# Loss of BAP31 Is Detrimentally Aging Photoreceptors Through ER Stress-Mediated Retinal Degeneration

**DOI:** 10.3390/cells14221802

**Published:** 2025-11-17

**Authors:** Fei Gao, Yuqiang Zheng, Tianyi Wang, Mingqi Zhang, Yuanlong An, Zhuoshi Wang, Bing Wang

**Affiliations:** 1 College of Life Science and Health, Northeastern University, Shenyang 110000, China; jluhpgaofei@126.com (F.G.); wangtianyi@mail.neu.edu.cn (T.W.); 2 Stem Cell Research Center, Precision Medical Innovation Institute, He University, Shenyang 110000, China; qiang_frank@163.com (Y.Z.); zhangmingqi111@163.com (M.Z.); anyuanlong@huh.edu.cn (Y.A.)

**Keywords:** BAP31, RNA sequencing, phototransduction, ER stress, UPR, retinal degeneration

## Abstract

Retinal degeneration (RD) is an intractable ophthalmic disorder with no effective treatments, and its pathogenesis is complex, involving multiple genes. Endoplasmic reticulum (ER) stress and neuronal apoptosis are key factors that drive neurodegeneration in retinal degeneration. B cell receptor-associated protein 31 (BAP31) is a transmembrane protein predominantly found in the ER, which plays an important role in regulating ER stress and apoptosis. To date, no studies have directly confirmed the association between BAP31 and retinal degenerative diseases. However, considering that ER dysfunction is a key trigger for retinal photoreceptor cell damage and that BAP31 acts as a core regulator of ER function, we hypothesize that BAP31 may be involved in the development of retinal degeneration by regulating ER homeostasis. Our study aimed to investigate the pathogenic mechanisms of BAP31 in retinal disorders. A rod-specific conditional knockdown of BAP31 mouse model (Rho-iCre-BAP31^fl/fl^(−/−)) was employed to explore the role of BAP31 in retinal pathogenesis. The Rho-iCre-BAP31^fl/fl^(−/−) mice exhibited phenotypes similar to retinitis pigmentosa (RP), including decreased ERG responses, photoreceptor degeneration, and reduced visual function. Optical coherence tomography (OCT) results showed that the outer nuclear layer (ONL) of the retina in conditional knockdown mice exhibited progressive thinning after 9 months of age; histopathological examination results were consistent with those of OCT. These findings indicated that the rod photoreceptor cells in the conditional knockdown mice showed damage and irregular arrangement starting at 9 months of age, with more prominent changes by 12 months. RNA sequence analysis of 12-month-old mice indicated enrichment of the phototransduction pathway, with significant downregulation of key genes (*rhodopsin*, *recoverin*, *Gnat1*, *Pde6a*, and *Pde6b*) involved in retinal development and phototransduction, along with a marked increase in *Gfap* expression (indicating glial activation and retinal damage). Quantitative real-time PCR and Western blot analyses showed significant upregulation of unfolded protein response (UPR) marker proteins (BIP, CHOP, XBP1, ATF4, ATF6), demonstrating robust ER stress activation. The findings suggest that BAP31 deficiency induces retinal degeneration, and the activation of the ER stress may contribute to the pathogenic mechanisms underlying this process.

## 1. Introduction

Retinal degeneration (RD) is known to be caused by apoptosis or the impaired function of photoreceptors. The main types of RD are retinitis pigmentosa (RP) and age-related macular degeneration (AMD). The main characteristics of RD are progressive loss of photoreceptors and impairment of visual function, ultimately leading to blindness. There are many genes and mechanisms reported to cause RD. However, there are currently no particularly effective treatments for this disease in clinical practice. Many studies have found that unfolded protein response (UPR) activation has played a role in several retinal degenerative diseases, such as inherited retinal degeneration (IRD), Stargardt disease, Leber congenital amaurosis, AMD, and diabetic retinopathy (DR) [1]. UPR activation has also been detected in many animal models. When UPR-related genes are knocked out in animal models, similar retinal degenerative diseases are produced [2]. For example, ATF6 is crucial for human cone photoreceptors [3]; its deficiency causes damage to both cone and rod cells, leading to retinal degenerative diseases. XBP1 plays a key role in photoreceptor synapses [4]; once absent, retinal neurodegeneration accelerates in diabetic patients.

The ER plays an important role in organisms. The main functions of the ER include protein biosynthesis, and post-translational modification, folding, and transporting. Consequently, the ER has consistently been regarded as the protein factory of cells. Moreover, the ER possesses the ability to detect and rectify abnormal protein folding states to prevent pathological occurrences. The dysfunction of the ER can give rise to endoplasmic reticulum stress and the subsequent activation of the UPR intracellular signal transduction network [5]. Chronic ER dysfunction can exert a significant influence on cells. The long-term activation of the UPR can result in cell death, inflammation, and oxidative stress, etc. [6,7] Numerous studies have indicated that many human diseases are associated with ER stress and the UPR, such as diabetes [8], cancer [9], vascular diseases [10], neurodegenerative diseases [11], etc. Among them, retinal diseases are mainly related to ER dysfunction [1,12,13,14].

B cell receptor-associated protein 31 (BAP31) is a widely expressed transmembrane protein that is localized in the ER. It is involved in regulating the synthesis, transportation, and degradation of multiple intracellular proteins and plays a crucial role in transporting protein from the ER to the Golgi apparatus. BAP31 is involved in the “ER quality control compartment”, which is a special perinuclear compartment [15], and has also been reported to be involved in ER homeostasis. Moreover, the absence of BAP31 will cause ER stress and activate the UPR [16], and this mechanism plays a core regulatory role in the pathological processes of various diseases. Previous studies by our team have revealed that BAP31 has many important functions, such as participating in the proteasome degradation pathway [17], T cell activation [18], insulin resistance [19], promoting apoptosis [20], activating autophagy [21], and the progression of Alzheimer’s disease, etc. [22]. In particular, previous research conducted by our team has shown that BAP31 has an inhibitory effect on neurodegenerative diseases. It can protect against neuroinflammation and the associated memory deficits [23]. Recently, our team has further expanded this understanding; we found that BAP31 can regulate superoxide levels in microglia by modulating the p22phox and Keap1/Nrf2/HO-1 signaling pathways, thereby participating in the pathological regulation of neurodegenerative diseases [24]. Qin et al. [25] provided a comprehensive elucidation of the core mechanism by which BAP31 mitigates neurodegenerative lesions, such as those observed in Parkinson’s disease, through the inhibition of ER stress-mediated apoptosis. This research offered substantial theoretical support for its neuroprotective role. Notably, this mechanism aligns closely with the established pathway of “excessive ER stress and neuronal apoptosis” seen in retinal degenerative diseases [26,27,28], thereby offering significant insights for investigating the function of BAP31 within the retina.

BAP31 plays an important role not only in the nervous system but also in metabolism and liver diseases. Studies have confirmed that BAP31 deficiency inhibits adipogenesis, blocks lipolysis, and promotes abnormal enlargement of lipid droplets by reducing the proteasomal degradation of Perilipin1, directly associated with the occurrence and development of lipid metabolism disorders [29]. In the context of liver health, relevant investigations have further elucidated the regulatory role of BAP31. For instance, one study constructed a hepatocyte-specific BAP31-deficient mouse model and found that BAP31 deficiency significantly exacerbates acetaminophen-induced hepatotoxicity by impairing Nrf2 signaling activation [30]. Another independent study complemented this finding by revealing that in chronic alcoholic liver injury, the deacetylase Sirtuin 2 can alleviate endoplasmic reticulum (ER) stress-induced hepatocyte apoptosis by deacetylating BAP31 [31]. The above studies suggest that BAP31, as a key molecule regulating the physiological functions of multiple systems and disease progression in the body, plays an important role in processes such as maintaining body homeostasis, regulating cellular metabolism, and mediating the occurrence and development of diseases.

In addition, BAP31 dysfunction mutations are the cause of “deafness, dystonia, and cerebral/central hypomelination” (DDCH) syndrome, which is characterized by severe neurological symptoms and early death. This disease is a rare autosomal dominant disorder, caused by missense mutations in the BAP31 gene, which is located in the q28 region of the X chromosome [32,33]. The pathological changes in DDCH patients mainly involve the auditory system and the central nervous system. However, no tests on systematic retinal function and structure have been conducted on these patients in existing reports. Although all these systemic diseases rely on capacity of BAP31 to sustain cellular homeostasis, such as regulating ER stress and facilitating protein folding, there is a lack of clear evidence on whether BAP31 mutations have effects on the retina. Notably, the retina is an organ with extremely high metabolic demands, and its phototransduction process also relies heavily on robust ER function. Age-related retinal diseases are the leading cause of irreversible blindness worldwide, and their pathogenesis is closely associated with the decline of cellular homeostasis, including impaired ER stress resolution, accumulation of misfolded proteins, and accelerated loss of photoreceptor cells [34]. These diseases share core pathogenic features with BAP31-related systemic diseases (such as ER stress, apoptosis, and protein misfolding), but whether BAP31 is involved in maintaining retinal health or retinal disease progression, and whether it can serve as a protective factor against age-related retinal degeneration, remains completely unknown.

Given the above, we hypothesize that BAP31 plays a crucial role in maintaining retinal homeostasis. To verify our hypothesis, we investigated the roles of BAP31 in rod cells by generating BAP31 rod-specific conditional knockdown mice (Rho-iCre-BAP31^fl/fl^(−/−)). We found that conditional knockdown of BAP31 in the retina induced ER stress, which led to a reduction in the ONL of the retina, apoptosis of photoreceptor cells, and ultimately impaired the visual function of the mice. At the same time, the expression of ER stress markers increased, and the misfolding of proteins in the retina increased. This result is highly consistent with the mechanism by which BAP31 regulates ER homeostasis to function in other systems, initially suggesting that BAP31 may be involved in retinal degenerative diseases through similar pathways. Additionally, in 12-month-old mice, the expression of glial fibrillary acidic protein (GFAP) was increased and gliosis was severe, indicating that BAP31 is crucial for the long-term survival of photoreceptor cells. This study not only preliminarily explored the potential mechanism by which BAP31 affects retinal function but also verified its role in retinal degenerative diseases. In turn, this provides a new direction for subsequent research and offers hope for identifying new therapeutic targets for age-related retinal diseases.

## 2. Materials and Methods

### 2.1. Animals and Genotyping

Conditional knockdown of BAP31 in the retina was achieved by crossing mice with LoxP sites flanking BAP31 [35] with a retina-specific Rho-iCre line [36]. The Rho-iCre line carries an improved Cre recombinase (iCre), which exhibits higher recombination efficiency than the traditional Cre recombinase and mitigates the risks of background recombination and non-specific expression. This line is widely used in the study of gene functions related to photoreceptor cells and eye diseases. The two independent lines have been interbred and maintained as BAP31^fl/fl^(+/+) mice and Rho-iCre-BAP31^fl/fl^(−/−). The F1 offspring were backcrossed to BAP31^fl/fl^(+/+) parental mice for more than 3 generations to obtain conditional knockdown mice (Rho-iCre-BAP31^fl/fl^(−/−)). During subsequent experimental procedures, sib-mating within the same genotype was performed every 2 generations. Meanwhile, genotyping was regularly conducted by PCR to ensure consistent genetic background and effectively prevent genetic drift. We also verified the expression level of BAP31 at different time points using Western blot, confirming that BAP31 was maintained at a low expression level (Figure S1).

Genotyping was performed by PCR using the primers lox-S-5’-GAGAAGCTAATGGTCTGTGACCCTGA-3′ and loxA-5′-CTACAGAGCAAGTGCCATGACATCC-3′, resulting in a 170 bp band for WT and a 298 bp band for BAP31 ^fl/fl^(+/+). The presence of the Rho-iCre allele was determined by PCR with Cre-F 5′-TCAGTGCCTGGAGTTGCGCTGTGG-3′ and Cre-R 5′-CTTAAAGGCCAGGGCCTGCTTGGC-3′, resulting in a 550 bp band. At each time point (3, 6, 9, and 12 months of age), there were 6–8 animals, with an equal number of males and females.

All animals were illuminated by fluorescent lamps with 12 h light/12 h dark cycle, and the ambient temperature was controlled at 25 ± 2 °C. All animals were treated in accordance with the Guide for the Care and Use of Laboratory Animals and the ARVO Statement for Use of Animals in Ophthalmic and Vision Research. They were monitored with approval from the Institutional Animal Care and Use Committee (IACUC) of He University.

### 2.2. Electroretinography (ERG)

All the mice were dark-adapted for 12 h prior to the start of ERG recordings, and all procedures were performed under dim red light. Mice were anesthetized with Tiletamine hydrochloride and Zolazepam hydrochloride (16 mg/mL) at a dose of 50–75 mg/kg, and 1% topical tropicamide was used to dilate the pupils. After the anesthesia, each mouse was placed on a test bench. The contact lens electrodes were placed on each cornea, reference electrodes were placed in the mouth, and the ground electrode was placed intradermally next to the tail. The a- and b-wave amplitudes were recorded at intensities of 0.015, 1.5, 3.0, and 10.0 cd·s/m^2^. The recordings were analyzed using Espion e2 software.

### 2.3. OCT

Mice were anesthetized via intraperitoneal injection of a ketamine (Jiangsu, China) and xylazine (Sichuan, China) mixture (100 mg/kg and 10 mg/kg, respectively). Retinal thickness was captured using rectangular volume scans, with the optic nerve serving as the positional reference. The outer nuclear layer (ONL) and retinal thickness were measured using the built-in calipers of the imaging instrument (Optoprobe Science LTD, London, UK) before the images were saved for further analysis. The thickness of the ONL measured by OCT at different age points was analyzed using two-way ANOVA with replicates for multiple comparisons.

### 2.4. Immunohistochemistry and Retinal Staining

The mice were euthanized, and eyeballs were enucleated. The eyes were fixed with 4% paraformaldehyde for 24 h at 4 °C. After removing the cornea and lens, the eyes were embedded in OCT and directly frozen. They were then sectioned at 8 µm thickness through the optic nerve head using a cryostat. The slices were taken from the −80 °C freezer and dried at room temperature for 10 min before soaking them in PBS to remove the OCT. They were fixed with 4% paraformaldehyde for 30 min, then blocked with 1% bovine serum albumin for 1 h at room temperature. The primary antibodies were diluted in accordance with the optimal dilution ratio and incubated the slices overnight at 4 °C. The following primary antibodies were used: RHO (abcam, ab98887, Mouse, 1:200, Cambridge, UK), GNAT1 (proteintech, Rabbit, 1:200, Rosemont, IL, USA), PDE6A (HUABIO, HA500264, Rabbit, 1:500, Hangzhou, China), PDE6B (santa, sc-377486, Mouse, 1:500, Dallas, TX, USA), GFAP (abcam, ab7260, Rabbit, 1:600, Cambridge, UK).

### 2.5. HE

The mice were euthanized, and eyeballs were enucleated. Immediately, the eyeballs were immersion fixed using Davidson’s fixative (PH0975, Phygene, Fuzhou, China) and placed in a refrigerator at 4 °C overnight. Before dehydration, the cornea and iris were removed. After gradient dehydration in a tissue dehydrator, the lens was removed and the tissue block was immersed in paraffin. Then, the embedded eyes were sliced into 6 um sections using a paraffin slicing machine (BIOCUT, Leica Microsystems, Wetzlar, Germany). The retina sections encompassing the optic nerve head (ONH) were carefully selected and subjected to hematoxylin–eosin (HE) staining for subsequent histological examination and analysis.

### 2.6. Western Blot Analysis

After the animals were euthanized, the eyeballs were enucleated and retinas were quickly isolated on ice. The retinas were washed with cold PBS and lysed using RIPA lysis buffer (Solarbio, Shanghai, China) supplemented with 1 mM phenylmethanesulfonyl fluoride (PMSF) and protease inhibitors for 30 min. Following protein quantification using the BCA Protein Assay Kit (Beyotime Institute of Biotechnology, Shanghai, China), 20 µg of protein was added to each lane and separated by 10% or 12% sodium dodecyl sulfate polyacrylamide gel electrophoresis (SDS-PAGE). Then, the samples were transferred to polyvinylidene fluoride (PVDF) transfer membranes (Merck KGaA, Darmstadt, Germany) and blocked by blocking buffer for 1 h. PVDF membranes containing specific proteins were then carefully incubated in primary antibodies overnight at 4 °C. The following day, PVDF membranes were washed and incubated with the corresponding HRP-conjugated secondary antibody for 1 h at room temperature. The immunoreactivity was detected using an ECL Kit (Beyotime Institute of Biotechnology, Shanghai, China). ImageJ software (ImageJ1.47, Media Cybernetics, Baltimore, MD, USA) was used to assess the protein levels, which were normalized to the relative density of tubulin. The following primary antibodies were used: BAP31 (our lab, Rabbit, 1:3000, Shenyang, China), Cre (abcam, ab216262, Rabbit, 1:2000, Cambridge, UK), RHO (abcam, ab98887, Mouse, 1:1000, Cambridge, UK), RCVRN (Abways, *AY3894*, *Rabbit*, *1:1000*, Shanghai, China), GNAT1 (proteintech, Rabbit, 1:2000, Rosemont, USA), PDE6A (HUABIO, HA500264, Rabbit, 1:500, Hangzhou, China), PDE6B (santa, sc-377486, Mouse, 1:500, Dallas, USA), CRX (abcam, ab140603, Rabbit, 1:1000, Cambridge, UK), GFAP (abcam, ab7260, Rabbit, 1:10000, Cambridge, UK), BIP (proteintech, 66574-1-Ig, Mouse, 1:2000, Rosemont, USA), ATF4 (beyotime, AF2560, Rabbit, 1:1000, Shanghai, China), ATF6 (Affinity, DF6009, Rabbit, 1:1000, Cinti, OH, USA), XBPI (HUABIO, ET1703-23, Rabbit, 1:500, Hangzhou, China), CHOP (HUABIO, HA722854, Rabbit, 1:500, Hangzhou, China), and TUBULIN (abcam, ab18207, Rabbit, 1:5000, Cambridge, UK).

### 2.7. Optomotor Response Test

The visual function was measured by an automated head tracking system, according to previously published methods [37]. The mice were adapted to darkness for 12 h in advance. In a dark and quiet environment, the mice were placed on the central platform of the machine where they were allowed to move freely. After starting the detection, the mice were presented with vertical black and white stripes generated by the surrounding LCD monitors located around the machine in a random clockwise or counter-clockwise cylindrical image. We selected four different spatial frequencies (0.1, 0.15, 0.2, and 0.3 cycles/degree) or presented the best spatial frequency (0.2 cycles/degree) with five different contrast levels (100%, 50%, 25%, 12.5%, and 5%). The head movements were monitored using a camera.

### 2.8. Quantitative Real-Time PCR

Total RNA was isolated from the retinas using TRIzol^®^ Reagent (Ambion, Austin, TX, USA). Subsequently, the extracted RNA was reverse-transcribed into complementary DNA (cDNA) using a cDNA synthesis kit (BioFlux, Hangzhou Bioer Technology Co., Ltd., Hangzhou, China). For quantitative real-time PCR, SYBR Green qPCR Master Mix (BioFlux, Hangzhou Bioer Technology Co., Ltd., Hangzhou, China) was used, with the cycle time, temperature, and number of cycles set in accordance with the manufacturer’s protocol. The reaction was performed in qTOWER^3^G (Analytik Jena, Jena, Germany). The sequences of the primers used for qPCR are provided in Table S1.

### 2.9. RNA Sequencing

Following inhalation anesthesia, the eyeballs of the mice were promptly enucleated. The corneas were incised, and the retinas were quickly dissected after removing the lenses. All procedures were carried out on ice to ensure sample integrity. Total RNA was extracted using Trizol Reagent (Invitrogen Life Technologies, Waltham, MA, US), and its concentration, quality, and integrity were assessed using a NanoDrop spectrophotometer (Thermo Scientific, Waltham, MA, USA). Three micrograms of RNA were used as the starting material for RNA sample preparation. Sequencing libraries were constructed following standard operating procedures (SOP) and subsequently sequenced on the NovaSeq 6000 platform (Illumina, San Diego, CA, USA) at Shanghai Personal Biotechnology Co., Ltd. (Shanghai, China).

Samples were sequenced to obtain image files, which were transformed by the software of the sequencing platform. The original data was generated in FASTQ format (Raw Data). Sequencing data contains a number of connectors and low-quality reads, so we filtered it using the fastp (0.22.0) software to obtain high-quality sequence data (Clean Data) for further analysis. The reference genome and gene annotation files were downloaded from the genome website. The filtered reads were mapping to the reference genome GRCm39 (Ensembl 108.39) using HISAT2 (v2.1.0). We used HTSeq (v0.9.1) statistics to compare the Read Count values on each gene as the original expression of the gene, and then used FPKM (Fragments Per Kilo bases per Million fragments)/TPM (Transcripts per Million) to standardize the expression. The difference in expression of genes was analyzed by DESeq2 (v1.38.3), with screened conditions as follows: expression difference multiple |log2FoldChange| > 1, significant *p*-value < 0.05. At the same time, we used the ComplexHeatmap (v2.16.0) software package to perform bi-directional clustering analysis of all different genes in the samples. We generated a heatmap according to the expression level of the same gene in different samples and the expression patterns of different genes in the same sample, using the Euclidean method to calculate the distance and the Complete Linkage method to cluster.

### 2.10. Statistical Analysis

The GraphPad Prism 5 software was used to analyze the dates obtained from at least three independent experiments. A two-tailed Student’s *t*-test was used for comparison. Before conducting the two-tailed Student’s *t*-test, the Shapiro–Wilk test was performed on each group of data using GraphPad Prism 5.0 software. The test results for all groups met the criterion of *p* > 0.05, indicating that the data conformed to a normal distribution. Each group of data was presented as mean ± SEM. * *p* < 0.05, ** *p* < 0.01, and *** *p* < 0.001 were considered statistically significant.

## 3. Results

### 3.1. Deletion of BAP31 in Mouse Rod Photoreceptors via Rho-iCre

**Figure 1:**
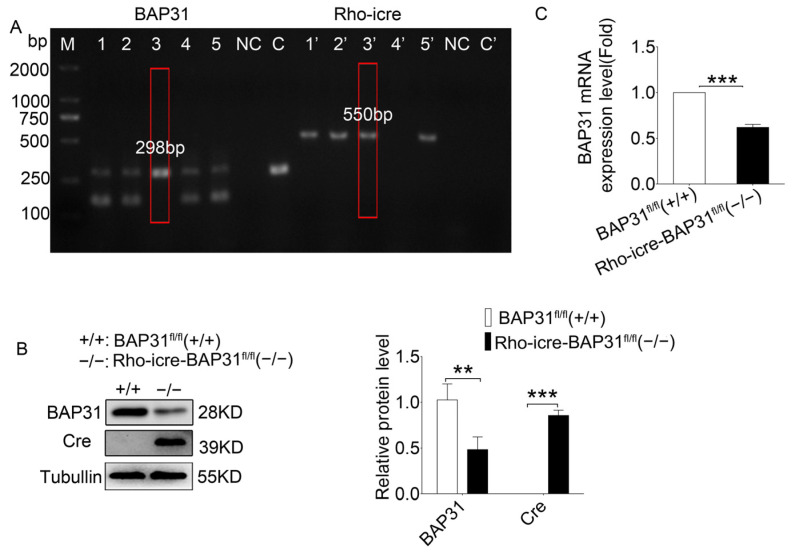
Deletion of BAP31 in mouse rod photoreceptors via Rho-iCre. (**A**) At one month of age, genotyping was conducted using mouse tail PCR. The *Bap31*-specific primer generated a single amplification product of 298 bp, whereas the Rho-Cre-specific primer yielded a single band of 550 bp. Animals exhibiting both distinct bands were identified as model animals, which we named Rho-iCre-BAP31^fl/fl^(−/−) (as indicated by a red rectangular box) (lane 3 and 3’). NC: negative water control; C and C’: BAP31^fl/fl^(+/+) mouse control. (**B**) Western blot analysis was performed to assess the expression levels of BAP31 and Cre proteins in the retinas of BAP31^fl/fl^(+/+) mice and Rho-iCre-BAP31^fl/fl^(−/−) mice (*n* = 3). (**C**) The mRNA level of *Bap31* was measured using real-time PCR analysis (*n* = 3). ** *p* < 0.01, *** *p* < 0.001.

To determine the role of BAP31 in photoreceptor development and homeostasis, we generated BAP31 conditional knockdown mice. We crossed the BAP31^flox/flox^ to the Rho-iCre (Cre) line to specifically delete BAP31 in rod photoreceptors. We used PCR to identify the recombination of mice (Figure 1A). The results showed that the genetic background required for BAP31 recombination (i.e., the presence of floxed BAP31 and Cre recombinase) was successfully established. Experimental mice with the correct genotype exhibited simultaneous amplification of a single 298 bp band for *Bap31* and a single 550 bp band for *Cre* (lanes 3 and 3’). Mice with incorrect genotypes exhibited two types of amplification results: one consisting of two bands (298 bp and 170 bp) amplified by *Bap31* primers plus one 550 bp band for *Cre* primers (lanes 1, 2, 5 and 1’, 2’, 5’), and the other consisting of two bands (298 bp and 170 bp) amplified by *Bap31* primers with no band amplified for *Cre* primers (lanes 4 and 4’). Among these, the bands at 178 bp and 298 bp correspond to the amplification products of the unfloxed and floxed *Bap31* alleles, respectively; the 550 bp band indicates the expression of Cre recombinase. To further confirmed the occurrence of recombination and the consequent knockdown of BAP31, we performed Western blot (Figure 1B) and qPCR (Figure 1C) analyses. Notably, rod cells constitute approximately 70% of all cells within the mouse retina. This significant proportion ensures that the protein and mRNA expression levels observed in whole retinal lysates are highly representative of the expression profiles found in rod cells. Consequently, we utilized whole retinal extracts for our detection purposes. Western blot results showed significant decreases in BAP31 in the correct genotype mice retina and the Cre protein only expressed in the correct genotype mice retina (Figure 1B). The results of qPCR demonstrated a significant downregulation of *Bap31* mRNA in the retina of correct genotype mice, which was consistent with the protein expression profile observed in the Western blot analysis, indicating a concordant reduction in both transcriptional and translational levels of BAP31.

### 3.2. BAP31-Specific Knockdown Results in Age-Related Degeneration

**Figure 2:**
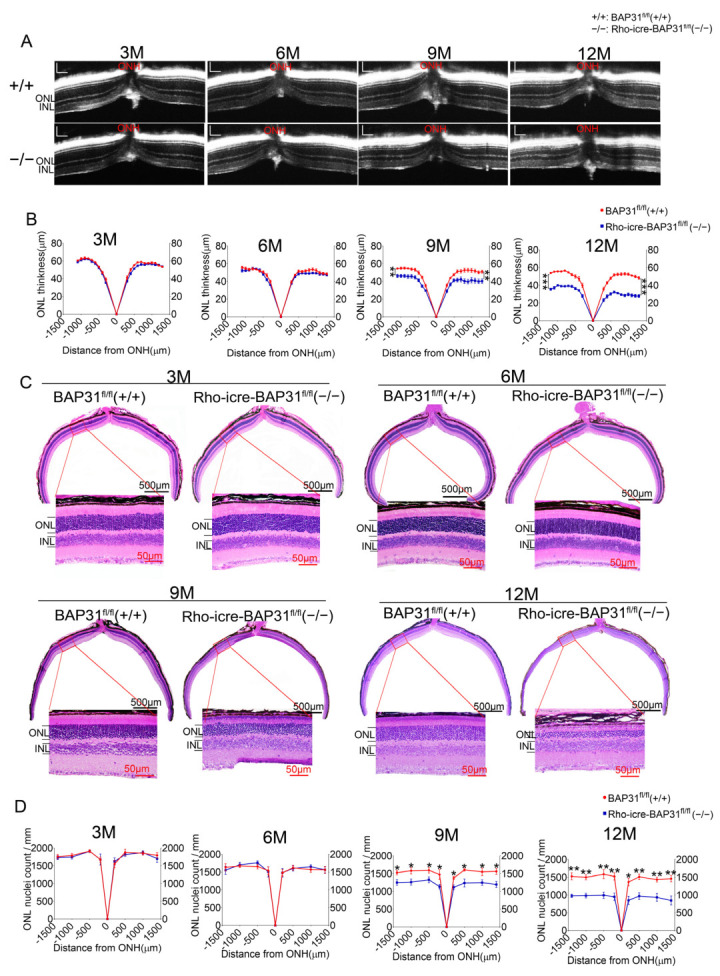
BAP31-specific knockdown results in age-related degeneration. (**A**) In vivo OCT images of BAP31-deficient retinas at 3, 6, 9, and 12 months of age demonstrated progressive thinning of the retinal layers starting from 9 months of age. (*n* = 8). Horizontal scale bar = 248.5 μm, vertical scale bar = 150 μm.(ONL outer nuclear layer; INL inner nuclear layer; ONH optic nerve head) (**B**) ONL thickness was quantified in both BAP31^fl/fl^(+/+) mice and Rho-iCre-BAP31^fl/fl^(−/−) mice at the age of 3, 6, 9, 12 months (*n* = 8). (**C**) Paraffin sections were stained with H&E at 3, 6, 9, and 12 months of age of BAP31^fl/fl^(+/+) mice and Rho-iCre-BAP31^fl/fl^(−/−) mice (*n* = 6). (**D**) The linear density of nuclei in the ONL at 3, 6, 9, and 12 months of age of BAP31^fl/fl^(+/+) mice and Rho-iCre-BAP31^fl/fl^(−/−) mice. The gradual decrease in nuclear linear density in the ONL suggested the progressive loss of the number of photoreceptor cells. (*n* = 6).* *p* < 0.05, ** *p* < 0.01, *** *p* < 0.001.

To investigate the impact of BAP31 deficiency on retinal development, we conducted a long-term study in mice over a period of 12 months. We analyzed retina using OCT imaging at different time points (Figure 2A). No abnormalities were observed in the retinas of BAP31-deficient mice at early time points (3 and 6 months) using OCT imaging. We detected a persistent thinning of retinal thickness at 9 months. This further intensified at 12 months in Rho-iCre-BAP31^fl/fl^(−/−) mice, reaching approximately 60% of that observed in BAP31^fl/fl^(+/+) mice (Figure 2B). Notably, no significant differences in the thickness of the inner nuclear layer (INL) were recorded throughout the experimental period (Figure S2A). In contrast, changes in total retinal thickness mirrored those seen in ONL thickness (Figure S2B). This indicates that overall retinal thinning resulting from BAP31 deficiency was primarily attributed to ONL thinning. Histological analysis of the retinas was consistent with OCT results, with no abnormalities detected at 3 and 6 months, but the thickness of ONL was significantly reduced at both 9 and 12 months of age (Figure 2C, Table S2). Further verification by the analysis of nuclear linear density in the ONL (Figure 2D) revealed that, starting from 9 months, the number of ONL cells in Rho-iCre-BAP31^fl/fl^(−/−) mice was significantly lower than that found in BAP31^fl/fl^(+/+) controls. Given that the ONL is rich in photoreceptor cells—key components for visual signal transmission—this result suggested that BAP31 deficiency may lead the loss of the photoreceptors.

The above results demonstrated that the deletion of BAP31 had no influence on the early development of mouse rod photoreceptor cells, but caused loss of rod photoreceptor cells after the age of 9 months, indicating an age-dependent pattern of this phenomenon. Consequently, there was a significant reduction in ONL thickness and photoreceptor nuclear density, which implies potential impairment of rod photoreceptor survival.

### 3.3. Absence of BAP31 in Rod Photoreceptors Impaired the Visual Function Response (ERG) with Age

**Figure 3:**
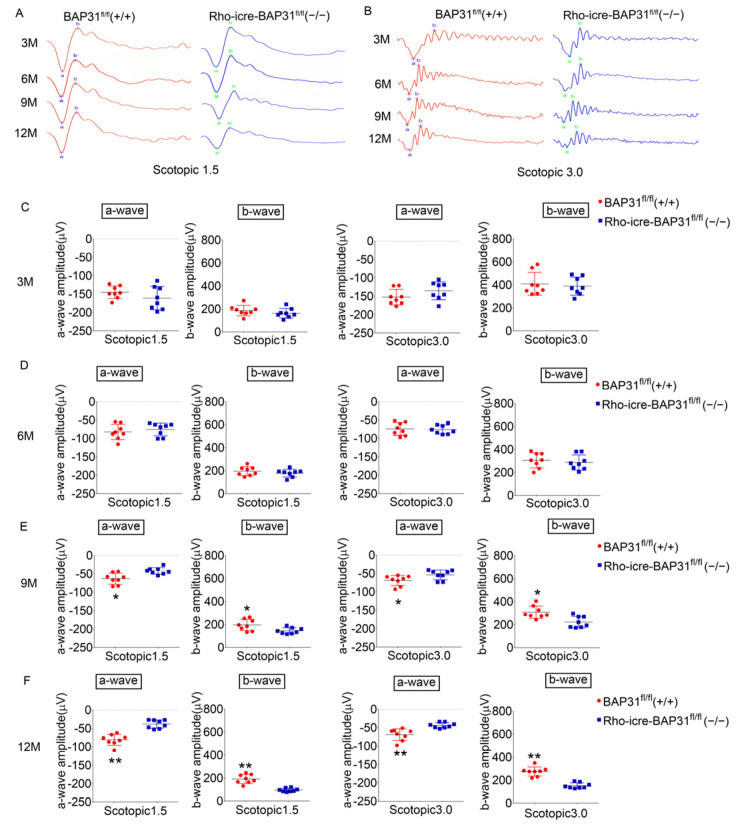
Electroretinography (ERG) recording analysis. The ERG waveforms were recorded of retinas of BAP31^fl/fl^(+/+) mice and Rho-iCre-BAP31^fl/fl^(−/−) mice at 3, 6, 9, and 12 months of age under 1.5 flash strength (cd·s/m^2^) (**A**) and 3.0 flash strength (cd·s/m^2^) (**B**) (*n* = 8). (**C**–**F**) Quantitative analysis of a-wave and b-wave amplitudes was conducted at flash strengths of 1.5 and 3.0 cd·s/m^2^. Statistical comparisons were performed to assess potential differences between the genotypes. * *p* < 0.05, ** *p* < 0.01.

We employed ERG to examine the visual function of the mouse models in order to verify whether the conditional deletion of BAP31 in rod photoreceptor cells would result in visual disorders. We conducted two types of ERG tests: scotopic ERG for evaluating rod and cone function, and photopic ERG for the selective assessment of cone photoreceptor function. The ERG waveforms of BAP31^fl/fl^(+/+) control mice and Rho-iCre-BAP31^fl/fl^(−/−) mice varied under different stimulation energies (Figure 3A,B). Statistical analysis of the ERG results from the two groups of mice under scotopic 1.5 and 3.0 cd·s/m^2^ energy stimulations revealed that the mean amplitudes of a-waves and b-waves in 3- and 6-month-old mice showed no statistically significant differences, indicating that the visual dysfunction was not detected at these stages (Figure 3C,D). However, at the age of 9 months, dark-adapted ERG recordings demonstrated a significant reduction in both a-wave and b-wave amplitudes in the BAP31-deficient mice compared to the control group (Figure 3E). The decline in retinal function progressively worsened by 12 months of age, with a significant reduction observed in both a-wave and b-wave amplitudes. This result indicated a severe impairment of retinal function (Figure 3F). The a-wave primarily reflected the hyperpolarization of photoreceptors (rods and cones) in response to light, and the b-wave was generated primarily by the activity of bipolar cells, which were postsynaptic to the photoreceptors. This wave reflects the signal transmission from photoreceptors to bipolar cells and the subsequent depolarization of ON-bipolar cells, providing insights into the functional integrity of the inner retinal circuitry. Our experimental findings suggested that the reduction in the b-wave was likely a secondary consequence of diminished signal input from rod cells. However, whether there is also secondary damage to the inner retinal layers necessitates further comprehensive investigation.

We also applied lower-intensity (0.15 cd·s/m^2^) and higher-intensity (10.0 cd·s/m^2^) stimuli to the two groups of mice, and the results were consistent with those obtained under 1.5 cd·s/m^2^ and 3.0 cd·s/m^2^ intensity stimuli (Figure S3). Results of photopic ERG (Figure S4A,B) showed that there were no statistical differences between mice at different time points; moreover, after 9 months of age, the photopic ERG response waveforms of the mice were extremely weak, suggesting that the cone cell function of mice at this age had basically declined. This result indicated that the functional retinal defects in mice at this stage were mainly caused by the loss or dysfunction of rod cells. In addition, we analyzed the variation in ERG waveforms with age and found that they became more obvious as age increased under stimuli of different intensities. ERG testing demonstrated a significant difference in the b-wave response between stimulus intensities of 3.0 cd·s/m^2^ and 10.0 cd·s/m^2^ under conditions of dark adaptation. We hypothesize that this phenomenon is linked to dysfunction in bipolar cell responses; however, it remains uncertain whether this dysfunction arises from diminished afferent signals from rod photoreceptors or if pathological changes have already affected the bipolar cells themselves. The underlying mechanisms require further investigation for clarification. Additionally, we analyzed the age-related changes in ERG waveforms (Figure S4C–G), focusing on the amplitude variations in the a-wave and b-wave in the ERG of the two mouse groups under different ages. The results showed that under scotopic conditions, both groups exhibited a decrease in ERG responses at age of 6 months. This decrease is hypothesized to be a physiological change. After 6 months, the ERG waveforms of the BAP31^fl/fl^(+/+) mice remained stable; however, starting from 9 months of age, the Rho-iCre-BAP31^fl/fl^(−/−) mice showed a continuous decline in ERG responses across different stimulus intensities. This indicated that BAP31 gene knockdown leaded to age-dependent progressive degeneration of retinal photoreceptor function (particularly rod photoreceptor function) in mice, with 9 months of age being a critical time point for functional impairment. A consistent degenerative trend was observed across all stimulation intensities tested.

Under photopic conditions, both the model and control mice displayed age-dependent progressive degeneration. This indicated that cone photoreceptors undergo age-related degeneration in both mouse groups, and after 9 months of age, the function of cone photoreceptors became very weak. Therefore, the degeneration of visual function was mainly driven by rod photoreceptors. All the observations indicate the critical role of BAP31 in maintaining the long-term functional integrity of photoreceptors, which is consistent with the progressive retinal degeneration observed in BAP31-deficient mice. These findings highlight the important role of BAP31 in maintaining retinal homeostasis and its potential involvement in age-related retinal diseases.

This observation emphasizes the essential role of BAP31 in sustaining the long-term functional integrity of photoreceptors, which aligns with the progressive retinal degeneration noted in BAP31-deficient mice. These findings underscore the critical role of BAP31 in maintaining retinal homeostasis and its potential involvement in age-related retinal pathologies.

### 3.4. BAP31 Deficiency in Rod Photoreceptors Weakened Visually Driven Optomotor Responses

**Figure 4:**
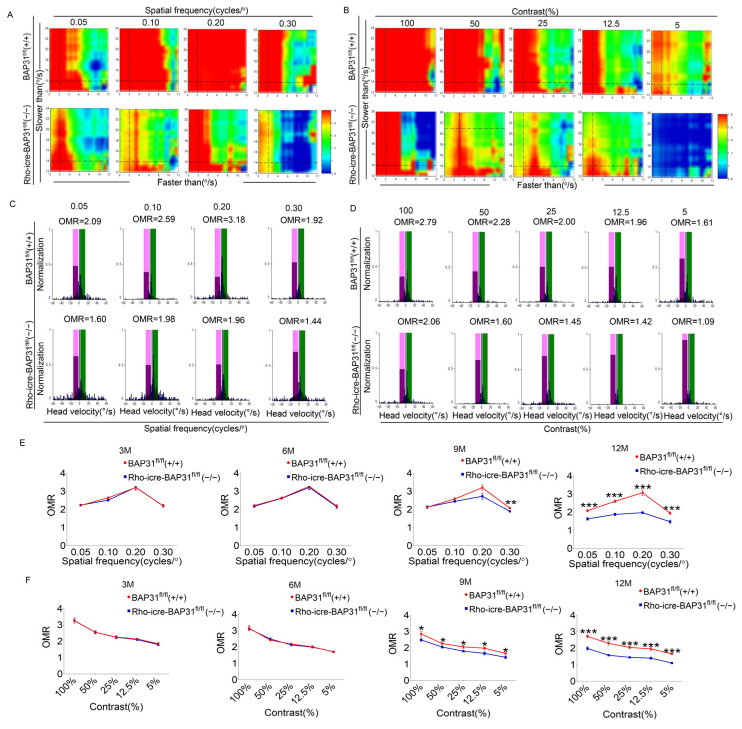
Visual impairment determined by visually driven optomotor behavior test in mice. (**A**) Compared with the control mice, Rho-iCre-BAP31^fl/fl^(−/−) mice at 12 months of age showed poorer responses to visual stimuli under a series of conditions with defined spatial frequencies (**A**) and contrasts (**B**) (*n* = 8). Representative heat maps indicate the wide range of OMR values within the defined spatial frequency or contrast levels (**A**,**B**). The head rotation speed was normalized using software to calculate the OMR ratio (**C**,**D**) (*n* = 8). (**E**,**F**) Statistical analysis of the differences in OMR values between Rho-iCre-BAP31^fl/fl^(−/−) mice and BAP31^fl/fl^(+/+) mice at 3, 6, 9, and 12 months of age under different stimulation conditions. Statistical tests are presented as mean ± SEM (*n* = 8 for each group); * *p* < 0.05; ** *p* < 0.01; *** *p* < 0.001.

Many studies have shown that if the retina of a mouse remains intact, it will have good visual function and respond to different spatial frequencies and contrasts. We accurately and objectively assessed visual function through a video tracking algorithm and quantitatively determined the degree of visual function in mice at 3, 6, 9, and 12 months of age. We used the OMR detection system. This system can reliably and impartially detect the changes in optokinetic responses of mice under different stimulation conditions. It has been reported that mice can hardly detect at 0.4 cycles/° [38]. Therefore, the spatial frequencies set in this experiment were 0.05, 0.1, 0.2 and 0.3 cycles/°. Representative heat maps were conducted on different ranges of velocity thresholds under specific spatial frequencies or contrast conditions. Figure 4A presents the representative heatmaps of the detection results for 12-month-old mice, in which red denotes relatively high OMR values and blue represents relatively low values. Across most velocity thresholds, BAP31^fl/fl^(+/+) mice sustained elevated OMR levels (≥2), with a peak observed at a spatial frequency of 0.2 cycles/°, where the OMR value exceeded 3. The visually stimulated responses of the Rho-iCre-BAP31^fl/fl^(−/−) mice were relatively low (<2) (Figure 4A). By comparing different spatial frequencies, it was found that the optokinetic response of mice was the most pronounced at 0.2 cycles/°. Therefore, a spatial frequency of 0.2 cycles/° was set, and different contrasts were adjusted. Figure 4B showed the representative graph of the detection results of 12-month-old mice. In terms of contrast, the visually evoked driving responses in BAP31^fl/fl^(+/+) mice peaked at 100% contrast, followed by a gradual decline starting from 50% contrast, extending down to the 5% contrast level. The Rho-iCre-BAP31^fl/fl^(−/−) mice exhibited a consistent trend in response changes compared to the control group; nevertheless, under the 100% contrast condition, their OMR values were already lower than those of the control mice (Figure 4B). A comparative analysis of visually evoked stimulus-driven responses was performed under optimal velocity conditions. Figure 4C,D show the representative graph of the detection results of 12-month-old mice. For BAP31^fl/fl^(+/+) mice, the OMR in the stimulus direction (positive value, light green window) exhibited significantly higher occurrence frequency and prolonged duration (blue bars) at 0.2 cycles/°, resulting in a peak OMR value of 3.18 at this spatial frequency. Correspondingly, the OMR values at 0.05, 0.1, and 0.3 cycles/° were 2.09, 2.59, and 1.92, respectively. In contrast, the Rho-iCre-BAP31^fl/fl^(−/−) mice displayed OMR values of 1.60, 1.98, 1.96, and 1.44 at 0.05, 0.1, 0.2, and 0.3 cycles/°, respectively (Figure 4C). With respect to contrast, the OMR value of BAP31^fl/fl^(+/+) mice reached a maximum of 2.79 at 100% contrast, and exhibited a steady decline as the contrast level decreased. The Rho-iCre-BAP31^fl/fl^(−/−) mice demonstrated a consistent pattern of OMR value changes relative to the control group (Figure 4D). Combined with the normalized results of different spatial frequencies and contrasts, the changing trend in OMR values was basically consistent with the characteristics presented in the heatmaps of Figure 4A,B. We further performed statistical analysis on the OMR results of mice at different ages (Figure 4E,F). At 3–6 months of age, no statistically significant differences in OMR values were observed between Rho-iCre-BAP31^fl/fl^(−/−) mice and control mice across all tested spatial frequencies and contrast conditions. By 9 months of age, the BAP31-deficient group showed significantly lower OMR values than the control group at spatial frequencies of 0.2 and 0.3 cycles/° and all contrast conditions. At 12 months of age, significant differences in OMR values were detected between the two groups across every tested spatial frequency and contrast conditions. These findings clearly illustrated the age-related pattern of OMR decline in mice caused by BAP31 deficiency. Specifically, mice develop functional deficits at specific frequencies/contrasts at 9 months of age, and by 12 months of age, these deficits further progress to widespread visual behavioral impairments. Notably, this degenerative process was synchronized with the retinal structural and visual function damages previously observed via OCT and ERG.

### 3.5. Loss of BAP31 Affects Phototransduction in the Retina

**Figure 5:**
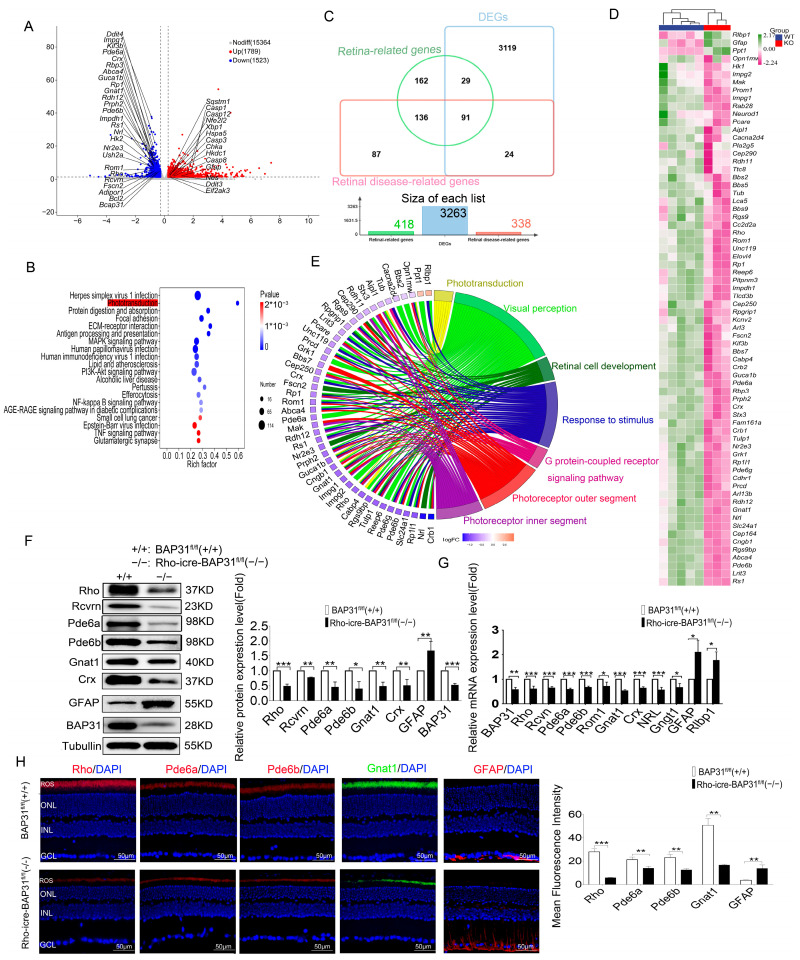
Loss of BAP31 affects phototransduction in the retina. (**A**) Volcano plot representation of differentially expressed genes between BAP31^fl/fl^(+/+) (*n* = 5) and Rho-iCre-BAP31^fl/fl^(−/−) (*n* = 3) retinas at 12 months of age. (**B**) KEGG pathway enrichment analysis of differentially expressed genes in the retina between BAP31^fl/fl^(+/+) (*n* = 5) and Rho-iCre-BAP31^fl/fl^(−/−) (*n* = 3) mice. The top 20 significantly enriched pathways are displayed.The phototransduction pathway is highlighted in red. (**C**) Intersecting genes associated with the retina in DEGs, retina-related genes of GeneCards database and RetNet database. (**D**) Heatmap showing the expression profiles of 71 differential genes with FPKM values greater than 10 among 91 intersecting genes. (**E**) Chord plot representation of differentially expressed genes (DEGs) associated with GO annotations for biological processes (BP) and cellular components (CC). The plot illustrates the relationships between DEGs (segments on the left) and their corresponding enriched biological processes (segments on the right). (**F**) Western blot analysis was performed to assess the expression levels of the phototransduction genes (*n* = 3). (**G**) Real-time PCR analysis showed the mRNA level of phototransduction genes. (BAP31^fl/fl^(+/+) = 5, Rho-iCre-BAP31^fl/fl^(−/−) = 3). (**H**) Immunofluorescence of retinal tissue for RHO, PDE6A, PDE6B, GNAT1, and GFAP expression and the mean fluorescence intensity analysis (*n* = 3). Scale bar = 50 μm.* *p* < 0.05; ** *p* < 0.01; *** *p* < 0.001.

To investigate the effect of BAP31 deletion on the retina, we isolated mouse retinal tissues from 12-month-old BAP31^fl/fl^(+/+) (*n* = 5) and Rho-iCre-BAP31^fl/fl^(−/−) (*n* = 3) mice and subjected them to RNA sequence analysis. A total of 3265 differential genes were identified, of which 1746 were upregulated and 1519 were downregulated (|FC| ≥ 1.2 *p* < 0.05) (Figure 5A). The results of KEGG enrichment analysis revealed that the phototransduction pathway was significantly enriched and positioned among the top-ranked pathways (Figure 5B). Downwards regulation in the phototransduction pathway (Figure S5) was clearly evident. The genes of this pathway were expressed in the photoreceptor cilia. The disruption of this pathway is the typical characteristic of stereotypical degeneration in RP. According to the latest statistics from RetNet (https://web.sph.uth.edu/RetNet/(accessed on 2 March 2025)), there are 338 genes associated with retinal diseases in total (Table S3), while 418 retina-related genes (with a relevance score ≥ 7) were extracted from the GeneCards database (Table S4). In this study, an intersection analysis was performed between these two gene lists and the 3263 differential genes obtained from transcriptome sequencing, and a total of 91 differential genes were screened out (Figure 5C). A heatmap analysis was performed on the differentially expressed genes with FPKM values greater than 10. In the heatmap, each column represents a single retinal sample, where green indicates upregulated genes and purple indicates downregulated genes (Figure 5D). We systematically analyzed the expression profiles of rod and cone photoreceptor-specific markers in our RNA sequencing dataset. We focused on two types of cell-specific markers: *rhodopsin* (*Rho*), a well-recognized marker for rod photoreceptors, and cone-specific opsins (S-opsin encoded by *Opn1sw*, L/M-opsin encoded by *Opn1mw*). We compared the expression levels of these markers between the control group and the model group. Results showed that the mRNA expression level of *Rho* in the Rho-iCre-BAP31^fl/fl^(−/−) group was significantly downregulated, with a relative expression of 0.55-fold compared to the control group (*p* < 0.01). This significant reduction strongly suggests that rod photoreceptor function may be impaired in the model group. In contrast, the relative expression of *Opn1sw* in the Rho-iCre-BAP31^fl/fl^(−/−) group was 0.83-fold that of the control group (*p* < 0.05), but there was substantial individual variation. The *Opn1mw* showed a slight upregulation (1.1-fold relative to the control group) without statistical significance (*p* > 0.05). These results indicated that cone photoreceptor markers did not undergo significant changes under our experimental conditions, further confirming the presence of rod photoreceptor-specific defects in the model.

To further investigate the impact of conditional deletion of BAP31 on retinal function, we performed Gene Ontology (GO terms) analysis on the screened candidate genes. These analyses aimed to elucidate the biological processes, molecular functions, and cellular components significantly affected by BAP31 deficiency in the retina. The results showed that the candidate genes were mainly associated with phototransduction, visual perception, retinal photoreceptor cell development, response to stimulus, G protein-coupled receptor signaling pathway, photoreceptor outer segment, and photoreceptor inner segment (Figure 5E). The connecting chords in the network diagram represent the strength of associations between differentially expressed genes (DEGs) and specific biological processes, with thicker chords indicating a greater number of DEGs linked to a particular process. This visualization underscores the key biological processes significantly influenced by the differential gene expression observed between BAP31^fl/fl^(+/+) and Rho-iCre-BAP31^fl/fl^(−/−) retinas. RNA sequencing results show that, among the 27 genes in the phototransduction pathway, 16 had significant change, with 15 genes downregulated and 1 upregulated. This indicates that the phototransduction pathway is significantly affected in the model animals, which may be the key factor contributing to visual dysfunction. The genes downregulated in the phototransduction pathway were *Rho*, *Rcvrn*, *Grk1*, *Gnat1*, *Gnb1*, *Gngt1*, *Pde6a*, *Pde6b*, *Pde6g*, *Gucy2e*, *Gucy2f*, *Guca1b*, *Slc24a1*, *Cngb1*, and *Rgs9*. All the genes played major roles in phototransduction and retinal degeneration. *Rho, Gnat1, Pde6a* and *Pde6g* are well-known genes involved in the rod phototransduction pathway [39]. The qPCR results showed a significant downregulation of the key genes related to photoreceptors and the retina in Rho iCre BAP31^fl/fl^(−/−) mice compared to BAP31^fl/fl^(+/+) (Figure 5F), including *Rde6a, Rde6b, Rho, Rcvrn, Rom1, Gnat1, Crx, Gngt1, Crx, Nrl, Gfap* and *Rlbp1*. The Western blot (WB) analysis further corroborated this observation, which shoed a substantial reduction in the protein expression levels of RHO, RCVRN, PDE6A, PDE6B, GNAT1, CRX and GFAP in Rho iCre BAP31^fl/fl^(−/−) mice relative to BAP31^fl/fl^(+/+) (Figure 5G). Immunofluorescence staining demonstrated that the staining intensity of RHO, PDE6A, PDE6B, and GNAT1 were significantly reduced in Rho-iCre-BAP31^fl/fl^(−/−) mice. In contrast, the expression level of GFAP was significantly increased (Figure 5H). All findings collectively indicate that the loss of BAP31 in Rho-iCre-BAP31^fl/fl^(−/−) mice disrupted the expression of critical retinal genes and proteins, potentially contributing to retinal dysfunction and impaired visual cycle regulation. This shows that the phototransduction pathway was significantly affected.

### 3.6. BAP31 Regulated UPR to Affect the Expression of Phototransduction Genes

**Figure 6:**
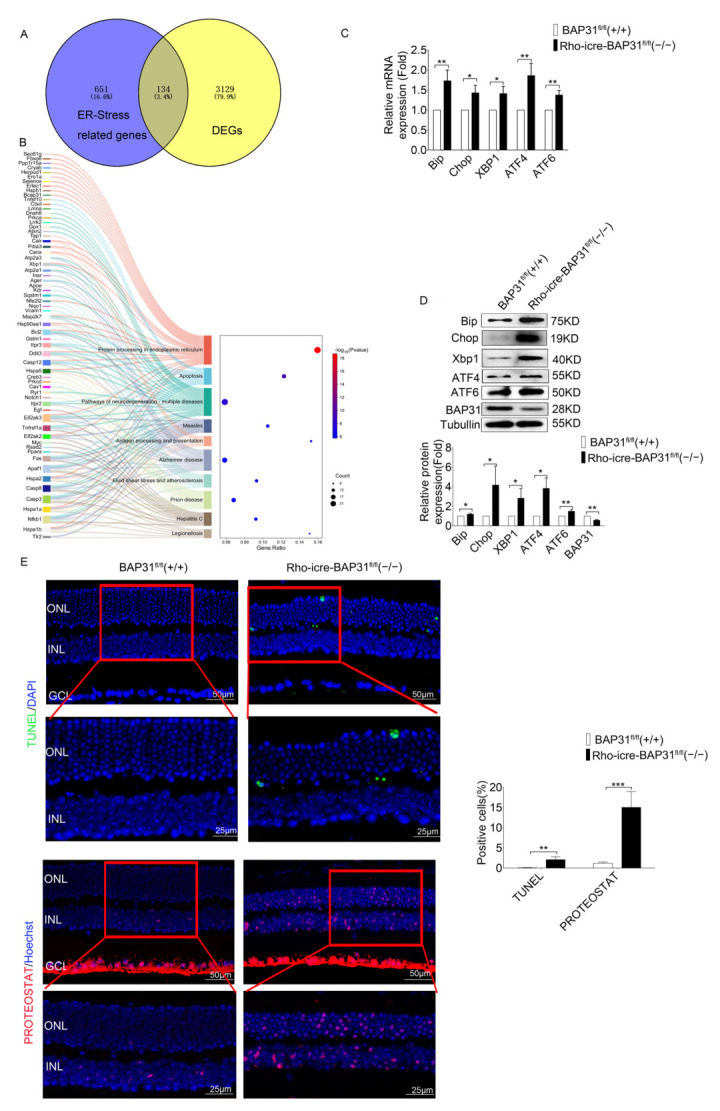
BAP31 affects the expression of phototransduction genes in the retina of 12-month-old mice by regulating UPR. (**A**) Intersecting genes associated with ER-stress related genes in DEGs and GeneCards database. (**B**) KEGG enrichment analysis on the selected genes. (**C**) The mRNA level of UPR genes were performed by real-time PCR analysis (*n* = 3). (**D**) Western blot analysis was performed to assess the expression levels of the UPR genes (*n* = 3). (**E**) Retinal cryosections were labeled by TUNEL (green) or PROTEOSTAT (red) (*n* = 3) Zoom-in pictures were shown in the red rectangle ((Scale bars = 50 μm low-magnification images) and scale bars = 25 μm (high-magnification images)). * *p* < 0.05; ** *p* < 0.01; *** *p* < 0.001.

The results demonstrated that the genes in the phototransduction pathway are significantly affected in the retinas of model animals with BAP31 deficiency; however, the precise mechanisms involved in this pathway remain elusive. To further explain how BAP31 modulates the expression of retina-related genes, we extracted 785 ER stress-related genes with a relevance score ≥ 7 from the GeneCards databases (Table S5). The DEGs identified through RNA sequencing exhibited an overlap of 134 common genes with those associated with ER stress (Figure 6A). The KEGG enrichment analysis of these candidate genes revealed that these DEGs were significantly enriched in protein processing in the ER, neurodegeneration, and apoptosis pathways (Figure 6B). We inferred that BAP31 plays a critical role in regulating protein homeostasis within the ER. However, the dysregulation of protein processing in ER may trigger the UPR, which may lead to the apoptosis of retinal photoreceptors.

To verify this mechanism, we examined the expression of ER stress-related genes in 12-month-old mice using qPCR and WB (Figure 6C,D). In the Rho-iCre-BAP31^fl/fl^(−/−) mice retina, there was a marked increase in the immunoglobulin heavy chain binding protein (*Bip*), *Xbp1*, and *Chop*, which are markers of ER stress, compared to BAP31^fl/fl^(+/+) retina. Bip is a well-known ER chaperone protein that plays an important role as a monitor of various protein modification processes. It has been reported that ER stress responses are activated as a protective mechanism to decrease cellular damage under various stress conditions. However, if the intensity of ER stress increases or persists over an extended period, cells may undergo ER stress overload. This phenomenon can ultimately lead to cell death [40,41]. We also detected the changes in expression of the marker genes of ER stress at various time points (Figure S6), and found that their expression significantly increased with advancing age. This finding further confirms that BAP31 deficiency triggered ER stress in an age-dependent manner, which subsequently led to secondary phototransduction dysfunction. These results suggest that the increase in ER stress observed in aged BAP31-deficient retinas may play an important role in promoting the apoptosis of photoreceptor cells. This indicates the critical role of ER stress pathways in the pathogenesis of retinal degeneration associated with BAP31 deficiency and provides further insight into the molecular mechanisms underlying photoreceptor cell loss.

Loss of BAP31 leads to impaired endoplasmic reticulum function, which in turn affects the folding of proteins. We used PROTEOSTAT, which is a dye used for detecting protein aggregation and the function of the protein quality control system to detect protein aggregates in the retina. It emitted a fluorescence signal by binding to protein aggregates. Terminal deoxynucleotidyl transferase dUTP nick end labeling (TUNEL) was used to detect the apoptosis of photoreceptor cells (Figure 6E). BAP31^fl/fl^(+/+) mice displayed fewer TUNEL- or PROTEOSTAT-positive cells in the outer nuclear layer at 12 month. In contrast, Rho-iCre-BAP31^fl/fl^(−/−) mice displayed TUNEL and distinct PROTEOSTAT staining in the outer nuclear layer at 12 months. The TUNEL results represent the retinal degeneration in Rho-iCre-BAP31^fl/fl^(−/−) mice. PROTEOSTAT staining was performed on TUNEL-detected mouse samples of the same age to evaluate the possible relationship between aggregation and photoreceptor cell apoptosis. The PROTEOSTAT staining is consistent with the TUNEL results, indicating a potential relationship between the misfolding of retina proteins and the apoptosis of photoreceptor cells, which was also consistent with previous results [42]. Some PROTEOSTAT staining was detected throughout the GCL of the mouse retinas and may represent non-specific staining, as the level of fluorescence was variable in different experiments. Notably, PROTEOSTAT staining also exhibited positive signals in INL of the retina in model mice. Based on the association between retinal cell structure and function, this can be attributed to secondary damage of bipolar cells induced by rod cell injury—a speculation supported by the results of ERG functional testing: under 3.0cd·s/m^2^ stimulation, the ERG b-wave of model mice was significantly reduced. However, the underlying regulatory mechanisms by which rod cell injury induces secondary damage to bipolar cells still require further verification.

Since BAP31 is a major endoplasmic reticulum function gene, we hypothesized that its absence leads to endoplasmic reticulum dysfunction, which in turn triggers the UPR response and causes endoplasmic reticulum stress. Under long-term stress, it further leads to the apoptosis of retinal photoreceptor cells and ultimately results in visual dysfunction. Our study findings suggest for the first time that BAP31 is associated with the pathological process of retinal degeneration involving the phototransduction signaling pathway.

## 4. Discussion

BAP31 is a transmembrane protein located in the ER that plays a significant role in various cellular processes, including protein trafficking, apoptosis, and ER stress responses. Given the current lack of direct evidence for the involvement of BAP31 in retinal degeneration, our study is the first to identify that retina-specific knockdown of BAP31 causes age-dependent retinal degenerative disease. However, the specific regulatory pathways still require further exploration, which also clarifies directions for future research. The significance of our study was that we used a BAP31 rod cell-specific knockdown mouse model, and systematically investigated the long-term effects of BAP31 deficiency on retinal neuronal development and visual function for the first time.

The OCT analysis revealed that the thickness of the ONL was significantly reduced in Rho-iCre-BAP31^fl/fl^(−/−) mice at 9 months of age, and this reduction became more severe at 12 months, which indicated progressive photoreceptor cell loss. This degenerative phenotype became more significant with advancing age, suggesting that the deterioration of retinal structure was time-dependent. ERG and OCT are standard ophthalmic tests used to assess retinal function and structure. Consistent with the results of the OCT test, the ERG results demonstrated a gradual decline in retinal function of BAP31 knockdown mice beginning at 9 months of age, with the functional impairment becoming increasingly severe over time. We used optomotor response testing to further evaluate the visual function. The results were in agreement with those of the OCT and ERG, showing that there were no significant differences in OMR between Rho-iCre-BAP31^fl/fl^(−/−) mice and BAP31^fl/fl^(+/+) mice prior to 6 months of age. However, a significant decrease in visual dynamic response indices was observed beginning at 9 months of age, with the disparity becoming increasingly evident by 12 months of age. Collectively, these findings demonstrate that retina-specific knockdown of BAP31 induces age-dependent retinal degeneration, characterized by progressive ONL thinning, photoreceptor loss, and visual function impairment. This proved that the lack of BAP31 in rod cell does not affect the early development of the retina but plays a critical role in maintaining retinal integrity during aging. The loss of BAP31 may cause the damaging retina response to chronic stress over a long time period, which may be due to the cumulative impact on cells, finally leading to the accelerated decline of visual function and the gradual deterioration of retina structures.

Transcriptomic profiling analysis of 12-month-old mice demonstrated that the specific ablation of BAP31 exerts a profound impact on the phototransduction pathway. The reduction in the expression of key phototransduction genes (*Rho*, *Rcvrn*, *Grk1*, *Gnat1*, *Gnb1*, *Gngt1*, *Pde6a*, *Pde6b*, *Pde6g*, *Gucy2e*, *Gucy2f*, *Guca1b*, *Slc24A1*, *Cngb1* and *Rgs9*) suggests a potential disruption in the phototransduction pathway, which could have significant implications for retinal function and vision [39,43]. The phototransduction pathway is a critical biochemical process by which photoreceptor cells in the retina (rods and cones) convert light into electrical signals. This pathway is essential for vision and involves a series of molecular events [44,45,46]. Reduced expression of any of these genes can disrupt this process, leading to decrease in sensitivity to light, delayed response to light stimuli, and impaired vision in low-light conditions (scotopic vision). This effect may be associated with photoreceptor loss, which leads to downregulated expression of phototransduction-related genes and consequently results in visual functional impairment in the animal model.

Another important finding in our study was the observation of upregulation in the expression of ER stress-associated marker genes, including *Bip, Chop, Xbp1, Atf6* and *Atf4*, which were the core components of the ER stress pathway. The high expression of Bip, a molecular chaperone, reflected the cell’s attempt to enhance protein folding capacity and prevent aggregation of misfolded proteins. Concurrently, *Chop* is a pro-apoptotic transcription factor, and *Atf4* is a regulator of stress-responsive genes; the increase in the two genes indicates a shift toward apoptotic signaling under prolonged ER stress conditions. Additionally, ATF6 and XBP1 are major transcription factors in the core pathway of the UPR; they both regulate protein homeostasis in cells in response to ER stress.

Collectively, these findings suggest that BAP31 may play a critical role in maintaining ER homeostasis. Our result confirms that BAP31 deficiency would impair the function of ER, leading to abnormalities in key processes such as protein folding and processing. Furthermore, ER dysfunction triggers ER stress. When this ER stress state accumulates over the long term, it ultimately induces the apoptosis of photoreceptor cells which involves the disorders of the transduction. The literature provides partial support for this mechanism; for example, 4-PBA treatment can effectively rescue the expression of phototransduction-related genes in EYS-RP cells [47]. However, simply blocking ER stress cannot fully prevent neuroinflammation-mediated loss of retinal RGC cells [48]. This indicates that retinal damage may be the result of the synergistic effect of multiple factors, rather than being caused by a single mechanism. Combined with existing research conclusions—that both 4-PBA and TUDCA exert their therapeutic effects in retinal degenerative diseases mainly by inhibiting ER stress, and further inhibiting apoptosis and neuroinflammation through this pathway—we infer that ER stress and apoptosis may have a synergistic effect, jointly promoting the progression of retinal damage.

Interestingly, no retinal degeneration or visual functional impairments were observed in younger animal models. Structural and functional deficits started at 9 months of age. These results were consistent with observations from previous studies, in which structural and functional defects were detected only at around 12–14 months of age after the deletion of XBP1 in the retina [49]. A similar phenotype was also observed in Atf6(−/−) mice, where the retinal morphology and function were normal in childhood, but developed rod and cone dysfunction with increasing age until 18 months [50]. These results suggest that BAP31, XBP1, and ATF6 deficiencies exhibit delayed-onset phenotypes, with retinal degeneration and functional decline becoming apparent only at advanced ages. This late-onset phenotype is also observed in IRE1 knockout model mice [12]. This phenomenon is highly consistent with the previous research results of our team in the Alzheimer’s disease model [22]. In this model, animals only showed obvious symptoms after reaching 10 months of age, and this result indicates that the simple knockout of BAP31 is not sufficient to induce pathological changes in the early stage of the disease. Although mice with ATF6, XBP1, or IRE1 gene knockout all display delayed-onset retinal phenotypes, their disease onset timelines differ significantly from our model. Specifically, the ATF6 knockout model manifests pathological changes at 18 months of age, while the XBP1 knockout model exhibits disease onset between 12 and 14 months of age. Both models exhibit later onset compared to our BAP31 knockout model (onset at 9 months of age), thus classifying them as late-stage animal models. In contrast, although the IRE1 knockout model is also categorized as delayed-onset, its disease onset occurs earlier at 6 months of age, preceding our model. Notably, the BAP31 knockdown model in our study initiates phenotypic changes at 9–12 months of age, which precisely fills the gap in the 9–12 months age-dependent onset window among ER stress-related models. This characteristic aligns more closely with the clinical onset pattern of human age-related retinal degenerative diseases, which typically commence in the late middle-aged period. Furthermore, ATF6, XBP1, and IRE1 are direct effectors of the ER stress pathway; their mutation or knockout directly disrupts key components of this pathway. In contrast, BAP31 functions as an indirect regulator localized on the ER membrane, modulating ER stress pathway homeostasis by controlling ER-associated protein transport. This mechanistic distinction highlights that our model is uniquely suited for investigating retinal degeneration induced by “non-direct pathway disruption”, thereby offering a novel perspective to decipher the diverse mechanisms underlying ER stress-mediated diseases. Collectively, the successful establishment of this BAP31 knockdown model not only advances the research framework of ER stress-related animal models but also enriches the theoretical basis for understanding how ER stress regulates retinal degenerative diseases. Previous studies have found that the fidelity of protein quality control and proteostasis regulatory mechanisms was deteriorated with advancing age in *Caenorhabditis elegans*, resulting in elevated ER stress levels and the accumulation of misfolded proteins [51,52]. The results of our study further confirm the critical role of BAP31 in the regulation of protein processing and folding in retinal cells. The retina cells were highly susceptible to disruptions in proteostasis due to their high metabolic demands and specialized functions; however, dysfunction of BAP31 may impair ER function, causing abnormalities in key processes such as protein folding and processing, ultimately triggering ER stress. It is important to note that BAP31 is not a direct trigger of ER stress; instead, BAP31 acts as a regulator of ER function. This differs from models such as those induced by tunicamycin, rhodopsin misfolding, or Tulp1 mutants, pathogenic factors that are direct triggers of ER stress. Thus, the deficiency of BAP31 may not trigger acute ER stress immediately; rather, functional defects gradually manifest over time. Additionally, during retinal development, a compensatory mechanism may exist in the context of BAP31 deficiency, which can temporarily offset its functional defects. For example, the compensation may occur through the activation of the IRE1 and ATF6 pathways. The IRE1 could enhance the clearance efficiency of abnormal proteins by upregulating the expression of ER-associated degradation (ERAD)-related proteins [2]; after translocating to the nucleus, ATF6 promotes the transcription of ER chaperone proteins, thereby enhancing the ability of ER to fold unfolded proteins [53]. This compensatory mechanism is sufficient to maintain normal retinal structure and function in young animals. Only when animals age and their compensatory capacity gradually declines does retinal degeneration begin to manifest. Specifically, dysfunction of the ER may first trigger ER stress; when this stress state accumulates over the long term and exceeds the regulatory capacity of other compensatory mechanisms during development, it will eventually induce apoptosis in the photoreceptor. This process is accompanied by impairments in the phototransduction pathway, which in turn leads to visual dysfunction in animal models. Since this pathological process is associated with animal development and aging, it ultimately presents as a delayed-onset phenotype. Our results offer mechanistic insight into retinal degenerative diseases such as retinitis pigmentosa and age-related macular degeneration with the potential contribution of BAP31 dysfunction.

Consequently, our findings suggest that BAP31 is either non-essential or its deficiency can be compensated for under conditions of photoreceptor homeostasis or normal ER stress. However, BAP31 assumes increasing significance during aging when cells are subjected to heightened ER stress, emphasizing its critical role in the regulation of ER mechanisms in aged animals. It may potentially implicate shared mechanisms related to age-dependent stress responses or protein homeostasis dysregulation. While our study has shed light on the transcriptional changes in the phototransduction pathway and the ER stress pathway associated with BAP31 deficiency, the intrinsic mechanisms linking these pathways remains to be fully elucidated. Combining published research and our experimental findings, we hypothesize that the ER dysfunction caused by BAP31 deficiency may exert a synergistic effect with aging pathways. Specifically, aging accelerates ER stress-induced cellular damage, while ER stress further exacerbates the aging-related disruption of protein homeostasis; these two factors collectively drive the manifestation of phenotypes in mice aged 9–12 months. Thus, the BAP31 knockdown model serves as an ideal experimental model for studying age-related retinal neurodegenerative diseases.

Another significant feature of this study is that it provides a new animal model for investigating the pathogenesis of age-related retinal diseases. Experimental models have played a crucial role in elucidating genetic and pharmacological mechanisms, particularly in the study of familial neurodegenerative diseases. However, whether these models were suitable for explaining the etiology of age-related neurodegenerative diseases remained questionable. As is widely known, rd1 and rd10 are mouse models established for the study of retinitis pigmentosa. Both models arise from the mutations in the Pde6b gene, which trigger apoptosis of rod photoreceptors and ultimately lead to RP [54]. However, these two models have significant limitations regarding age: rd1 exhibits an exceptionally rapid disease progression, with severe outer retinal degeneration evident as early as postnatal day 14. In contrast, at this same developmental stage, most rd10 mice display normal spectral-domain optical coherence tomography (SD-OCT) images, with only a subset showing subtle structural alterations at the inner segment/outer segment (IS/OS) junction, reflecting a comparatively slower disease course. Notably, the peak of rod photoreceptor loss in rd10 mice occurs around three weeks of age; the onset rate of the disease is also relatively fast [55]. Given the established age correspondence between mice and humans [56], the early onset of retinal degeneration in both models limits their suitability as representative systems for studying age-related forms of RP. In contrast, the BAP31 knockdown model developed in our study offers significant advantages. Retinal degeneration in this model begins at approximately 9 months of age (equivalent to 38–47 years in human age), which aligns closely with the characteristics of age-dependent disease onset. More importantly, therapeutic interventions in rd1 and rd10 models have primarily targeted correction of phototransduction defects, while our model highlights pathways involving regulation of endoplasmic reticulum homeostasis and enhancement of cellular compensatory mechanisms. These findings suggest a promising therapeutic avenue for late-onset RP and provide critical experimental support for the development of age-adapted treatment strategies tailored to elderly patients. In previous studies, through the overexpression of mutant proteins or the use of neurotoxins, certain pathological features of diseases could be induced in animals. However, the pathogenesis of these diseases may be different from those of age-related diseases. These experimental models have certain limitations. For instance, overexpression of mutant proteins could lead to abnormal protein aggregation and cellular stress, but this may not be fully consistent with the gradual dysregulation of protein homeostasis observed in sporadic diseases. Neurotoxins (such as MPTP and 6-OHDA) could rapidly induce pathological changes, but these phenotypes were different from the chronic progression of age-related diseases. Additionally, most experimental models used were young animals, which cannot fully replicate the cellular and molecular changes that occur during the aging process. Therefore, whether the mechanisms revealed in these models are relevant to the etiology of the majority of age-related neurodegenerative diseases remains an unresolved question. The retinal degenerative disease model induced by the specific deletion of BAP31 in our study exhibits characteristics of age-related diseases and mimics the pathogenesis of this disease. Thus, it serves as an ideal experimental model for studying age-related retinal neurodegenerative diseases.

Furthermore, recent studies have also confirmed that a programmed cell death mechanism independent of caspases—PARP1-dependent programmed cell death (parthanatos)—that plays a key role in the rd10 mouse model [57]. Moreover, this mechanism may be independent of disease-causing mutant genes and directly involved in the death process of photoreceptor cells in RP. The core mechanism of parthanatos is that DNA damage signals trigger the recruitment and activation of poly (ADP-ribose) polymerase 1 (PARP1) in the nucleus. The abnormal activation of PARP1 has been clearly confirmed to be a key driver of retinal degeneration [58].

Based on the findings of this study, BAP31 deficiency disrupts the membrane protein transport function of the ER, triggering persistent ER stress. We hypothesize that this chronic ER stress may also induce DNA damage, which in turn activates parthanatos. To verify this hypothesis and clarify the regulatory role of parthanatos, we will use the PARP1-specific inhibitor Olaparib to intervene in BAP31-deficient model mice in future studies. By detecting the thickness of the ONL, visual function (ERG/OMR), and cell death ratio, we will evaluate the alleviating effect of parthanatos inhibition on the retinal degeneration phenotype induced by BAP31 deficiency. Meanwhile, considering that excessive activation of PARP1 consumes a large amount of intracellular NAD+, leading to energy metabolism disorders and further aggravating cell death, we also plan to use NAD+ precursor substances (e.g., β-nicotinamide mononucleotide, NMN) for intervention in subsequent studies. By detecting the NAD+ content in retinal tissue, ER stress markers (e.g., GRP78, PERK phosphorylation), and parthanatos-related indicators (e.g., PAR polymer levels), we will observe whether NAD+ supplementation can synergistically improve ER stress and parthanatos-mediated cell death, providing a potential target reference for the clinical development of targeted intervention strategies.

## 5. Conclusions

The findings indicate that BAP31, as an ER-stress-regulatory molecule, plays a critical role in age-dependent retinal degenerative diseases. We found that BAP31 deficiency downregulates the expression of key genes in the phototransduction pathway, blocking the conversion of light signals to electrical signals; it also impairs ER homeostasis, leading to increased expression of ER stress markers. It is important to emphasize that the occurrence of retinal degenerative lesions is not driven by a single pathway but rather synergistically driven by multiple factors, such as chronic ER stress, aging, and apoptosis. This also explains the age-dependent characteristic of lesions induced by BAP31 deficiency. Here, we provide the first evidence that BAP31 is involved in regulating age-dependent retinal degenerative lesions. The BAP31 knockdown model constructed in this study highly aligns with the pathogenesis of human age-related diseases and highlights the core significance of ER homeostasis regulation in lesions. It provides an ideal experimental model for mechanism research and intervention exploration of late-onset retinitis pigmentosa.

## Data Availability

The datasets presented in this study can be found in online repositories. The names of the repository/repositories and accession number(s) can be found below: https://ngdc.cncb.ac.cn/gsa/search?searchTerm=CRA031022 (accessed on 2 October 2025). The data are available in the article itself and its Supplementary Materials.

## Supplementary Materials

The following supporting information can be downloaded at https://www.mdpi.com/article/10.3390/cells14221802/s1, Table S1: Primer sequences; Table S2: Peripheral point data of outer nuclear layer (ONL) thickness; Table S3: The 338 retinal disease-associated genes retrieved from RetNet database; Table S4: The 418 retina-related genes (with a relevance score ≥ 7) extracted from the GeneCards database; Table S5: ER stress-related genes with a relevance score of not less than 7 (total number of genes, 785). Figure S1: Deletion of BAP31 in Mouse Rod Photoreceptors via Rho-iCre; Figure S2: BAP31 specific knockdown results in Age-Related Degeneration; Figure S3: Electroretinography (ERG) recording analysis under 0.15 cd·s/m^2^ and 10 cd·s/m^2^; Figure S4: Electroretinography (ERG) recording analysis; Figure S5: Heatmap of gene expression in the phototransduction pathway; Figure S6: BAP31 regulated UPR to affect the expression of phototransduction genes.

## Abbreviations

The following abbreviations are used in this manuscript:

RD	Retinal degeneration
AMD	Age-related macular degeneration
RP	Retinitis pigmentosa
DR	Diabetic retinopathy
IRD	Inherited retinal degeneration
ER	Endoplasmic reticulum
BAP31	B cell receptor-associated protein 31
UPR	Unfolded protein response
ERG	Electroretinogram
OCT	Optical Coherence Tomography
OMR	Optomotor response
qPCR	Quantitative real-time polymerase chain reaction
HE	Hematoxylin–eosin
ONL	Outer nuclear layer
DEGs	Differentially expressed genes
