# Supplementary material for: Loss of BAP31 Is Detrimentally Aging Photoreceptors Through ER Stress-Mediated Retinal Degeneration
Source: Cells. 2025 Nov 17;14(22):1802. doi: 10.3390/cells14221802 (PMC12650883; doi:10.3390/cells14221802)
Supplement: Supplementary file 1 [file cells-14-01802-s001.zip › Supplement-Table.pdf]

TableS1. The sequence of primer

Gene	Forward primer	Reverse primer
BAP31	CTGCTGTCCTTCCTGC TTAG	CTCCTGTTCTCTTCCTC CAAC
Gnat1	GATGCCCGCACTGTGAAAC	CCAGCGAATACCCGTCCTG
Pde6b	GCAGCACTTTTTGAACTGGTG	CATTGCGCTGGCGGTACATA
Pde6a	CAGCAACTACCACGATGTGAA	GTGGACATTGAAGAGCCTAGTG
Rho	CCCTTCTCCAACGTCACAGG	GTAGAGCGTGAGGAAGTTGATG
Rcvrn	ACGACGTAGACGGCAATGG	CCGCTTTTCTGGGGTGTTTT
NRL	CTGCCCGAGAGATTTTCGGAC	CTTGGAGCGACAAGCCTGAG
GFAP	ACCAGCTTACGGCCAACAG	CCAGCGATTCAACCTTTCTCT
Gngt1	TCAACATCGAAGACCTGACAGA	ACACAGCCTCCTTTGAGTTCC
Rom1	CTCCAACCCCGTATCCGTTTG	GAGCAGGGAATGAACAAGAGG
Crx	GTTCCTATTTCAGTGGGTTGGAT	CCACAGGACTGTAGGTGCTAT
Bip	GCATCACGCCGTCGTATGT	ATTCCAAGTGCGTCCGATGAG
Chop	CTCGCTCTCCAGATTCCAGTC	CTTCATGCGTTGCTTCCCA
Xbp1	AGCTTTTACGGGAGAAAACTCAC	CCTCTGGAACCTCGTCAGGA
ATF4	CTCTTGACCACGTTGGATGAC	CAACTTCACTGCCTAGCTCTAAA
ATF6	TCGCCTTTTAGTCCGGTTCTT	GGCTCCATAGGTCTGACTCC
GAPDH	TGGCCTTCCGTGTTCCTAC	GAGTTGCTGTTGAAGTCGCA

TableS2. Peripheral point data of outer nuclear layer (ONL) thickness

Month	Distances(μm)	-1200	-900	-600	-300	300	600	900	1200
3M	BAP31$^{fl/fl}$(+/+)	60.6149	64.0302	59.1731	39.3535	41.0834	57.5228	57.1953	55.3137
	Rho-icre-BAP31$^{fl/fl}$(-/-)	60.4118	61.4619	56.7671	35.6951	37.3404	55.2495	57.6431	55.6439
	P	ns	ns	ns	ns	ns	ns	ns	ns
6M	BAP31$^{fl/fl}$(+/+)	56.9659	53.5697	51.7857	39.8205	39.5102	51.1409	53.6058	48.796
	Rho-icre-BAP31$^{fl/fl}$(-/-)	51.6262	52.6805	52.8063	36.3634	38.434	48.6652	48.5681	47.3961
	P	ns	ns	ns	ns	ns	ns	ns	ns
9M	BAP31$^{fl/fl}$(+/+)	56.2489	55.7099	51.3098	34.8135	34.2372	51.2347	53.0768	51.1075
	Rho-icre-BAP31$^{fl/fl}$(-/-)	48.0993	48.3169	46.8953	30.976	32.0255	43.9473	42.4869	42.325
	P	*	**	*	*	*	*	**	*
12M	BAP31$^{fl/fl}$(+/+)	56.0025	53.9943	49.1319	33.0678	31.6967	47.6883	50.5396	50.3238
	Rho-icre-BAP31$^{fl/fl}$(-/-)	41.5752	38.3075	37.1876	25.0465	24.9097	30.4363	25.776	26.1272
	P	*	***	**	*	*	***	***	***

TableS3.The 338 retinal disease-associated genes retrieved from RetNet database

Disease Category	Identified Genes
Bardet-Biedl syndrome, autosomal recessive	ADIPOR1, ARL6, BBIP1, BBS1, BBS10, BBS12, BBS2, BBS4, BBS5, BBS7, BBS9, CEP19, CEP290CFAP418, IFT172, IFT27, IFT74, INPP5E, LZTFL1, MKKS, MKS1, NPHP1, SCAPER, SDCCAG8, TRIM32, TTC8
Chorioretinal atrophy or degeneration, autosomal dominant	PRDM13, RGR, TEAD1
Cone or cone-rod dystrophy, autosomal dominant	AIPL1, CRX, GUCA1A, GUCY2D, PITPNM3, PROM1, PRPH2, RIMS1, SEMA4A, THRB, UNC119
Cone or cone-rod dystrophy, autosomal recessive	ABCA4, ADAM9, ATF6, C19Orf44, CACNA2D4, CDHR1, CEP78, CERKL, CFAP410, CFAP418, CNGA3, CNGB3, CNNM4, DYNC2I2, GNAT2, IFT81, KCNV2, PDE6C, PDE6H, POC1B, RAB2, RAX2, RDH5, RPGRIP1, SLC4A7, TLCD3B, TTLL5, UBAP1L
Cone or cone-rod dystrophy, X-linked	CACNA1F, OPSIN-LCR, RPGR
Congenital stationary night blindness, autosomal	GNAT1,PDE6B, RHO

dominant	
Congenital stationary night blindness, autosomal recessive	CABP4, GNAT1, GNB3, GPR179, GRK1, GRM6, LRIT3, RDH5, SAG, SLC24A1, TRPM1
Congenital stationary night blindness, X-linked	CACNA1F, NYX
Deafness alone or syndromic, autosomal dominant	ESPN, WFS1
Deafness alone or syndromic, autosomal recessive	CDH23, CIB2, ESPN, MYO7A, PCDH15, PDZD7, USH1C, WHRN
Leber congenital amaurosis, autosomal dominant	CRX,IMPDH1,OTX2,TUBB4B
Leber congenital amaurosis, autosomal recessive	AIPL1, CABP4, CCT2, CEP290, CLUAP1, CRB1, CRX, DTHD1, GDF6, GUCY2D IDH3A, IFT140, IQCB1, KCNJ13, LCA5, LRAT, NMNAT1, PRPH2, RD3, RDH12, RPE65, RPGRIP1, SPATA7.<br>TULP1, USP45
Macular degeneration, autosomal dominant	BEST1, C1QTNF5, CLEC3B, CTNNA1, EFEMP1, ELOVL4, FSCN2, GUCA1B, HMCN1, IMPG1LRRTM4, OTX2, PRDM13, PROM1, PRPH2, RP1L1, THRB, TIMP3
Macular degeneration, autosomal recessive	UBAP1L, WDR19, ABCA4, AP5B1, AP5M1, AP5Z1, CFH, DRAM2, IMPG1, MFSD8, MPDZ, SAMD7, SLC37A3
Macular degeneration, X-linked	RPGR
Ocular-retinal developmental disease, autosomal dominant	VCAN
Optic atrophy, autosomal dominant	AFG3L2, DNM1L, MFN2, MIEF1, NR2F1, OPA1, SPG7
Optic atrophy, autosomal recessive	ACO2, NBAS, RTN4IP1, TMEM126A
Optic atrophy, X-linked	TIMM8A
Retinitis pigmentosa, autosomal dominant	ADIPOR1, ARL3, BEST1, CRX, FSCN2, GUCA1B, HK1, IMPDH1, IMPG1, KIF3B, KLHL7, NR2E3NRL, PRPF3, PRPF31, PRPF4, PRPF6, PRPF8, PRPH2, RDH12, RHO, ROM1, RP1, RP17, RP9.RPE65, SAG, SEMA4A, SNRNP200, SPP2, TOPORS, TUBB4B, VWA8
Retinitis pigmentosa, autosomal recessive	ABCA4, ADGRA3, AGBL5, AHR, ARHGEF18, ARL2BP ARL6, BBS1, BBS2, BEST1, CC2D2ACCDC51, CEP162, CERKL, CFAP20, CFAP410, CFAP418, CLCC1, CLRN1, CNGA1, CNGB1, COQ2, COQ4, COQ5, COQ8B, CRB1, CRB2, CWC27, CYP4V2, DHDDS, DHX38, EMC1, ENSA, EYS, FAM161A, HGSNAT, HKDC1, IDH3A, IDH3B, IFT140, IFT172, IFT43, IMPG2, KIAA1549KIZ, LRAT, MAK, MERTK, MVK, NEK2, NEUROD1, NR2E3, NRL, PCARE, PDE6A,

	PDE6B, PDE6G, PDSS1, POMGNT1, PRCD, PROM1, PROS1, RAX2, RBP3, REEP6, RGR, RHO, RLBP1RP1, RP1L1, RPE65, SAG, SAMD11, SCAPER, SLC37A3, SLC39A12, SLC66A1, SLC7A14, SPATA7, STX3, SUMF1, TBC1D32, TMEM216, TRNT1, TTC8, TULP1, UBAP1L, USH2A, ZNF408, ZNF513
Retinitis pigmentosa, X-linked	IDH3G, OFD1, RP2, RPGR
Syndromic/systemic diseases with retinopathy, autosomal dominant	ABCC6, AFG3L2, AGBL5, ATXN7, COL11A1, COL2A1, JAG1, KCNJ13, KIF11, MFN2NOTCH2NLC, NR6A1, NTN1, OPA3, PAX2, TREX1, VCAN
Syndromic/systemic diseases with retinopathy, autosomal recessive	ABCC6, ABHD12, ACBD5, ACO2, ADAMTS18, ADIPOR1, AFG3L2, AGPAT3, AHI1, ALMS1ARL13B, ARMC9, ATIC, CC2D2A, CEP164, CEP290, CISD2, CLCN2, CLN3, CLN5, COL9A1,CSPP1, CWC27, DYNC2I2, ELOVL4, EXOSC2, FDXR, FLVCR1, GNPTG, HARS, HGSNAT, HMX1IFT140, IFT81, INPP5E, INVS, IOCB1, LAMA1, LRP5, MKS1, MTTP NPHP1, NPHP3, NPHP4OPA3, PANK2, PCYT1A, PDSS1, PEX1, PEX2, PEX7, PHYH, PLK4, PNPLA6, POC1B, POC5, PPT1, PRPS1, RDH11, RIMS2, RPGRIP1L, SCAPER, SDCCAG8, SLC25A46, SLC38A8, SPG7STX3, TMEM216, TMEM237, TMEM67, TRNT1, TTPA, TUB, TUBGCP4, TUBGCP6, WDPCPWDR19, WFS1, ZNF423
Syndromic/systemic diseases with retinopathy, X-linked	LAMP2, OFD1,TIMM8A
Usher syndrome, autosomal recessive	PCDH15, USH1C, USH1G, USH2A, WHRN, ABHD12, ADGRV1, ARSG, CDH23, CEP250, CEP78, CIB2, CLRN1, ESPN, HARS, MYO7A
Other retinopathy, autosomal dominant	BEST1, CAPN5, CRB1, CTNNB1, ELOVL1, FZD4, ITM2B, KIF3B, LRP5, MAPKAPK3, MIR204MMP19, OPN1SW, RB1, RCBTB1, RGR, TSPAN12, ZNF408
Other retinopathy, autosomal recessive	ASRGL1, ATOH7, BEST1, C120rf65, CDH3, CNGA3, CNGB3, CNNM4, COQ2, CYP4V2DYNC2H1, LRP5, MFRP MVK, NBAS, NDUFAF5, NR2E3, OAT, PLA2G5, PROM1, RBP4RCBTB1, RGS9, RGS9BP RLBP1
Other retinopathy, mitochondrial	KSS, LHON, MT-ATP6, MT-TH, MT-TL1, MT-TP MT-TS2
Other retinopathy, X-linked	CACNA1F, CHM, DMD, NDP OPN1LW, OPN1MW, PGK1, RS1

TableS4.The 418 retina-related genes (with a relevance score ≥7) extracted from the GeneCards database

Gene Symbol	Description	Category	Uniprot ID	Gifts	GC Id	Relevance score	GeneCards Link
OAT	Ornithine Aminotransferase	Protein Coding	P04181	60	GC10M124397	64.6324691772461	https://www.genecards.org/cgi-bin/carddisp.pl?gene=OAT
ABCA4	ATP Binding Cassette Subfamily A Member 4	Protein Coding	P78363	57	GC01M093992	36.5983047485352	https://www.genecards.org/cgi-bin/carddisp.pl?gene=ABCA4
PAX6	Paired Box 6	Protein Coding	P26367	60	GC11M031784	34.7335395812988	https://www.genecards.org/cgi-bin/carddisp.pl?gene=PAX6
NRL	Neural Retina Leucine Zipper	Protein Coding	P54845	51	GC14M024078	33.2439727783203	https://www.genecards.org/cgi-bin/carddisp.pl?gene=NRL
RHO	Rhodopsin	Protein Coding	P08100	59	GC03P144186	31.9091243743896	https://www.genecards.org/cgi-bin/carddisp.pl?gene=RHO
PRPH2	Peripherin 2	Protein Coding	P23942	52	GC06M111790	30.8561763763428	https://www.genecards.org/cgi-bin/carddisp.pl?gene=PRPH2
GUCY2D	Guanylate Cyclase 2D, Retinal	Protein Coding	Q02846	58	GC17P008002	30.3278827667236	https://www.genecards.org/cgi-bin/carddisp.pl?gene=GUCY2D

RPE65	Retinoid Isomerohydrolase RPE65	Protein Coding	Q16518	57	GC01M068428	29.4226131439209	https://www.genecards.org/cgi-bin/carddisp.pl?gene=RPE65
RAX2	Retina And Anterior Neural Fold Homeobox 2	Protein Coding	Q96IS3	44	GC19M003769	28.844690322876	https://www.genecards.org/cgi-bin/carddisp.pl?gene=RAX2
USH2A	Usherin	Protein Coding	O75445	50	GC01M215622	28.2408771514893	https://www.genecards.org/cgi-bin/carddisp.pl?gene=USH2A
SAG	S-Antigen Visual Arrestin	Protein Coding	P10523	57	GC02P235463	27.4352321624756	https://www.genecards.org/cgi-bin/carddisp.pl?gene=SAG
NR2E3	Nuclear Receptor Subfamily 2 Group E Member 3	Protein Coding	Q9Y5X4	55	GC15P071792	27.0443706512451	https://www.genecards.org/cgi-bin/carddisp.pl?gene=NR2E3
CRX	Cone-Rod Homeobox	Protein Coding	O43186	53	GC19P047819	26.8643074035645	https://www.genecards.org/cgi-bin/carddisp.pl?gene=CRX
LOC121815974	NANOG HESC Enhancer GRCh37_chr10:126092029-126092572	Functional Element		7	GC10P124403	26.5827827453613	https://www.genecards.org/cgi-bin/carddisp.pl?gene=LOC121815974
RS1	Retinoschisin 1	Protein Coding	O15537	52	GC0XM018639	26.436450958252	https://www.genecards.org/cgi-bin/carddisp.pl?gene=RS1
CRB1	Crumbs Cell Polarity Complex Component 1	Protein Coding	P82279	55	GC01P1972	24.9798278808594	https://www.genecards.org/cgi-bin/carddisp.pl?gene=CRB1

					08		
BEST1	Bestrophin 1	Protein Coding	O76090	56	GC11 P0619 49	24.541698 4558105	https://www.genecards.org/cgi-bin/car ddisp.pl?gene=BEST1
RAX	Retina And Anterior Neural Fold Homeobox	Protein Coding	Q9Y2V3	45	GC18 M059 267	24.281169 8913574	https://www.genecards.org/cgi-bin/car ddisp.pl?gene=RAX
AIPL1	AIP Like 1 HSP90 Co-Chaperone	Protein Coding	Q9NZN9	53	GC17 M006 393	23.964017 868042	https://www.genecards.org/cgi-bin/car ddisp.pl?gene=AIPL1
RPGR	Retinitis Pigmentosa GTPase Regulator	Protein Coding	Q92834	53	GC0X M038 269	23.751396 1791992	https://www.genecards.org/cgi-bin/car ddisp.pl?gene=RPGR
PLA2G5	Phospholipase A2 Group V	Protein Coding	P39877	51	GC01 P0200 28	23.449544 9066162	https://www.genecards.org/cgi-bin/car ddisp.pl?gene=PLA2G5
GUCA1A	Guanylate Cyclase Activator 1A	Protein Coding	P43080	52	GC06 P1908 72	22.934154 510498	https://www.genecards.org/cgi-bin/car ddisp.pl?gene=GUCA1A
TULP1	TUB Like Protein 1	Protein Coding	O00294	53	GC06 M111 703	22.554342 2698975	https://www.genecards.org/cgi-bin/car ddisp.pl?gene=TULP1
RDH12	Retinol Dehydrogenase 12	Protein Coding	Q96NR8	56	GC14 P0677 01	22.470733 6425781	https://www.genecards.org/cgi-bin/car ddisp.pl?gene=RDH12
NDP	Norrin Cystine Knot Growth Factor	Protein	Q00604	54	GC0X	22.458366	https://www.genecards.org/cgi-bin/car

	NDP	Coding			M043 948	394043	ddisp.pl?gene=NDP
RLBP1	Retinaldehyde Binding Protein 1	Protein Coding	P12271	57	GC15 M089 209	22.452384 9487305	https://www.genecards.org/cgi-bin/carddisp.pl?gene=RLBP1
CNGB3	Cyclic Nucleotide Gated Channel Subunit Beta 3	Protein Coding	Q9NQW 8	53	GC08 M086 553	21.940124 5117188	https://www.genecards.org/cgi-bin/carddisp.pl?gene=CNGB3
RBP3	Retinol Binding Protein 3	Protein Coding	P10745	54	GC10 P0473 48	21.936454 7729492	https://www.genecards.org/cgi-bin/carddisp.pl?gene=RBP3
PROM1	Prominin 1	Protein Coding	O43490	60	GC04 M015 965	21.836730 9570313	https://www.genecards.org/cgi-bin/carddisp.pl?gene=PROM1
RIMS1	Regulating Synaptic Membrane Exocytosis 1	Protein Coding	Q86UR5	55	GC06 P0718 86	21.828409 1949463	https://www.genecards.org/cgi-bin/carddisp.pl?gene=RIMS1
CNGA3	Cyclic Nucleotide Gated Channel Subunit Alpha 3	Protein Coding	Q16281	55	GC02 P1009 73	21.738039 0167236	https://www.genecards.org/cgi-bin/carddisp.pl?gene=CNGA3
MYO7A	Myosin VIIA	Protein Coding	Q13402	56	GC11 P0771 28	21.386224 7467041	https://www.genecards.org/cgi-bin/carddisp.pl?gene=MYO7A
PNPLA6	Patatin Like Domain 6, Lysophospholipase	Protein Coding	Q8IY17	56	GC19 P0075 34	21.259983 0627441	https://www.genecards.org/cgi-bin/carddisp.pl?gene=PNPLA6

RPGRIP1	RPGR Interacting Protein 1	Protein Coding	Q96KN7	52	GC14 P0602 88	21.170269 0124512	https://www.genecards.org/cgi-bin/carddisp.pl?gene=RPGRIP1
GUCA1B	Guanylate Cyclase Activator 1B	Protein Coding	Q9UMX6	49	GC06 M111 789	20.997039 7949219	https://www.genecards.org/cgi-bin/carddisp.pl?gene=GUCA1B
FZD5	Frizzled Class Receptor 5	Protein Coding	Q13467	58	GC02 M207 762	20.797277 4505615	https://www.genecards.org/cgi-bin/carddisp.pl?gene=FZD5
EYS	Eyes Shut Homolog	Protein Coding	Q5T1H1	45	GC06 M063 719	20.643522 2625732	https://www.genecards.org/cgi-bin/carddisp.pl?gene=EYS
RDH5	Retinol Dehydrogenase 5	Protein Coding	Q92781	57	GC12 P0557 20	20.400800 7049561	https://www.genecards.org/cgi-bin/carddisp.pl?gene=RDH5
CEP290	Centrosomal Protein 290	Protein Coding	O15078	53	GC12 M088 049	20.348304 7485352	https://www.genecards.org/cgi-bin/carddisp.pl?gene=CEP290
CACNA1F	Calcium Voltage-Gated Channel Subunit Alpha1 F	Protein Coding	O60840	56	GC0X M049 205	20.061672 2106934	https://www.genecards.org/cgi-bin/carddisp.pl?gene=CACNA1F
PDE6B	Phosphodiesterase 6B	Protein Coding	P35913	57	GC04 P0315 56	19.742261 8865967	https://www.genecards.org/cgi-bin/carddisp.pl?gene=PDE6B
SPATA7	Spermatogenesis Associated 7	Protein Coding	Q9P0W8	48	GC14 P0883	19.358715 057373	https://www.genecards.org/cgi-bin/carddisp.pl?gene=SPATA7

					84		
LRAT	Lecithin Retinol Acyltransferase	Protein Coding	O95237	56	GC04 P1546 26	19.261293 4112549	https://www.genecards.org/cgi-bin/carddisp.pl?gene=LRAT
IMPG1	Interphotoreceptor Matrix Proteoglycan 1	Protein Coding	Q17R60	50	GC06 M075 921	18.878431 3201904	https://www.genecards.org/cgi-bin/carddisp.pl?gene=IMPG1
VSX2	Visual System Homeobox 2	Protein Coding	P58304	52	GC14 P0742 39	18.733373 6419678	https://www.genecards.org/cgi-bin/carddisp.pl?gene=VSX2
BBS1	Bardet-Biedl Syndrome 1	Protein Coding	Q8NFJ9	52	GC11 P1151 39	18.368021 0113525	https://www.genecards.org/cgi-bin/carddisp.pl?gene=BBS1
CHM	CHM Rab Escort Protein	Protein Coding	P24386	52	GC0X M085 861	18.288387 298584	https://www.genecards.org/cgi-bin/carddisp.pl?gene=CHM
IMPDH1	Inosine Monophosphate Dehydrogenase 1	Protein Coding	P20839	60	GC07 M128 392	17.914291 3818359	https://www.genecards.org/cgi-bin/carddisp.pl?gene=IMPDH1
CFH	Complement Factor H	Protein Coding	P08603	59	GC01 P1966 21	17.827024 4598389	https://www.genecards.org/cgi-bin/carddisp.pl?gene=CFH
EFEMP1	EGF Containing Fibulin Extracellular Matrix Protein 1	Protein Coding	Q12805	56	GC02 M055 865	17.542915 3442383	https://www.genecards.org/cgi-bin/carddisp.pl?gene=EFEMP1
GNAT2	G Protein Subunit Alpha	Protein	P19087	56	GC01	17.474399	https://www.genecards.org/cgi-bin/car

	Transducin 2	Coding			M109 603	5666504	ddisp.pl?gene=GNAT2
LRP5	LDL Receptor Related Protein 5	Protein Coding	O75197	62	GC11 P0682 98	17.425756 4544678	https://www.genecards.org/cgi-bin/carddisp.pl?gene=LRP5
ELOVL4	ELOVL Fatty Acid Elongase 4	Protein Coding	Q9GZR5	57	GC06 M079 914	17.377431 8695068	https://www.genecards.org/cgi-bin/carddisp.pl?gene=ELOVL4
FZD4	Frizzled Class Receptor 4	Protein Coding	Q9ULV1	62	GC11 M086 945	17.212869 644165	https://www.genecards.org/cgi-bin/carddisp.pl?gene=FZD4
IMPG2	Interphotoreceptor Matrix Proteoglycan 2	Protein Coding	Q9BZV3	50	GC03 M101 222	17.148843 7652588	https://www.genecards.org/cgi-bin/carddisp.pl?gene=IMPG2
ROM1	Retinal Outer Segment Membrane Protein 1	Protein Coding	Q03395	55	GC11 P0626 11	17.146396 6369629	https://www.genecards.org/cgi-bin/carddisp.pl?gene=ROM1
ZNF408	Zinc Finger Protein 408	Protein Coding	Q9H9D4	45	GC11 P0467 01	17.073570 2514648	https://www.genecards.org/cgi-bin/carddisp.pl?gene=ZNF408
OTX2	Orthodenticle Homeobox 2	Protein Coding	P32243	59	GC14 M056 799	17.072364 8071289	https://www.genecards.org/cgi-bin/carddisp.pl?gene=OTX2
RP1	RP1 Axonemal Microtubule Associated	Protein Coding	P56715	49	GC08 P0547 07	16.977201 461792	https://www.genecards.org/cgi-bin/carddisp.pl?gene=RP1

RD3	RD3 Regulator Of GUCY2D	Protein Coding	Q7Z3Z2	47	GC01 M211 476	16.934591 293335	https://www.genecards.org/cgi-bin/carddisp.pl?gene=RD3
COL2A1	Collagen Type II Alpha 1 Chain	Protein Coding	P02458	62	GC12 M047 972	16.794107 4371338	https://www.genecards.org/cgi-bin/carddisp.pl?gene=COL2A1
PDE6A	Phosphodiesterase 6A	Protein Coding	P16499	57	GC05 M149 857	16.739385 6048584	https://www.genecards.org/cgi-bin/carddisp.pl?gene=PDE6A
CLRN1	Clarin 1	Protein Coding	P58418	46	GC03 M150 926	16.732906 3415527	https://www.genecards.org/cgi-bin/carddisp.pl?gene=CLRN1
MERTK	MER Proto-Oncogene, Tyrosine Kinase	Protein Coding	Q12866	63	GC02 P1118 98	16.613315 5822754	https://www.genecards.org/cgi-bin/carddisp.pl?gene=MERTK
CERKL	CERK Like Autophagy Regulator	Protein Coding	Q49MI3	50	GC02 M181 536	16.268598 5565186	https://www.genecards.org/cgi-bin/carddisp.pl?gene=CERKL
CYP4V2	Cytochrome P450 Family 4 Subfamily V Member 2	Protein Coding	Q6ZWL3	50	GC04 P1861 91	16.243530 2734375	https://www.genecards.org/cgi-bin/carddisp.pl?gene=CYP4V2
LCA5	Lebercilin LCA5	Protein Coding	Q86VQ0	49	GC06 M079 484	16.163284 3017578	https://www.genecards.org/cgi-bin/carddisp.pl?gene=LCA5
RP1L1	RP1 Like 1	Protein Coding	Q8IWN7	45	GC08 M010	16.137342 4530029	https://www.genecards.org/cgi-bin/carddisp.pl?gene=RP1L1

					606		
GRM6	Glutamate Metabotropic Receptor 6	Protein Coding	O15303	59	GC05 M183 659	15.891470 9091187	https://www.genecards.org/cgi-bin/carddisp.pl?gene=GRM6
KCNJ13	Potassium Inwardly Rectifying Channel Subfamily J Member 13	Protein Coding	O60928	53	GC02 M232 765	15.880671 5011597	https://www.genecards.org/cgi-bin/carddisp.pl?gene=KCNJ13
CABP4	Calcium Binding Protein 4	Protein Coding	P57796	47	GC11 P1151 93	15.741038 3224487	https://www.genecards.org/cgi-bin/carddisp.pl?gene=CABP4
PDE6C	Phosphodiesterase 6C	Protein Coding	P51160	57	GC10 P0936 12	15.692513 4658813	https://www.genecards.org/cgi-bin/carddisp.pl?gene=PDE6C
RBP4	Retinol Binding Protein 4	Protein Coding	P02753	57	GC10 M093 591	15.508108 1390381	https://www.genecards.org/cgi-bin/carddisp.pl?gene=RBP4
GRK1	G Protein-Coupled Receptor Kinase 1	Protein Coding	Q15835	55	GC13 P1150 40	15.504623 4130859	https://www.genecards.org/cgi-bin/carddisp.pl?gene=GRK1
UNC119	Unc-119 Lipid Binding Chaperone	Protein Coding	Q13432	55	GC17 M028 546	15.500243 1869507	https://www.genecards.org/cgi-bin/carddisp.pl?gene=UNC119
CDHR1	Cadherin Related Family Member 1	Protein Coding	Q96JP9	50	GC10 P0841 94	15.486791 6107178	https://www.genecards.org/cgi-bin/carddisp.pl?gene=CDHR1
RCVRN	Recoverin	Protein	P35243	53	GC17	15.467061	https://www.genecards.org/cgi-bin/car

		Coding			M009 896	0427856	ddisp.pl?gene=RCVRN
RGR	Retinal G Protein Coupled Receptor	Protein Coding	P47804	53	GC10 P1285 16	15.373887 0620728	https://www.genecards.org/cgi-bin/carddisp.pl?gene=RGR
TMEM67	Transmembrane Protein 67	Protein Coding	Q5HYA8	52	GC08 P0937 54	15.342782 0205688	https://www.genecards.org/cgi-bin/carddisp.pl?gene=TMEM67
IQCB1	IQ Motif Containing B1	Protein Coding	Q15051	54	GC03 M121 769	15.092672 3480225	https://www.genecards.org/cgi-bin/carddisp.pl?gene=IQCB1
RP2	RP2 Activator Of ARL3 GTPase	Protein Coding	O75695	53	GC0X P0468 37	15.072027 2064209	https://www.genecards.org/cgi-bin/carddisp.pl?gene=RP2
NMNAT1	Nicotinamide Nucleotide Adenylyltransferase 1	Protein Coding	Q9HAN9	60	GC01 P0832 52	14.992842 6742554	https://www.genecards.org/cgi-bin/carddisp.pl?gene=NMNAT1
GNAT1	G Protein Subunit Alpha Transducin 1	Protein Coding	P11488	56	GC03 P0668 93	14.957849 5025635	https://www.genecards.org/cgi-bin/carddisp.pl?gene=GNAT1
TSPAN12	Tetraspanin 12	Protein Coding	O95859	51	GC07 M120 787	14.918140 411377	https://www.genecards.org/cgi-bin/carddisp.pl?gene=TSPAN12
AHI1	Abelson Helper Integration Site 1	Protein Coding	Q8N157	53	GC06 M135 283	14.830437 6602173	https://www.genecards.org/cgi-bin/carddisp.pl?gene=AHI1

CFAP410	Cilia And Flagella Associated Protein 410	Protein Coding	O43822	44	GC21M055268	14.778491973877	https://www.genecards.org/cgi-bin/carddisp.pl?gene=CFAP410
OPN1LW	Opsin 1, Long Wave Sensitive	Protein Coding	P04000	47	GC0XP154144	14.6180629730225	https://www.genecards.org/cgi-bin/carddisp.pl?gene=OPN1LW
SALL2	Spalt Like Transcription Factor 2	Protein Coding	Q9Y467	50	GC14M021521	14.5195684432983	https://www.genecards.org/cgi-bin/carddisp.pl?gene=SALL2
CNGB1	Cyclic Nucleotide Gated Channel Subunit Beta 1	Protein Coding	Q14028	53	GC16M057884	14.496865272522	https://www.genecards.org/cgi-bin/carddisp.pl?gene=CNGB1
PDE6H	Phosphodiesterase 6H	Protein Coding	Q13956	51	GC12P080546	14.4933786392212	https://www.genecards.org/cgi-bin/carddisp.pl?gene=PDE6H
CFAP418	Cilia And Flagella Associated Protein 418	Protein Coding	Q96NL8	43	GC08M095245	14.4661798477173	https://www.genecards.org/cgi-bin/carddisp.pl?gene=CFAP418
PRPF31	Pre-MRNA Processing Factor 31	Protein Coding	Q8WWY3	55	GC19P162657	14.3972129821777	https://www.genecards.org/cgi-bin/carddisp.pl?gene=PRPF31
MAK	Male Germ Cell Associated Kinase	Protein Coding	P20794	54	GC06M010762	14.3945665359497	https://www.genecards.org/cgi-bin/carddisp.pl?gene=MAK
WHRN	Whirlin	Protein Coding	Q9P202	46	GC09M127	14.3647994995117	https://www.genecards.org/cgi-bin/carddisp.pl?gene=WHRN

					075		
CC2D2A	Coiled-Coil And C2 Domain Containing 2A	Protein Coding	Q9P2K1	52	GC04P032170	14.2939376831055	https://www.genecards.org/cgi-bin/carddisp.pl?gene=CC2D2A
CTNNB1	Catenin Beta 1	Protein Coding	P35222	66	GC03P041194	14.2788095474243	https://www.genecards.org/cgi-bin/carddisp.pl?gene=CTNNB1
ABCB6	ATP Binding Cassette Subfamily B Member 6 (LAN Blood Group)	Protein Coding	Q9NP58	57	GC02M219209	14.2187519073486	https://www.genecards.org/cgi-bin/carddisp.pl?gene=ABCB6
FAM161A	FAM161 Centrosomal Protein A	Protein Coding	Q3B820	44	GC02M061792	14.1860151290894	https://www.genecards.org/cgi-bin/carddisp.pl?gene=FAM161A
FSCN2	Fascin Actin-Bundling Protein 2, Retinal	Protein Coding	O14926	50	GC17P081515	14.0707006454468	https://www.genecards.org/cgi-bin/carddisp.pl?gene=FSCN2
PCARE	Photoreceptor Cilium Actin Regulator	Protein Coding	A6NGG8	45	GC02M029060	14.0435237884521	https://www.genecards.org/cgi-bin/carddisp.pl?gene=PCARE
KCNV2	Potassium Voltage-Gated Channel Modifier Subfamily V Member 2	Protein Coding	Q8TDN2	53	GC09P002717	14.0024175643921	https://www.genecards.org/cgi-bin/carddisp.pl?gene=KCNV2
ADGRV1	Adhesion G Protein-Coupled Receptor V1	Protein Coding	Q8WXG9	52	GC05P090529	13.8618841171265	https://www.genecards.org/cgi-bin/carddisp.pl?gene=ADGRV1
IFT140	Intraflagellar Transport 140	Protein	Q96RY7	52	GC16	13.831005	https://www.genecards.org/cgi-bin/car

		Coding			M052 123	0964355	ddisp.pl?gene=IFT140
VHL	Von Hippel-Lindau Tumor Suppressor	Protein Coding	P40337	60	GC03 P0287 87	13.800013 5421753	https://www.genecards.org/cgi-bin/car ddisp.pl?gene=VHL
CDH23	Cadherin Related 23	Protein Coding	Q9H251	56	GC10 P0713 96	13.796776 7715454	https://www.genecards.org/cgi-bin/car ddisp.pl?gene=CDH23
USH1C	USH1 Protein Network Component Harmonin	Protein Coding	Q9Y6N9	53	GC11 M018 220	13.750665 6646729	https://www.genecards.org/cgi-bin/car ddisp.pl?gene=USH1C
OPA1	OPA1 Mitochondrial Dynamin Like GTPase	Protein Coding	O60313	56	GC03 P1935 94	13.741608 6196899	https://www.genecards.org/cgi-bin/car ddisp.pl?gene=OPA1
RB1	RB Transcriptional Corepressor 1	Protein Coding	P06400	62	GC13 P0483 03	13.622934 3414307	https://www.genecards.org/cgi-bin/car ddisp.pl?gene=RB1
ALMS1	ALMS1 Centrosome And Basal Body Associated Protein	Protein Coding	Q8TCU4	51	GC02 P0733 85	13.543482 7804565	https://www.genecards.org/cgi-bin/car ddisp.pl?gene=ALMS1
CAPN5	Calpain 5	Protein Coding	O15484	56	GC11 P0770 66	13.529934 8831177	https://www.genecards.org/cgi-bin/car ddisp.pl?gene=CAPN5
NYX	Nyctalopin	Protein Coding	Q9GZU5	47	GC0X P0414 47	13.507651 3290405	https://www.genecards.org/cgi-bin/car ddisp.pl?gene=NYX

GDF6	Growth Differentiation Factor 6	Protein Coding	Q6KF10	54	GC08M096142	13.4562149047852	https://www.genecards.org/cgi-bin/carddisp.pl?gene=GDF6
AOC2	Amine Oxidase Copper Containing 2	Protein Coding	O75106	49	GC17P042844	13.4344825744629	https://www.genecards.org/cgi-bin/carddisp.pl?gene=AOC2
PRPF8	Pre-MRNA Processing Factor 8	Protein Coding	Q6P2Q9	53	GC17M001650	13.3167219161987	https://www.genecards.org/cgi-bin/carddisp.pl?gene=PRPF8
BBS4	Bardet-Biedl Syndrome 4	Protein Coding	Q96RK4	53	GC15P072686	13.3026752471924	https://www.genecards.org/cgi-bin/carddisp.pl?gene=BBS4
ARMS2	Age-Related Maculopathy Susceptibility 2	Protein Coding	P0C7Q2	40	GC10P122454	13.2305202484131	https://www.genecards.org/cgi-bin/carddisp.pl?gene=ARMS2
PAX2	Paired Box 2	Protein Coding	Q02962	58	GC10P100735	13.0073843002319	https://www.genecards.org/cgi-bin/carddisp.pl?gene=PAX2
TP53	Tumor Protein P53	Protein Coding	P04637	66	GC17M007661	12.9517040252686	https://www.genecards.org/cgi-bin/carddisp.pl?gene=TP53
TIMP3	TIMP Metallopeptidase Inhibitor 3	Protein Coding	P35625	56	GC22P097244	12.8309717178345	https://www.genecards.org/cgi-bin/carddisp.pl?gene=TIMP3
ZFYVE26	Zinc Finger FYVE-Type Containing 26	Protein Coding	Q68DK2	51	GC14M067	12.8004932403564	https://www.genecards.org/cgi-bin/carddisp.pl?gene=ZFYVE26

					727		
PDE6G	Phosphodiesterase 6G	Protein Coding	P18545	53	GC17 M081 650	12.786334 0377808	https://www.genecards.org/cgi-bin/carddisp.pl?gene=PDE6G
ARL3	ARF Like GTPase 3	Protein Coding	P36405	56	GC10 M102 673	12.785810 4705811	https://www.genecards.org/cgi-bin/carddisp.pl?gene=ARL3
PCDH15	Protocadherin Related 15	Protein Coding	Q96QU1	54	GC10 M053 802	12.724267 0059204	https://www.genecards.org/cgi-bin/carddisp.pl?gene=PCDH15
RAB28	RAB28, Member RAS Oncogene Family	Protein Coding	P51157	49	GC04 M013 361	12.689808 85	https://www.genecards.org/cgi-bin/carddisp.pl?gene=RAB28
GPR143	G Protein-Coupled Receptor 143	Protein Coding	P51810	52	GC0X M009 725	12.654562 9501343	https://www.genecards.org/cgi-bin/carddisp.pl?gene=GPR143
INPP5E	Inositol Polyphosphate-5-Phosphatase E	Protein Coding	Q9NRR6	52	GC09 M136 428	12.646861 076355	https://www.genecards.org/cgi-bin/carddisp.pl?gene=INPP5E
NPHP1	Nephrocystin 1	Protein Coding	O15259	55	GC02 M110 122	12.624832 1533203	https://www.genecards.org/cgi-bin/carddisp.pl?gene=NPHP1
MFRP	Membrane Frizzled-Related Protein	Protein Coding	Q9BY79	47	GC11 M143 869	12.589881 8969727	https://www.genecards.org/cgi-bin/carddisp.pl?gene=MFRP
LOC12680	CDK7 Strongly-Dependent Group	Function		8	GC01	12.541646	https://www.genecards.org/cgi-bin/car

5793	2 Enhancer GRCh37_chr1:94486302-94487501	al Element			P094290	0037231	ddisp.pl?gene=LOC126805793
BBS10	Bardet-Biedl Syndrome 10	Protein Coding	Q8TAM1	52	GC12M076344	12.3698606491089	https://www.genecards.org/cgi-bin/carddisp.pl?gene=BBS10
ACTG1	Actin Gamma 1	Protein Coding	P63261	60	GC17M081509	12.3286304473877	https://www.genecards.org/cgi-bin/carddisp.pl?gene=ACTG1
ARR3	Arrestin 3	Protein Coding	P36575	52	GC0XP070269	12.2959394454956	https://www.genecards.org/cgi-bin/carddisp.pl?gene=ARR3
SLC38A8	Solute Carrier Family 38 Member 8	Protein Coding	A6NNN8	45	GC16M084009	12.1845855712891	https://www.genecards.org/cgi-bin/carddisp.pl?gene=SLC38A8
BBS2	Bardet-Biedl Syndrome 2	Protein Coding	Q9BXC9	53	GC16M056467	12.0976400375366	https://www.genecards.org/cgi-bin/carddisp.pl?gene=BBS2
CHD7	Chromodomain Helicase DNA Binding Protein 7	Protein Coding	Q9P2D1	57	GC08P060678	12.0274019241333	https://www.genecards.org/cgi-bin/carddisp.pl?gene=CHD7
CLN3	CLN3 Lysosomal/Endosomal Transmembrane Protein, Battenin	Protein Coding	Q13286	56	GC16M028466	11.9901542663574	https://www.genecards.org/cgi-bin/carddisp.pl?gene=CLN3
TTC8	Tetratricopeptide Repeat Domain 8	Protein Coding	Q8TAM2	52	GC14P0959	11.9738798141479	https://www.genecards.org/cgi-bin/carddisp.pl?gene=TTC8

					12		
GPHN	Gephyrin	Protein Coding	Q9NQX3	61	GC14 P0665 07	11.954702 3773193	https://www.genecards.org/cgi-bin/carddisp.pl?gene=GPHN
RDH11	Retinol Dehydrogenase 11	Protein Coding	Q8TC12	54	GC14 M067 676	11.918107 9864502	https://www.genecards.org/cgi-bin/carddisp.pl?gene=RDH11
PRPF3	Pre-MRNA Processing Factor 3	Protein Coding	O43395	54	GC01 P1503 21	11.864038 4674072	https://www.genecards.org/cgi-bin/carddisp.pl?gene=PRPF3
COL18A1	Collagen Type XVIII Alpha 1 Chain	Protein Coding	P39060	58	GC21 P0454 05	11.805443 7637329	https://www.genecards.org/cgi-bin/carddisp.pl?gene=COL18A1
CNGA1	Cyclic Nucleotide Gated Channel Subunit Alpha 1	Protein Coding	P29973	56	GC04 M047 935	11.728147 5067139	https://www.genecards.org/cgi-bin/carddisp.pl?gene=CNGA1
RGS9	Regulator Of G Protein Signaling 9	Protein Coding	O75916	54	GC17 P1658 05	11.727878 5705566	https://www.genecards.org/cgi-bin/carddisp.pl?gene=RGS9
WDR19	WD Repeat Domain 19	Protein Coding	Q8NEZ3	50	GC04 P0401 45	11.701828 0029297	https://www.genecards.org/cgi-bin/carddisp.pl?gene=WDR19
NPHP4	Nephrocystin 4	Protein Coding	O75161	52	GC01 M023 449	11.665841 1026001	https://www.genecards.org/cgi-bin/carddisp.pl?gene=NPHP4
BDNF-AS	BDNF Antisense RNA	RNA		30	GC11	11.641999	https://www.genecards.org/cgi-bin/car

		Gene			P027466	2446899	ddisp.pl?gene=BDNF-AS
USH1G	USH1 Protein Network Component Sans	Protein Coding	Q495M9	50	GC17M074916	11.6206483840942	https://www.genecards.org/cgi-bin/carddisp.pl?gene=USH1G
RPGRIP1L	RPGRIP1 Like	Protein Coding	Q68CZ1	53	GC16M053844	11.6131534576416	https://www.genecards.org/cgi-bin/carddisp.pl?gene=RPGRIP1L
ZDHHC24	ZDHHC Palmitoyltransferase 24	Protein Coding	Q6UX98	44	GC11M143036	11.557354927063	https://www.genecards.org/cgi-bin/carddisp.pl?gene=ZDHHC24
LRIT3	Leucine Rich Repeat, Ig-Like And Transmembrane Domains 3	Protein Coding	Q3SXY7	47	GC04P109848	11.555495262146	https://www.genecards.org/cgi-bin/carddisp.pl?gene=LRIT3
TYR	Tyrosinase	Protein Coding	P14679	60	GC11P089177	11.5126523971558	https://www.genecards.org/cgi-bin/carddisp.pl?gene=TYR
BBS5	Bardet-Biedl Syndrome 5	Protein Coding	Q8N3I7	50	GC02P169502	11.4697380065918	https://www.genecards.org/cgi-bin/carddisp.pl?gene=BBS5
POC1B	POC1 Centriolar Protein B	Protein Coding	Q8TC44	47	GC12M089401	11.4286699295044	https://www.genecards.org/cgi-bin/carddisp.pl?gene=POC1B
NTRK2	Neurotrophic Receptor Tyrosine Kinase 2	Protein Coding	Q16620	66	GC09P084668	11.3709421157837	https://www.genecards.org/cgi-bin/carddisp.pl?gene=NTRK2

TRPM1	Transient Receptor Potential Cation Channel Subfamily M Member 1	Protein Coding	Q7Z4N2	56	GC15M031001	11.359899520874	https://www.genecards.org/cgi-bin/carddisp.pl?gene=TRPM1
ATF6	Activating Transcription Factor 6	Protein Coding	P18850	61	GC01P161766	11.3382434844971	https://www.genecards.org/cgi-bin/carddisp.pl?gene=ATF6
OPN1MW	Opsin 1, Medium Wave Sensitive	Protein Coding	P04001	47	GC0XP154182	11.3343734741211	https://www.genecards.org/cgi-bin/carddisp.pl?gene=OPN1MW
ATOH7	Atonal BHLH Transcription Factor 7	Protein Coding	Q8N100	49	GC10M068230	11.3267002105713	https://www.genecards.org/cgi-bin/carddisp.pl?gene=ATOH7
PTEN	Phosphatase And Tensin Homolog	Protein Coding	P60484	65	GC10P128607	11.3043041229248	https://www.genecards.org/cgi-bin/carddisp.pl?gene=PTEN
ARL6	ARF Like GTPase 6	Protein Coding	Q9H0F7	52	GC03P097764	11.2921199798584	https://www.genecards.org/cgi-bin/carddisp.pl?gene=ARL6
MKS1	MKS Transition Zone Complex Subunit 1	Protein Coding	Q9NXB0	53	GC17M058205	11.279896736145	https://www.genecards.org/cgi-bin/carddisp.pl?gene=MKS1
SNRNP200	Small Nuclear Ribonucleoprotein U5 Subunit 200	Protein Coding	O75643	55	GC02M102621	11.2604188919067	https://www.genecards.org/cgi-bin/carddisp.pl?gene=SNRNP200
TOPORS	TOP1 Binding Arginine/Serine Rich Protein, E3 Ubiquitin Ligase	Protein Coding	Q9NS56	53	GC09M032	11.2521800994873	https://www.genecards.org/cgi-bin/carddisp.pl?gene=TOPORS

					540		
MT-ND4	Mitochondrially Encoded NADH:Ubiquinone Oxidoreductase Core Subunit 4	Protein Coding	P03905	44	GCMTP010762	11.2326536178589	https://www.genecards.org/cgi-bin/carddisp.pl?gene=MT-ND4
VCAN	Versican	Protein Coding	P13611	59	GC05P083471	11.2304258346558	https://www.genecards.org/cgi-bin/carddisp.pl?gene=VCAN
SCAPER	S-Phase Cyclin A Associated Protein In The ER	Protein Coding	Q9BY12	46	GC15M076347	11.212347984314	https://www.genecards.org/cgi-bin/carddisp.pl?gene=SCAPER
PRCD	Photoreceptor Disc Component	Protein Coding	Q00LT1	40	GC17P076527	11.1901416778564	https://www.genecards.org/cgi-bin/carddisp.pl?gene=PRCD
HTRA1	HtrA Serine Peptidase 1	Protein Coding	Q92743	57	GC10P129704	11.1713151931763	https://www.genecards.org/cgi-bin/carddisp.pl?gene=HTRA1
RP9	RP9 Pre-MRNA Splicing Factor	Protein Coding	Q8TA86	47	GC07M033094	11.0943059921265	https://www.genecards.org/cgi-bin/carddisp.pl?gene=RP9
TUG1	Taurine Up-Regulated 1	Protein Coding	A0A6I8PU40	33	GC22P030969	11.0897121429443	https://www.genecards.org/cgi-bin/carddisp.pl?gene=TUG1
WT1	WT1 Transcription Factor	Protein Coding	P19544	60	GC11M032365	10.9996604919434	https://www.genecards.org/cgi-bin/carddisp.pl?gene=WT1
DRAM2	DNA Damage Regulated	Protein	Q6UX65	48	GC01	10.940381	https://www.genecards.org/cgi-bin/car

	Autophagy Modulator 2	Coding			M111 117	0501099	ddisp.pl?gene=DRAM2
BBS9	Bardet-Biedl Syndrome 9	Protein Coding	Q3SYG4	52	GC07 P0343 96	10.930414 1998291	https://www.genecards.org/cgi-bin/carddisp.pl?gene=BBS9
REEP6	Receptor Accessory Protein 6	Protein Coding	Q96HR9	49	GC19 P0014 91	10.886479 3777466	https://www.genecards.org/cgi-bin/carddisp.pl?gene=REEP6
ZNF513	Zinc Finger Protein 513	Protein Coding	Q8N8E2	45	GC02 M027 377	10.840694 4274902	https://www.genecards.org/cgi-bin/carddisp.pl?gene=ZNF513
OPN4	Opsin 4	Protein Coding	Q9UHM 6	53	GC10 P0866 54	10.817672 7294922	https://www.genecards.org/cgi-bin/carddisp.pl?gene=OPN4
TTLL5	Tubulin Tyrosine Ligase Like 5	Protein Coding	Q6EMB2	50	GC14 P0756 33	10.777497 2915649	https://www.genecards.org/cgi-bin/carddisp.pl?gene=TTLL5
SLC24A1	Solute Carrier Family 24 Member 1	Protein Coding	O60721	52	GC15 P0656 11	10.761776 9241333	https://www.genecards.org/cgi-bin/carddisp.pl?gene=SLC24A1
SLC7A14	Solute Carrier Family 7 Member 14	Protein Coding	Q8TBB6	50	GC03 M170 459	10.755434 0362549	https://www.genecards.org/cgi-bin/carddisp.pl?gene=SLC7A14
RCBTB1	RCC1 And BTB Domain Containing Protein 1	Protein Coding	Q8NDN9	50	GC13 M049 531	10.730710 9832764	https://www.genecards.org/cgi-bin/carddisp.pl?gene=RCBTB1

MT-TS2	Mitochondrially Encoded TRNA-Ser (AGU/C) 2	RNA Gene		21	GCMTP012215	10.6988258361816	https://www.genecards.org/cgi-bin/carddisp.pl?gene=MT-TS2
CWC27	CWC27 Spliceosome Associated Cyclophilin	Protein Coding	Q6UX04	52	GC05P064768	10.6606426239014	https://www.genecards.org/cgi-bin/carddisp.pl?gene=CWC27
YAP1	Yes1 Associated Transcriptional Regulator	Protein Coding	P46937	61	GC11P102110	10.6202840805054	https://www.genecards.org/cgi-bin/carddisp.pl?gene=YAP1
HGSNAT	Heparan-Alpha-Glucosaminide N-Acetyltransferase	Protein Coding	Q68CP4	48	GC08P043140	10.6065883636475	https://www.genecards.org/cgi-bin/carddisp.pl?gene=HGSNAT
IFT172	Intraflagellar Transport 172	Protein Coding	Q9UG01	52	GC02M029345	10.5984039306641	https://www.genecards.org/cgi-bin/carddisp.pl?gene=IFT172
C1QTNF5	C1q And TNF Related 5	Protein Coding	Q9BXJ0	50	GC11M119338	10.5921878814697	https://www.genecards.org/cgi-bin/carddisp.pl?gene=C1QTNF5
GPR179	G Protein-Coupled Receptor 179	Protein Coding	Q6PRD1	46	GC17M100818	10.5771780014038	https://www.genecards.org/cgi-bin/carddisp.pl?gene=GPR179
CA4	Carbonic Anhydrase 4	Protein Coding	P22748	59	GC17P060149	10.5593976974487	https://www.genecards.org/cgi-bin/carddisp.pl?gene=CA4
SIX3	SIX Homeobox 3	Protein Coding	O95343	53	GC02P0449	10.5022811889648	https://www.genecards.org/cgi-bin/carddisp.pl?gene=SIX3

					41		
CDH3	Cadherin 3	Protein Coding	P22223	59	GC16 P1298 22	10.498372 0779419	https://www.genecards.org/cgi-bin/car ddisp.pl?gene=CDH3
MFSD8	Major Facilitator Superfamily Domain Containing 8	Protein Coding	Q8NHS3	48	GC04 M127 917	10.486041 0690308	https://www.genecards.org/cgi-bin/car ddisp.pl?gene=MFSD8
FLVCR1	FLVCR Choline And Heme Transporter 1	Protein Coding	Q9Y5Y0	51	GC01 P2128 58	10.477859 4970703	https://www.genecards.org/cgi-bin/car ddisp.pl?gene=FLVCR1
BBS7	Bardet-Biedl Syndrome 7	Protein Coding	Q8IWZ6	50	GC04 M121 824	10.453635 2157593	https://www.genecards.org/cgi-bin/car ddisp.pl?gene=BBS7
SERPINF1	Serpin Family F Member 1	Protein Coding	P36955	57	GC17 P1638 67	10.435279 8461914	https://www.genecards.org/cgi-bin/car ddisp.pl?gene=SERPINF1
VEGFA	Vascular Endothelial Growth Factor A	Protein Coding	P15692	61	GC06 P0437 70	10.434260 3683472	https://www.genecards.org/cgi-bin/car ddisp.pl?gene=VEGFA
TUB	TUB Bipartite Transcription Factor	Protein Coding	P50607	56	GC11 P0080 19	10.415650 3677368	https://www.genecards.org/cgi-bin/car ddisp.pl?gene=TUB
CNNM4	Cyclin And CBS Domain Divalent Metal Cation Transport Mediator 4	Protein Coding	Q6P4Q7	51	GC02 P1008 67	10.352944 3740845	https://www.genecards.org/cgi-bin/car ddisp.pl?gene=CNNM4
SEMA4A	Semaphorin 4A	Protein	Q9H3S1	55	GC01	10.303561	https://www.genecards.org/cgi-bin/car

		Coding			P156147	2106323	ddisp.pl?gene=SEMA4A
CACNA2D4	Calcium Voltage-Gated Channel Auxiliary Subunit Alpha2delta 4	Protein Coding	Q7Z3S7	53	GC12M001791	10.281569480896	https://www.genecards.org/cgi-bin/carddisp.pl?gene=CACNA2D4
PPT1	Palmitoyl-Protein Thioesterase 1	Protein Coding	P50897	59	GC01M040289	10.2738790512085	https://www.genecards.org/cgi-bin/carddisp.pl?gene=PPT1
KLHL7	Kelch Like Family Member 7	Protein Coding	Q8IXQ5	50	GC07P023105	10.2548847198486	https://www.genecards.org/cgi-bin/carddisp.pl?gene=KLHL7
MYH10	Myosin Heavy Chain 10	Protein Coding	P35580	59	GC17M008474	10.2180585861206	https://www.genecards.org/cgi-bin/carddisp.pl?gene=MYH10
TSC2	TSC Complex Subunit 2	Protein Coding	P49815	64	GC16P128054	10.21604347	https://www.genecards.org/cgi-bin/carddisp.pl?gene=TSC2
RARB	Retinoic Acid Receptor Beta	Protein Coding	P10826	62	GC03P029063	10.21360207	https://www.genecards.org/cgi-bin/carddisp.pl?gene=RARB
NDP-AS1	NDP Antisense RNA 1	RNA Gene		21	GC0XP043949	10.2059907913208	https://www.genecards.org/cgi-bin/carddisp.pl?gene=NDP-AS1
SDHB	Succinate Dehydrogenase Complex Iron Sulfur Subunit B	Protein Coding	P21912	61	GC01M036334	10.1427097320557	https://www.genecards.org/cgi-bin/carddisp.pl?gene=SDHB

OFD1	OFD1 Centriole And Centriolar Satellite Protein	Protein Coding	O75665	55	GC0XP013714	10.1320056915283	https://www.genecards.org/cgi-bin/carddisp.pl?gene=OFD1
VPS13B	Vacuolar Protein Sorting 13 Homolog B	Protein Coding	Q7Z7G8	52	GC08P099195	10.1067819595337	https://www.genecards.org/cgi-bin/carddisp.pl?gene=VPS13B
USP45	Ubiquitin Specific Peptidase 45	Protein Coding	Q70EL2	49	GC06M099432	10.1042966842651	https://www.genecards.org/cgi-bin/carddisp.pl?gene=USP45
MT-ND1	Mitochondrially Encoded NADH:Ubiquinone Oxidoreductase Core Subunit 1	Protein Coding	P03886	46	GCMTP003309	10.1040544509888	https://www.genecards.org/cgi-bin/carddisp.pl?gene=MT-ND1
AGBL5	AGBL Carboxypeptidase 5	Protein Coding	Q8NDL9	48	GC02P030137	10.1018781661987	https://www.genecards.org/cgi-bin/carddisp.pl?gene=AGBL5
MT-ND5	Mitochondrially Encoded NADH:Ubiquinone Oxidoreductase Core Subunit 5	Protein Coding	P03915	45	GCMTP012339	10.088134765625	https://www.genecards.org/cgi-bin/carddisp.pl?gene=MT-ND5
ARSG	Arylsulfatase G	Protein Coding	Q96EG1	53	GC17P068259	10.078929901123	https://www.genecards.org/cgi-bin/carddisp.pl?gene=ARSG
GUCY2F	Guanylate Cyclase 2F, Retinal	Protein Coding	P51841	48	GC0XM109372	10.073974609375	https://www.genecards.org/cgi-bin/carddisp.pl?gene=GUCY2F
MT-ATP8	Mitochondrially Encoded ATP Synthase Membrane Subunit 8	Protein Coding	P03928	43	GCMTP0083	10.0027322769165	https://www.genecards.org/cgi-bin/carddisp.pl?gene=MT-ATP8

					68		
PRPF6	Pre-MRNA Processing Factor 6	Protein Coding	O94906	51	GC20 P0639 81	9.9933700 5615234	https://www.genecards.org/cgi-bin/carddisp.pl?gene=PRPF6
SMARCA4	SWI/SNF Related BAF Chromatin Remodeling Complex Subunit ATPase 4	Protein Coding	P51532	63	GC19 P1613 76	9.9628572 4639893	https://www.genecards.org/cgi-bin/carddisp.pl?gene=SMARCA4
PITPNM3	PITPNM Family Member 3	Protein Coding	Q9BZ71	50	GC17 M006 451	9.9619865 4174805	https://www.genecards.org/cgi-bin/carddisp.pl?gene=PITPNM3
BBS12	Bardet-Biedl Syndrome 12	Protein Coding	Q6ZW61	46	GC04 P1227 77	9.9546222 6867676	https://www.genecards.org/cgi-bin/carddisp.pl?gene=BBS12
NEUROD1	Neuronal Differentiation 1	Protein Coding	Q13562	56	GC02 M181 673	9.9260435 1043701	https://www.genecards.org/cgi-bin/carddisp.pl?gene=NEUROD1
MT-ATP6	Mitochondrially Encoded ATP Synthase Membrane Subunit 6	Protein Coding	P00846	44	GCMT P0085 31	9.9199800 491333	https://www.genecards.org/cgi-bin/carddisp.pl?gene=MT-ATP6
TSC1	TSC Complex Subunit 1	Protein Coding	Q92574	61	GC09 M132 891	9.8992719 6502686	https://www.genecards.org/cgi-bin/carddisp.pl?gene=TSC1
POMGNT1	Protein O-Linked Mannose N-Acetylglucosaminyltransferase 1 (Beta 1,2-)	Protein Coding	Q8WZA1	57	GC01 M046 188	9.8813934 3261719	https://www.genecards.org/cgi-bin/carddisp.pl?gene=POMGNT1
MIAT	Myocardial Infarction Associated	RNA		33	GC22	9.8734292	https://www.genecards.org/cgi-bin/car

	Transcript	Gene			P0266 46	9840088	ddisp.pl?gene=MIAT
MALAT1	Metastasis Associated Lung Adenocarcinoma Transcript 1	RNA Gene		33	GC11 P1150 65	9.8678731 918335	https://www.genecards.org/cgi-bin/carddisp.pl?gene=MALAT1
DICER1	Dicer 1, Ribonuclease III	Protein Coding	Q9UPY3	61	GC14 M095 086	9.8616237 6403809	https://www.genecards.org/cgi-bin/carddisp.pl?gene=DICER1
GFAP	Glial Fibrillary Acidic Protein	Protein Coding	P14136	60	GC17 M101 088	9.8228273 3917236	https://www.genecards.org/cgi-bin/carddisp.pl?gene=GFAP
CTNNA1	Catenin Alpha 1	Protein Coding	P35221	60	GC05 P1388 34	9.8184986 114502	https://www.genecards.org/cgi-bin/carddisp.pl?gene=CTNNA1
SIX6	SIX Homeobox 6	Protein Coding	O95475	52	GC14 P0624 92	9.8116168 9758301	https://www.genecards.org/cgi-bin/carddisp.pl?gene=SIX6
APOE	Apolipoprotein E	Protein Coding	P02649	62	GC19 P1622 61	9.7822093 963623	https://www.genecards.org/cgi-bin/carddisp.pl?gene=APOE
MT-TL1	Mitochondrially Encoded TRNA-Leu (UUA/G) 1	RNA Gene		24	GCMT P0032 32	9.7544164 6575928	https://www.genecards.org/cgi-bin/carddisp.pl?gene=MT-TL1
PCYT1A	Phosphate Cytidylyltransferase 1A, Choline	Protein Coding	P49585	59	GC03 M196 214	9.7436113 3575439	https://www.genecards.org/cgi-bin/carddisp.pl?gene=PCYT1A

MIR34A	MicroRNA 34a	RNA Gene		30	GC01M009151	9.69530582427979	https://www.genecards.org/cgi-bin/carddisp.pl?gene=MIR34A
MT-ND2	Mitochondrially Encoded NADH:Ubiquinone Oxidoreductase Core Subunit 2	Protein Coding	P03891	44	GCMTP004472	9.67955684661865	https://www.genecards.org/cgi-bin/carddisp.pl?gene=MT-ND2
RGS9BP	Regulator Of G Protein Signaling 9 Binding Protein	Protein Coding	Q6ZS82	43	GC19P032675	9.67445945739746	https://www.genecards.org/cgi-bin/carddisp.pl?gene=RGS9BP
KIZ	Kizuna Centrosomal Protein	Protein Coding	Q2M2Z5	47	GC20P021125	9.66599941253662	https://www.genecards.org/cgi-bin/carddisp.pl?gene=KIZ
MITF	Melanocyte Inducing Transcription Factor	Protein Coding	O75030	61	GC03P069740	9.66247081756592	https://www.genecards.org/cgi-bin/carddisp.pl?gene=MITF
USH2A-AS2	USH2A Antisense RNA 2	RNA Gene		18	GC01P216073	9.66057300567627	https://www.genecards.org/cgi-bin/carddisp.pl?gene=USH2A-AS2
DHX38	DEAH-Box Helicase 38	Protein Coding	Q92620	53	GC16P129986	9.63129997253418	https://www.genecards.org/cgi-bin/carddisp.pl?gene=DHX38
DHDDS	Dehydrodolichyl Diphosphate Synthase Subunit	Protein Coding	Q86SQ9	54	GC01P026432	9.62963485717773	https://www.genecards.org/cgi-bin/carddisp.pl?gene=DHDDS
COL9A3	Collagen Type IX Alpha 3 Chain	Protein Coding	Q14050	54	GC20P0628	9.61581134796143	https://www.genecards.org/cgi-bin/carddisp.pl?gene=COL9A3

					16		
TLCD3B	TLC Domain Containing 3B	Protein Coding	Q71RH2	46	GC16M053294	9.57430934906006	https://www.genecards.org/cgi-bin/carddisp.pl?gene=TLCD3B
ARHGEF18	Rho/Rac Guanine Nucleotide Exchange Factor 18	Protein Coding	Q6ZSZ5	51	GC19P161187	9.56125831604004	https://www.genecards.org/cgi-bin/carddisp.pl?gene=ARHGEF18
NF1	Neurofibromin 1	Protein Coding	P21359	60	GC17P031094	9.55946636199951	https://www.genecards.org/cgi-bin/carddisp.pl?gene=NF1
CDKL5	Cyclin Dependent Kinase Like 5	Protein Coding	O76039	56	GC0XP018425	9.4842472076416	https://www.genecards.org/cgi-bin/carddisp.pl?gene=CDKL5
ARL2BP	ARF Like GTPase 2 Binding Protein	Protein Coding	Q9Y2Y0	49	GC16P057245	9.47601222991943	https://www.genecards.org/cgi-bin/carddisp.pl?gene=ARL2BP
CEP78	Centrosomal Protein 78	Protein Coding	Q5JTW2	47	GC09P078236	9.47538185119629	https://www.genecards.org/cgi-bin/carddisp.pl?gene=CEP78
IFT43	Intraflagellar Transport 43	Protein Coding	Q96FT9	52	GC14P075902	9.46990776062012	https://www.genecards.org/cgi-bin/carddisp.pl?gene=IFT43
POU4F2	POU Class 4 Homeobox 2	Protein Coding	Q12837	49	GC04P146638	9.43787288665771	https://www.genecards.org/cgi-bin/carddisp.pl?gene=POU4F2
WDR37	WD Repeat Domain 37	Protein	Q9Y2I8	50	GC10	9.4256534	https://www.genecards.org/cgi-bin/car

		Coding			P002467	576416	ddisp.pl?gene=WDR37
MT-CO1	Mitochondrially Encoded Cytochrome C Oxidase I	Protein Coding	P00395	47	GCMTP005906	9.42003345489502	https://www.genecards.org/cgi-bin/carddisp.pl?gene=MT-CO1
HMCN1	Hemicentin 1	Protein Coding	Q96RW7	49	GC01P185734	9.41482257843018	https://www.genecards.org/cgi-bin/carddisp.pl?gene=HMCN1
SDHD	Succinate Dehydrogenase Complex Subunit D	Protein Coding	O14521	57	GC11P116389	9.40145111083984	https://www.genecards.org/cgi-bin/carddisp.pl?gene=SDHD
GUCA1ANB-GUCA1A	GUCA1ANB-GUCA1A Readthrough	Protein Coding		20	GC06P193529	9.37685775756836	https://www.genecards.org/cgi-bin/carddisp.pl?gene=GUCA1ANB-GUCA1A
PDE6D	Phosphodiesterase 6D	Protein Coding	O43924	56	GC02M231732	9.37591075897217	https://www.genecards.org/cgi-bin/carddisp.pl?gene=PDE6D
NF2	NF2, Moesin-Ezrin-Radixin Like (MERLIN) Tumor Suppressor	Protein Coding	P35240	62	GC22P029603	9.36932563781738	https://www.genecards.org/cgi-bin/carddisp.pl?gene=NF2
MT-ND3	Mitochondrially Encoded NADH:Ubiquinone Oxidoreductase Core Subunit 3	Protein Coding	P03897	44	GCMTP010061	9.33448314666748	https://www.genecards.org/cgi-bin/carddisp.pl?gene=MT-ND3
ABCC6	ATP Binding Cassette Subfamily C Member 6	Protein Coding	O95255	60	GC16M052674	9.28916358947754	https://www.genecards.org/cgi-bin/carddisp.pl?gene=ABCC6

KIAA1549	KIAA1549	Protein Coding	Q9HCM3	46	GC07M138831	9.26908493041992	https://www.genecards.org/cgi-bin/carddisp.pl?gene=KIAA1549
MT-CO2	Mitochondrially Encoded Cytochrome C Oxidase II	Protein Coding	P00403	50	GCMTP007587	9.24602699279785	https://www.genecards.org/cgi-bin/carddisp.pl?gene=MT-CO2
MT-CO3	Mitochondrially Encoded Cytochrome C Oxidase III	Protein Coding	P00414	44	GCMTP009209	9.22803783416748	https://www.genecards.org/cgi-bin/carddisp.pl?gene=MT-CO3
ADAM9	ADAM Metallopeptidase Domain 9	Protein Coding	Q13443	61	GC08P038996	9.20526218414307	https://www.genecards.org/cgi-bin/carddisp.pl?gene=ADAM9
TFAP2A	Transcription Factor AP-2 Alpha	Protein Coding	P05549	60	GC06M010393	9.19695377349854	https://www.genecards.org/cgi-bin/carddisp.pl?gene=TFAP2A
SDHC	Succinate Dehydrogenase Complex Subunit C	Protein Coding	Q99643	55	GC01P161314	9.1908597946167	https://www.genecards.org/cgi-bin/carddisp.pl?gene=SDHC
ELP4	Elongator Acetyltransferase Complex Subunit 4	Protein Coding	Q96EB1	51	GC11P031509	9.19044971466064	https://www.genecards.org/cgi-bin/carddisp.pl?gene=ELP4
GIGYF2	GRB10 Interacting GYF Protein 2	Protein Coding	Q6Y7W6	52	GC02P235432	9.16467761993408	https://www.genecards.org/cgi-bin/carddisp.pl?gene=GIGYF2
AHR	Aryl Hydrocarbon Receptor	Protein Coding	P35869	60	GC07P0169	9.15425586700439	https://www.genecards.org/cgi-bin/carddisp.pl?gene=AHR

					16		
CFB	Complement Factor B	Protein Coding	P00751	59	GC06P031945	9.13780879974365	https://www.genecards.org/cgi-bin/carddisp.pl?gene=CFB
MT-ND4L	Mitochondrially Encoded NADH:Ubiquinone Oxidoreductase Core Subunit 4L	Protein Coding	P03901	39	GCMTP010472	9.11622428894043	https://www.genecards.org/cgi-bin/carddisp.pl?gene=MT-ND4L
FH	Fumarate Hydratase	Protein Coding	P07954	59	GC01M241499	9.10393810272217	https://www.genecards.org/cgi-bin/carddisp.pl?gene=FH
PDE6B-AS1	PDE6B Antisense RNA 1	RNA Gene		18	GC04M006522	9.04405307769775	https://www.genecards.org/cgi-bin/carddisp.pl?gene=PDE6B-AS1
PANK2	Pantothenate Kinase 2	Protein Coding	Q9BZ23	57	GC20P011675	9.03916168212891	https://www.genecards.org/cgi-bin/carddisp.pl?gene=PANK2
PDC	Phosducin	Protein Coding	P20941	50	GC01M187190	9.00262069702148	https://www.genecards.org/cgi-bin/carddisp.pl?gene=PDC
DYNC2H1	Dynein Cytoplasmic 2 Heavy Chain 1	Protein Coding	Q8NCM8	52	GC11P103109	8.99886226654053	https://www.genecards.org/cgi-bin/carddisp.pl?gene=DYNC2H1
IFT88	Intraflagellar Transport 88	Protein Coding	Q13099	53	GC13P021420	8.99767112731934	https://www.genecards.org/cgi-bin/carddisp.pl?gene=IFT88
SHH	Sonic Hedgehog Signaling	Protein	Q15465	63	GC07	8.9960889	https://www.genecards.org/cgi-bin/car

	Molecule	Coding			M155 799	8162842	ddisp.pl?gene=SHH
MT-TS1	Mitochondrially Encoded TRNA-Ser (UCN) 1	RNA Gene		23	GCMT M007 447	8.9919328 6895752	https://www.genecards.org/cgi-bin/carddisp.pl?gene=MT-TS1
PDZD7	PDZ Domain Containing 7	Protein Coding	Q9H5P4	47	GC10 M101 007	8.9813346 862793	https://www.genecards.org/cgi-bin/carddisp.pl?gene=PDZD7
TMEM237	Transmembrane Protein 237	Protein Coding	Q96Q45	48	GC02 M201 620	8.9451427 4597168	https://www.genecards.org/cgi-bin/carddisp.pl?gene=TMEM237
CDON	Cell Adhesion Associated, Oncogene Regulated	Protein Coding	Q4KMG 0	58	GC11 M125 955	8.9332571 0296631	https://www.genecards.org/cgi-bin/carddisp.pl?gene=CDON
MT-TH	Mitochondrially Encoded TRNA-His (CAU/C)	RNA Gene		23	GCMT P0121 40	8.9182710 647583	https://www.genecards.org/cgi-bin/carddisp.pl?gene=MT-TH
CALB1	Calbindin 1	Protein Coding	P05937	57	GC08 M090 058	8.8859910 9649658	https://www.genecards.org/cgi-bin/carddisp.pl?gene=CALB1
MT-TK	Mitochondrially Encoded TRNA-Lys (AAA/G)	RNA Gene		21	GCMT P0082 97	8.8750429 1534424	https://www.genecards.org/cgi-bin/carddisp.pl?gene=MT-TK
TUBB4B	Tubulin Beta 4B Class IVb	Protein Coding	P68371	57	GC09 P1372 41	8.8748712 5396729	https://www.genecards.org/cgi-bin/carddisp.pl?gene=TUBB4B

PALS1	Protein Associated With LIN7 1, MAGUK P55 Family Member	Protein Coding	Q8N3R9	50	GC14P067240	8.87484550476074	https://www.genecards.org/cgi-bin/carddisp.pl?gene=PALS1
CFHR1	Complement Factor H Related 1	Protein Coding	Q03591	54	GC01P196822	8.82955932617188	https://www.genecards.org/cgi-bin/carddisp.pl?gene=CFHR1
FIBP	FGF1 Intracellular Binding Protein	Protein Coding	O43427	55	GC11M143005	8.82021236419678	https://www.genecards.org/cgi-bin/carddisp.pl?gene=FIBP
RET	Ret Proto-Oncogene	Protein Coding	P07949	67	GC10P053525	8.80292510986328	https://www.genecards.org/cgi-bin/carddisp.pl?gene=RET
P3H2	Prolyl 3-Hydroxylase 2	Protein Coding	Q8IVL5	53	GC03M189956	8.78727626800537	https://www.genecards.org/cgi-bin/carddisp.pl?gene=P3H2
CIB2	Calcium And Integrin Binding Family Member 2	Protein Coding	O75838	51	GC15M078104	8.77707290649414	https://www.genecards.org/cgi-bin/carddisp.pl?gene=CIB2
ABHD12	Abhydrolase Domain Containing 12, Lysophospholipase	Protein Coding	Q8N2K0	53	GC20M025294	8.76944351196289	https://www.genecards.org/cgi-bin/carddisp.pl?gene=ABHD12
KRAS	KRAS Proto-Oncogene, GTPase	Protein Coding	P01116	66	GC12M037206	8.72238349914551	https://www.genecards.org/cgi-bin/carddisp.pl?gene=KRAS
PTCH1	Patched 1	Protein Coding	Q13635	64	GC09M095	8.71820259094238	https://www.genecards.org/cgi-bin/carddisp.pl?gene=PTCH1

					442		
BAP1	BRCA1 Associated Deubiquitinase 1	Protein Coding	Q92560	59	GC03M052401	8.71312713623047	https://www.genecards.org/cgi-bin/carddisp.pl?gene=BAP1
KIAA0586	KIAA0586	Protein Coding	Q9BVV6	47	GC14P058427	8.70880889892578	https://www.genecards.org/cgi-bin/carddisp.pl?gene=KIAA0586
KIF11	Kinesin Family Member 11	Protein Coding	P52732	60	GC10P092574	8.70756435394287	https://www.genecards.org/cgi-bin/carddisp.pl?gene=KIF11
FGF2	Fibroblast Growth Factor 2	Protein Coding	P09038	59	GC04P122826	8.69928932189941	https://www.genecards.org/cgi-bin/carddisp.pl?gene=FGF2
CFHR3	Complement Factor H Related 3	Protein Coding	Q02985	53	GC01P196774	8.69388961791992	https://www.genecards.org/cgi-bin/carddisp.pl?gene=CFHR3
MSH2	MutS Homolog 2	Protein Coding	P43246	61	GC02P047403	8.69013023376465	https://www.genecards.org/cgi-bin/carddisp.pl?gene=MSH2
TMEM216	Transmembrane Protein 216	Protein Coding	Q9P0N5	46	GC11P114724	8.67067241668701	https://www.genecards.org/cgi-bin/carddisp.pl?gene=TMEM216
SDHA	Succinate Dehydrogenase Complex Flavoprotein Subunit A	Protein Coding	P31040	61	GC05P000254	8.63582992553711	https://www.genecards.org/cgi-bin/carddisp.pl?gene=SDHA
PRPF4	Pre-MRNA Splicing Tri-SnRNP	Protein	O43172	53	GC09	8.5913658	https://www.genecards.org/cgi-bin/car

	Complex Factor PRPF4	Coding			P113275	1420898	ddisp.pl?gene=PRPF4
HK1	Hexokinase 1	Protein Coding	P19367	63	GC10P069269	8.53097915649414	https://www.genecards.org/cgi-bin/carddisp.pl?gene=HK1
BMP4	Bone Morphogenetic Protein 4	Protein Coding	P12644	62	GC14M053949	8.52141094207764	https://www.genecards.org/cgi-bin/carddisp.pl?gene=BMP4
HMX1	H6 Family Homeobox 1	Protein Coding	Q9NP08	46	GC04M008847	8.50252914428711	https://www.genecards.org/cgi-bin/carddisp.pl?gene=HMX1
CP	Ceruloplasmin	Protein Coding	P00450	62	GC03M149162	8.48881244659424	https://www.genecards.org/cgi-bin/carddisp.pl?gene=CP
NPHP3	Nephrocystin 3	Protein Coding	Q7Z494	51	GC03M132684	8.43932819366455	https://www.genecards.org/cgi-bin/carddisp.pl?gene=NPHP3
SDCCAG8	SHH Signaling And Ciliogenesis Regulator SDCCAG8	Protein Coding	Q86SQ7	53	GC01P243255	8.4265251159668	https://www.genecards.org/cgi-bin/carddisp.pl?gene=SDCCAG8
MYCN	MYCN Proto-Oncogene, BHLH Transcription Factor	Protein Coding	P04198	59	GC02P016028	8.42479705810547	https://www.genecards.org/cgi-bin/carddisp.pl?gene=MYCN
BRAF	B-Raf Proto-Oncogene, Serine/Threonine Kinase	Protein Coding	P15056	67	GC07M140798	8.41872310638428	https://www.genecards.org/cgi-bin/carddisp.pl?gene=BRAF

SUFU	SUFU Negative Regulator Of Hedgehog Signaling	Protein Coding	Q9UMX1	55	GC10P128980	8.41460990905762	https://www.genecards.org/cgi-bin/carddisp.pl?gene=SUFU
IDH3B	Isocitrate Dehydrogenase (NAD(+)) 3 Non-Catalytic Subunit Beta	Protein Coding	O43837	55	GC20M002658	8.36933040618896	https://www.genecards.org/cgi-bin/carddisp.pl?gene=IDH3B
ACO2	Aconitase 2	Protein Coding	Q99798	59	GC22P097511	8.35015392303467	https://www.genecards.org/cgi-bin/carddisp.pl?gene=ACO2
MT-TI	Mitochondrially Encoded TRNA-Ile (AUU/C)	RNA Gene		21	GCMTP004265	8.32444953918457	https://www.genecards.org/cgi-bin/carddisp.pl?gene=MT-TI
CEP164	Centrosomal Protein 164	Protein Coding	Q9UPV0	53	GC11P117314	8.31486034393311	https://www.genecards.org/cgi-bin/carddisp.pl?gene=CEP164
PHYH	Phytanoyl-CoA 2-Hydroxylase	Protein Coding	O14832	56	GC10M013277	8.27995491027832	https://www.genecards.org/cgi-bin/carddisp.pl?gene=PHYH
MT-TW	Mitochondrially Encoded TRNA-Trp (UGA/G)	RNA Gene		22	GCMTP005514	8.27498531341553	https://www.genecards.org/cgi-bin/carddisp.pl?gene=MT-TW
MT-TY	Mitochondrially Encoded TRNA-Tyr (UAU/C)	RNA Gene		20	GCMTM005828	8.25189876556396	https://www.genecards.org/cgi-bin/carddisp.pl?gene=MT-TY
PEX1	Peroxisomal Biogenesis Factor 1	Protein Coding	O43933	57	GC07M092	8.2284688949585	https://www.genecards.org/cgi-bin/carddisp.pl?gene=PEX1

					487			
MT-TN	Mitochondrially Encoded TRNA-Asn (AAU/C)	RNA Gene		21	GCMTM005659	8.21554279327393	https://www.genecards.org/cgi-bin/carddisp.pl?gene=MT-TN	
MT-TQ	Mitochondrially Encoded TRNA-Gln (CAA/G)	RNA Gene		21	GCMTM004331	8.21554279327393	https://www.genecards.org/cgi-bin/carddisp.pl?gene=MT-TQ	
CCND1	Cyclin D1	Protein Coding	P24385	65	GC11P069641	8.21080780029297	https://www.genecards.org/cgi-bin/carddisp.pl?gene=CCND1	
CLCC1	Chloride Channel CLIC Like 1	Protein Coding	Q96S66	49	GC01M108881	8.17329025268555	https://www.genecards.org/cgi-bin/carddisp.pl?gene=CLCC1	
LZTFL1	Leucine Zipper Transcription Factor Like 1	Protein Coding	Q9NQ48	50	GC03M045823	8.1416482925415	https://www.genecards.org/cgi-bin/carddisp.pl?gene=LZTFL1	
SLC19A1	Solute Carrier Family 19 Member 1	Protein Coding	P41440	60	GC21M045493	8.1210470199585	https://www.genecards.org/cgi-bin/carddisp.pl?gene=SLC19A1	
RRM2B	Ribonucleotide Reductase Regulatory TP53 Inducible Subunit M2B	Protein Coding	Q7LG56	61	GC08M102204	8.12057971954346	https://www.genecards.org/cgi-bin/carddisp.pl?gene=RRM2B	
MLH1	MutL Homolog 1	Protein Coding	P40692	62	GC03P036993	8.0986270904541	https://www.genecards.org/cgi-bin/carddisp.pl?gene=MLH1	
USH2A-AS	USH2A Antisense RNA 1	RNA		19	GC01	8.0838069	https://www.genecards.org/cgi-bin/car	

1		Gene			P2161<br>93	9157715	ddisp.pl?gene=USH2A-AS1
MSH6	MutS Homolog 6	Protein Coding	P52701	61	GC02<br>P0476<br>95	8.0700902<br>9388428	https://www.genecards.org/cgi-bin/carddisp.pl?gene=MSH6
CSPP1	Centrosome And Spindle Pole Associated Protein 1	Protein Coding	Q1MSJ5	49	GC08<br>P0670<br>62	8.0607528<br>6865234	https://www.genecards.org/cgi-bin/carddisp.pl?gene=CSPP1
FBLN5	Fibulin 5	Protein Coding	Q9UBX5	59	GC14<br>M091<br>869	8.0384550<br>0946045	https://www.genecards.org/cgi-bin/carddisp.pl?gene=FBLN5
AANAT	Aralkylamine N-Acetyltransferase	Protein Coding	Q16613	52	GC17<br>P0764<br>53	8.0243310<br>9283447	https://www.genecards.org/cgi-bin/carddisp.pl?gene=AANAT
MAX	MYC Associated Factor X	Protein Coding	P61244	59	GC14<br>M065<br>451	8.0152902<br>6031494	https://www.genecards.org/cgi-bin/carddisp.pl?gene=MAX
ARL13B	ARF Like GTPase 13B	Protein Coding	Q3SXY8	50	GC03<br>P0939<br>80	8.0099191<br>6656494	https://www.genecards.org/cgi-bin/carddisp.pl?gene=ARL13B
MT-TC	Mitochondrially Encoded TRNA-Cys (UGU/C)	RNA Gene		20	GCMT<br>M005<br>763	8.0071210<br>8612061	https://www.genecards.org/cgi-bin/carddisp.pl?gene=MT-TC
MT-TG	Mitochondrially Encoded TRNA-Gly (GGN)	RNA Gene		20	GCMT<br>P0099<br>93	8.0071210<br>8612061	https://www.genecards.org/cgi-bin/carddisp.pl?gene=MT-TG

NEAT1	Nuclear Paraspeckle Assembly Transcript 1	RNA Gene		33	GC11P115059	7.98424863815308	https://www.genecards.org/cgi-bin/carddisp.pl?gene=NEAT1
CDKN1C	Cyclin Dependent Kinase Inhibitor 1C	Protein Coding	P49918	59	GC11M015881	7.97389316558838	https://www.genecards.org/cgi-bin/carddisp.pl?gene=CDKN1C
GRK7	G Protein-Coupled Receptor Kinase 7	Protein Coding	Q8WTQ7	51	GC03P144395	7.97270727157593	https://www.genecards.org/cgi-bin/carddisp.pl?gene=GRK7
HRAS	HRas Proto-Oncogene, GTPase	Protein Coding	P01112	65	GC11M015781	7.9603419303894	https://www.genecards.org/cgi-bin/carddisp.pl?gene=HRAS
PEX6	Peroxisomal Biogenesis Factor 6	Protein Coding	Q13608	53	GC06M042963	7.94436836242676	https://www.genecards.org/cgi-bin/carddisp.pl?gene=PEX6
OPN1SW	Opsin 1, Short Wave Sensitive	Protein Coding	P03999	53	GC07M128772	7.9412260055542	https://www.genecards.org/cgi-bin/carddisp.pl?gene=OPN1SW
EPCAM	Epithelial Cell Adhesion Molecule	Protein Coding	P16422	61	GC02P047345	7.93733835220337	https://www.genecards.org/cgi-bin/carddisp.pl?gene=EPCAM
PTPN11	Protein Tyrosine Phosphatase Non-Receptor Type 11	Protein Coding	Q06124	67	GC12P112418	7.86246395111084	https://www.genecards.org/cgi-bin/carddisp.pl?gene=PTPN11
KCNQ1OT1	KCNQ1 Opposite Strand/Antisense Transcript 1	RNA Gene		34	GC11M015	7.8560791015625	https://www.genecards.org/cgi-bin/carddisp.pl?gene=KCNQ1OT1

					879		
FRMD7	FERM Domain Containing 7	Protein Coding	Q6ZUT3	49	GC0XM132077	7.83368015289307	https://www.genecards.org/cgi-bin/carddisp.pl?gene=FRMD7
HOTAIR	HOX Transcript Antisense RNA	RNA Gene		31	GC12M053962	7.83185243606567	https://www.genecards.org/cgi-bin/carddisp.pl?gene=HOTAIR
C3	Complement C3	Protein Coding	P01024	62	GC19M006677	7.81005573272705	https://www.genecards.org/cgi-bin/carddisp.pl?gene=C3
MIR320A	MicroRNA 320a	RNA Gene		30	GC08M022506	7.7916407585144	https://www.genecards.org/cgi-bin/carddisp.pl?gene=MIR320A
BLOC1S1-RDH5	BLOC1S1-RDH5 Readthrough	RNA Gene		14	GC12P055716	7.77594566345215	https://www.genecards.org/cgi-bin/carddisp.pl?gene=BLOC1S1-RDH5
MEG3	Maternally Expressed 3	RNA Gene		36	GC14P122485	7.73536777496338	https://www.genecards.org/cgi-bin/carddisp.pl?gene=MEG3
BDNF	Brain Derived Neurotrophic Factor	Protein Coding	P23560	62	GC11M027654	7.73179912567139	https://www.genecards.org/cgi-bin/carddisp.pl?gene=BDNF
RUNX1	RUNX Family Transcription Factor 1	Protein Coding	Q01196	62	GC21M034787	7.72284698486328	https://www.genecards.org/cgi-bin/carddisp.pl?gene=RUNX1
MPP4	MAGUK P55 Scaffold Protein 4	Protein	Q96JB8	45	GC02	7.7170300	https://www.genecards.org/cgi-bin/car

		Coding			M201 644	4837036	ddisp.pl?gene=MPP4
POLG	DNA Polymerase Gamma, Catalytic Subunit	Protein Coding	P54098	62	GC15 M166 742	7.7107653 6178589	https://www.genecards.org/cgi-bin/car ddisp.pl?gene=POLG
MIR204	MicroRNA 204	RNA Gene		30	GC09 M070 809	7.7085456 8481445	https://www.genecards.org/cgi-bin/car ddisp.pl?gene=MIR204
MT-ND6	Mitochondrially Encoded NADH:Ubiquinone Oxidoreductase Core Subunit 6	Protein Coding	P03923	43	GCMT M014 151	7.7034645 0805664	https://www.genecards.org/cgi-bin/car ddisp.pl?gene=MT-ND6
ERCC6	ERCC Excision Repair 6, Chromatin Remodeling Factor	Protein Coding	Q03468	59	GC10 M049 692	7.6883378 0288696	https://www.genecards.org/cgi-bin/car ddisp.pl?gene=ERCC6
RLIG1	RNA 5'-Phosphate And 3'-OH Ligase 1	Protein Coding	Q8N999	42	GC12 P0880 33	7.6694989 2044067	https://www.genecards.org/cgi-bin/car ddisp.pl?gene=RLIG1
CERNA3	Competing Endogenous LncRNA 3 For MiR-645	RNA Gene		20	GC08 P0565 02	7.6631231 3079834	https://www.genecards.org/cgi-bin/car ddisp.pl?gene=CERNA3
PHF3	PHD Finger Protein 3	Protein Coding	Q92576	49	GC06 P0636 35	7.6511592 8649902	https://www.genecards.org/cgi-bin/car ddisp.pl?gene=PHF3
AKT1	AKT Serine/Threonine Kinase 1	Protein Coding	P31749	66	GC14 M104 769	7.5730409 6221924	https://www.genecards.org/cgi-bin/car ddisp.pl?gene=AKT1

CYP1B1	Cytochrome P450 Family 1 Subfamily B Member 1	Protein Coding	Q16678	60	GC02M038066	7.56866025924683	https://www.genecards.org/cgi-bin/carddisp.pl?gene=CYP1B1
RAF1	Raf-1 Proto-Oncogene, Serine/Threonine Kinase	Protein Coding	P04049	67	GC03M012583	7.56478834152222	https://www.genecards.org/cgi-bin/carddisp.pl?gene=RAF1
PVT1	Pvt1 Oncogene	RNA Gene		33	GC08P128471	7.56167459487915	https://www.genecards.org/cgi-bin/carddisp.pl?gene=PVT1
SOX2	SRY-Box Transcription Factor 2	Protein Coding	P48431	58	GC03P181711	7.55889081954956	https://www.genecards.org/cgi-bin/carddisp.pl?gene=SOX2
IDH3A	Isocitrate Dehydrogenase (NAD(+)) 3 Catalytic Subunit Alpha	Protein Coding	P50213	56	GC15P078131	7.53441095352173	https://www.genecards.org/cgi-bin/carddisp.pl?gene=IDH3A
VSX1	Visual System Homeobox 1	Protein Coding	Q9NZR4	49	GC20M025070	7.53262710571289	https://www.genecards.org/cgi-bin/carddisp.pl?gene=VSX1
MT-CYB	Mitochondrially Encoded Cytochrome B	Protein Coding	P00156	45	GCMTP014749	7.5260910987854	https://www.genecards.org/cgi-bin/carddisp.pl?gene=MT-CYB
WFS1	Wolframin ER Transmembrane Glycoprotein	Protein Coding	O76024	58	GC04P006269	7.51190996170044	https://www.genecards.org/cgi-bin/carddisp.pl?gene=WFS1
TMEM127	Transmembrane Protein 127	Protein Coding	O75204	49	GC02M096	7.47596645355225	https://www.genecards.org/cgi-bin/carddisp.pl?gene=TMEM127

					248		
C2	Complement C2	Protein Coding	P06681	59	GC06P031897	7.44977331161499	https://www.genecards.org/cgi-bin/carddisp.pl?gene=C2
SMAD4	SMAD Family Member 4	Protein Coding	Q13485	66	GC18P051028	7.44158554077148	https://www.genecards.org/cgi-bin/carddisp.pl?gene=SMAD4
EZH2	Enhancer Of Zeste 2 Polycomb Repressive Complex 2 Subunit	Protein Coding	Q15910	66	GC07M148807	7.41883897781372	https://www.genecards.org/cgi-bin/carddisp.pl?gene=EZH2
NEK2	NIMA Related Kinase 2	Protein Coding	P51955	62	GC01M211658	7.41881704330444	https://www.genecards.org/cgi-bin/carddisp.pl?gene=NEK2
KIF3B	Kinesin Family Member 3B	Protein Coding	O15066	52	GC20P032277	7.4086856842041	https://www.genecards.org/cgi-bin/carddisp.pl?gene=KIF3B
CPLANE1	Ciliogenesis And Planar Polarity Effector Complex Subunit 1	Protein Coding	Q9H799	46	GC05M039090	7.40448760986328	https://www.genecards.org/cgi-bin/carddisp.pl?gene=CPLANE1
SMARCB1	SWI/SNF Related BAF Chromatin Remodeling Complex Subunit B1	Protein Coding	Q12824	59	GC22P023786	7.38793039321899	https://www.genecards.org/cgi-bin/carddisp.pl?gene=SMARCB1
FTH1	Ferritin Heavy Chain 1	Protein Coding	P02794	63	GC11M061959	7.3692774772644	https://www.genecards.org/cgi-bin/carddisp.pl?gene=FTH1
STX3	Syntaxin 3	Protein	Q13277	54	GC11	7.3651304	https://www.genecards.org/cgi-bin/car

		Coding			P059713	2449951	ddisp.pl?gene=STX3
TRNT1	TRNA Nucleotidyl Transferase 1	Protein Coding	Q96Q11	54	GC03P003126	7.35589933395386	https://www.genecards.org/cgi-bin/carddisp.pl?gene=TRNT1
ZNF423	Zinc Finger Protein 423	Protein Coding	Q2M1K9	52	GC16M049487	7.32599258422852	https://www.genecards.org/cgi-bin/carddisp.pl?gene=ZNF423
SDHAF2	Succinate Dehydrogenase Complex Assembly Factor 2	Protein Coding	Q9NX18	53	GC11P061430	7.32064819335938	https://www.genecards.org/cgi-bin/carddisp.pl?gene=SDHAF2
MET	MET Proto-Oncogene, Receptor Tyrosine Kinase	Protein Coding	P08581	67	GC07P116672	7.31182670593262	https://www.genecards.org/cgi-bin/carddisp.pl?gene=MET
MT-TD	Mitochondrially Encoded TRNA-Asp (GAU/C)	RNA Gene		21	GCMTP007520	7.30439233779907	https://www.genecards.org/cgi-bin/carddisp.pl?gene=MT-TD
MT-TA	Mitochondrially Encoded TRNA-Ala (GCN)	RNA Gene		20	GCMTM005589	7.30439233779907	https://www.genecards.org/cgi-bin/carddisp.pl?gene=MT-TA
MT-TM	Mitochondrially Encoded TRNA-Met (AUA/G)	RNA Gene		20	GCMTP004404	7.30439233779907	https://www.genecards.org/cgi-bin/carddisp.pl?gene=MT-TM
MT-TR	Mitochondrially Encoded TRNA-Arg (CGN)	RNA Gene		20	GCMTP010407	7.30439233779907	https://www.genecards.org/cgi-bin/carddisp.pl?gene=MT-TR

VAX2	Ventral Anterior Homeobox 2	Protein Coding	Q9UIW0	49	GC02P070900	7.3002405166626	https://www.genecards.org/cgi-bin/carddisp.pl?gene=VAX2
ALDH1A3	Aldehyde Dehydrogenase 1 Family Member A3	Protein Coding	P47895	59	GC15P202880	7.28947114944458	https://www.genecards.org/cgi-bin/carddisp.pl?gene=ALDH1A3
IFNG	Interferon Gamma	Protein Coding	P01579	63	GC12M068154	7.26988935470581	https://www.genecards.org/cgi-bin/carddisp.pl?gene=IFNG
STK11	Serine/Threonine Kinase 11	Protein Coding	Q15831	62	GC19P001177	7.24717855453491	https://www.genecards.org/cgi-bin/carddisp.pl?gene=STK11
SLC6A6	Solute Carrier Family 6 Member 6	Protein Coding	P31641	58	GC03P014402	7.24630308151245	https://www.genecards.org/cgi-bin/carddisp.pl?gene=SLC6A6
PRF1	Perforin 1	Protein Coding	P14222	60	GC10M070597	7.24434852600098	https://www.genecards.org/cgi-bin/carddisp.pl?gene=PRF1
XIST	X Inactive Specific Transcript	RNA Gene		30	GC0XM073820	7.24359941482544	https://www.genecards.org/cgi-bin/carddisp.pl?gene=XIST
LARGE1	LARGE Xylosyl- And Glucuronyltransferase 1	Protein Coding	O95461	53	GC22M088338	7.24178409576416	https://www.genecards.org/cgi-bin/carddisp.pl?gene=LARGE1
TMEM231	Transmembrane Protein 231	Protein Coding	Q9H6L2	49	GC16M075	7.21913623809814	https://www.genecards.org/cgi-bin/carddisp.pl?gene=TMEM231

					536		
MKKS	MKKS Centrosomal Shuttling Protein	Protein Coding	Q9NPJ1	50	GC20M010942	7.21499919891357	https://www.genecards.org/cgi-bin/carddisp.pl?gene=MKKS
WRN	WRN RecQ Like Helicase	Protein Coding	Q14191	60	GC08P031033	7.21465015411377	https://www.genecards.org/cgi-bin/carddisp.pl?gene=WRN
INS	Insulin	Protein Coding	P01308	60	GC11M002159	7.20261573791504	https://www.genecards.org/cgi-bin/carddisp.pl?gene=INS
APC	APC Regulator Of Wnt Signaling Pathway	Protein Coding	P25054	62	GC05P112707	7.19793272018433	https://www.genecards.org/cgi-bin/carddisp.pl?gene=APC
MAP2K1	Mitogen-Activated Protein Kinase Kinase 1	Protein Coding	Q02750	67	GC15P066386	7.19379806518555	https://www.genecards.org/cgi-bin/carddisp.pl?gene=MAP2K1
PRSS23	Serine Protease 23	Protein Coding	O95084	51	GC11P086791	7.19113349914551	https://www.genecards.org/cgi-bin/carddisp.pl?gene=PRSS23
CEP250	Centrosomal Protein 250	Protein Coding	Q9BV73	51	GC20P035455	7.1611156463623	https://www.genecards.org/cgi-bin/carddisp.pl?gene=CEP250
WDPCP	WD Repeat Containing Planar Cell Polarity Effector	Protein Coding	O95876	49	GC02M063121	7.15908670425415	https://www.genecards.org/cgi-bin/carddisp.pl?gene=WDPCP
HOTTIP	HOXA Distal Transcript Antisense	RNA		31	GC07	7.1427402	https://www.genecards.org/cgi-bin/car

	RNA	Gene			P027198	4963379	ddisp.pl?gene=HOTTIP
PRKAR1A	Protein Kinase CAMP-Dependent Type I Regulatory Subunit Alpha	Protein Coding	P10644	63	GC17P165859	7.12849426269531	https://www.genecards.org/cgi-bin/carddisp.pl?gene=PRKAR1A
PMS2	PMS1 Homolog 2, Mismatch Repair System Component	Protein Coding	P54278	61	GC07M005973	7.11235666275024	https://www.genecards.org/cgi-bin/carddisp.pl?gene=PMS2
CRB2	Crumbs Cell Polarity Complex Component 2	Protein Coding	Q5IJ48	53	GC09P155632	7.10570573806763	https://www.genecards.org/cgi-bin/carddisp.pl?gene=CRB2
VIM	Vimentin	Protein Coding	P08670	63	GC10P017227	7.09545850753784	https://www.genecards.org/cgi-bin/carddisp.pl?gene=VIM
CD63	CD63 Molecule	Protein Coding	P08962	57	GC12M060634	7.07984161376953	https://www.genecards.org/cgi-bin/carddisp.pl?gene=CD63
REST	RE1 Silencing Transcription Factor	Protein Coding	Q13127	55	GC04P056907	7.07221078872681	https://www.genecards.org/cgi-bin/carddisp.pl?gene=REST
TENM3	Teneurin Transmembrane Protein 3	Protein Coding	Q9P273	48	GC04P181448	7.05841732025146	https://www.genecards.org/cgi-bin/carddisp.pl?gene=TENM3
CCAT1	Colon Cancer Associated Transcript 1	RNA Gene		23	GC08M127207	7.05812120437622	https://www.genecards.org/cgi-bin/carddisp.pl?gene=CCAT1

HIF1A	Hypoxia Inducible Factor 1 Subunit Alpha	Protein Coding	Q16665	62	GC14 P0616 95	7.0471630 0964355	https://www.genecards.org/cgi-bin/carddisp.pl?gene=HIF1A
ALK	ALK Receptor Tyrosine Kinase	Protein Coding	Q9UM73	64	GC02 M029 389	7.0390224 4567871	https://www.genecards.org/cgi-bin/carddisp.pl?gene=ALK
TTC21B	Tetratricopeptide Repeat Domain 21B	Protein Coding	Q7Z4L5	51	GC02 M165 905	7.0353388 7863159	https://www.genecards.org/cgi-bin/carddisp.pl?gene=TTC21B
TCTN1	Tectonic Family Member 1	Protein Coding	Q2MV58	50	GC12 P1106 14	7.0117864 6087646	https://www.genecards.org/cgi-bin/carddisp.pl?gene=TCTN1

Table S5: ER stress-related genes with a relevance score of not less than 7(total number of genes, 785).

Gene Symbol	Description	Category	Gifts	GC Id	Relevance score
HSPA5	Heat Shock Protein Family A (Hsp70) Member 5	Protein Coding	48	GC09M125234	71.51
EIF2AK3	Eukaryotic Translation Initiation Factor 2 Alpha Kinase 3	Protein Coding	50	GC02M088637	61.51
XBP1	X-Box Binding Protein 1	Protein Coding	47	GC22M028794	57.95
ERN1	Endoplasmic Reticulum To Nucleus Signaling 1	Protein Coding	47	GC17M064039	52.59
ATP2A2	ATPase Sarcoplasmic/Endoplasmic Reticulum Ca2+ Transporting 2	Protein Coding	52	GC12P110280	51.41
ATF6	Activating Transcription Factor 6	Protein Coding	49	GC01P161766	46.69
TP53	Tumor Protein P53	Protein Coding	55	GC17M007661	43.8
VCP	Valosin Containing Protein	Protein Coding	50	GC09M035056	42.2
HERPUD1	Homocysteine Inducible ER Protein With Ubiquitin Like Domain 1	Protein Coding	39	GC16P056931	39.04
SOD1	Superoxide Dismutase 1	Protein Coding	54	GC21P031659	38.93
RYR2	Ryanodine Receptor 2	Protein Coding	48	GC01P237042	38.55
ATP2A1	ATPase Sarcoplasmic/Endoplasmic Reticulum Ca2+ Transporting 1	Protein	49	GC16P028891	38.08

		Coding			
HSP90B1	Heat Shock Protein 90 Beta Family Member 1	Protein Coding	47	GC12P103930	36.77
TNF	Tumor Necrosis Factor	Protein Coding	53	GC06P033397	36.68
EIF2S1	Eukaryotic Translation Initiation Factor 2 Subunit Alpha	Protein Coding	46	GC14P067359	36.27
PRKN	Parkin RBR E3 Ubiquitin Protein Ligase	Protein Coding	41	GC06M161348	36.11
ATF4	Activating Transcription Factor 4	Protein Coding	48	GC22P039519	36.08
DDIT3	DNA Damage Inducible Transcript 3	Protein Coding	47	GC12M057516	35.95
APP	Amyloid Beta Precursor Protein	Protein Coding	52	GC21M025880	35.76
PSEN1	Presenilin 1	Protein Coding	53	GC14P073136	35.74
CANX	Calnexin	Protein Coding	46	GC05P179678	34.6
SYVN1	Synoviolin 1	Protein Coding	41	GC11M065122	33.49
CALR	Calreticulin	Protein Coding	52	GC19P012938	32.92
DNAJC3	DnaJ Heat Shock Protein Family (Hsp40) Member C3	Protein Coding	41	GC13P095677	31.23
HYOU1	Hypoxia Up-Regulated 1	Protein	44	GC11M119045	30.96

		Coding			
ATP2A3	ATPase Sarcoplasmic/Endoplasmic Reticulum Ca2+ Transporting 3	Protein Coding	46	GC17M003923	30.47
CAT	Catalase	Protein Coding	51	GC11P034460	29.28
INS	Insulin	Protein Coding	50	GC11M002159	29.27
SAR1B	Secretion Associated Ras Related GTPase 1B	Protein Coding	46	GC05M134601	28.59
NFE2L2	Nuclear Factor, Erythroid 2 Like 2	Protein Coding	49	GC02M177227	28.21
NFE2L1	Nuclear Factor, Erythroid 2 Like 1	Protein Coding	44	GC17P048049	27.7
RYR1	Ryanodine Receptor 1	Protein Coding	48	GC19P038434	27.69
DNAJC10	DnaJ Heat Shock Protein Family (Hsp40) Member C10	Protein Coding	37	GC02P182716	27.64
ITPR1	Inositol 1,4,5-Trisphosphate Receptor Type 1	Protein Coding	50	GC03P004486	27.62
CACNA1C	Calcium Voltage-Gated Channel Subunit Alpha1 C	Protein Coding	50	GC12P001970	27.4
WFS1	Wolframin ER Transmembrane Glycoprotein	Protein Coding	45	GC04P006271	27.2
PPP1R15A	Protein Phosphatase 1 Regulatory Subunit 15A	Protein Coding	41	GC19P048872	27.07
P4HB	Prolyl 4-Hydroxylase Subunit Beta	Protein	51	GC17M081843	27.06

		Coding			
CFTR	CF Transmembrane Conductance Regulator	Protein Coding	52	GC07P117465	26.34
PRNP	Prion Protein	Protein Coding	48	GC20P004686	26.15
MAPK8	Mitogen-Activated Protein Kinase 8	Protein Coding	51	GC10P048306	26.08
ERO1A	Endoplasmic Reticulum Oxidoreductase 1 Alpha	Protein Coding	35	GC14M052640	26.04
OS9	OS9 Endoplasmic Reticulum Lectin	Protein Coding	41	GC12P057693	25.94
DERL2	Derlin 2	Protein Coding	38	GC17M005471	25.89
BCL2	BCL2 Apoptosis Regulator	Protein Coding	53	GC18M063123	25.77
ERP29	Endoplasmic Reticulum Protein 29	Protein Coding	36	GC12P112013	25.58
SIL1	SIL1 Nucleotide Exchange Factor	Protein Coding	41	GC05M138957	25.46
CLU	Clusterin	Protein Coding	48	GC08M027596	25.31
SERP1	Stress Associated Endoplasmic Reticulum Protein 1	Protein Coding	33	GC03M150541	25.23
MAPT	Microtubule Associated Protein Tau	Protein Coding	52	GC17P045894	24.89
PDIA3	Protein Disulfide Isomerase Family A Member 3	Protein	47	GC15P043746	24.53

		Coding			
TARDBP	TAR DNA Binding Protein	Protein Coding	47	GC01P011013	24.5
MPO	Myeloperoxidase	Protein Coding	51	GC17M058269	24.43
MAP3K5	Mitogen-Activated Protein Kinase Kinase Kinase 5	Protein Coding	49	GC06M136557	23.88
H6PD	Hexose-6-Phosphate Dehydrogenase/Glucose 1-Dehydrogenase	Protein Coding	42	GC01P009234	23.87
SERPINA1	Serpin Family A Member 1	Protein Coding	51	GC14M094376	23.78
HSPA4	Heat Shock Protein Family A (Hsp70) Member 4	Protein Coding	43	GC05P133051	23.51
CASQ1	Calsequestrin 1	Protein Coding	45	GC01P160190	23.41
ERP44	Endoplasmic Reticulum Protein 44	Protein Coding	41	GC09M099979	23.29
DERL1	Derlin 1	Protein Coding	37	GC08M123013	23.22
CASP4	Caspase 4	Protein Coding	47	GC11M104942	23.07
EPM2A	EPM2A Glucan Phosphatase, Laforin	Protein Coding	43	GC06M145382	22.86
CXCL8	C-X-C Motif Chemokine Ligand 8	Protein Coding	43	GC04P073740	22.85
CASP3	Caspase 3	Protein	52	GC04M184627	22.82

		Coding			
NHLRC1	NHL Repeat Containing E3 Ubiquitin Protein Ligase 1	Protein Coding	41	GC06M018065	22.82
BAX	BCL2 Associated X, Apoptosis Regulator	Protein Coding	50	GC19P048954	22.67
ERAP1	Endoplasmic Reticulum Aminopeptidase 1	Protein Coding	45	GC05M096760	22.53
STIM1	Stromal Interaction Molecule 1	Protein Coding	50	GC11P003855	22.52
DSP	Desmoplakin	Protein Coding	51	GC06P007541	22.4
HLA-DRB1	Major Histocompatibility Complex, Class II, DR Beta 1	Protein Coding	47	GC06M032578	22.27
F2	Coagulation Factor II, Thrombin	Protein Coding	50	GC11P046720	22.22
HMOX1	Heme Oxygenase 1	Protein Coding	54	GC22P035380	21.87
KDELR1	KDEL Endoplasmic Reticulum Protein Retention Receptor 1	Protein Coding	41	GC19M048382	21.38
MIA2	MIA SH3 Domain ER Export Factor 2	Protein Coding	38	GC14P039230	21.28
SNCA	Synuclein Alpha	Protein Coding	51	GC04M089724	20.96
IL1B	Interleukin 1 Beta	Protein Coding	50	GC02M112829	20.95
DERL3	Derlin 3	Protein	36	GC22M023834	20.81

		Coding			
SEC16A	SEC16 Homolog A, Endoplasmic Reticulum Export Factor	Protein Coding	38	GC09M136440	20.8
MBTPS2	Membrane Bound Transcription Factor Peptidase, Site 2	Protein Coding	44	GC0XP021839	20.79
ERLEC1	Endoplasmic Reticulum Lectin 1	Protein Coding	37	GC02P053786	20.76
SEC23A	Sec23 Homolog A, COPII Coat Complex Component	Protein Coding	44	GC14M039031	20.75
AMFR	Autocrine Motility Factor Receptor	Protein Coding	44	GC16M056361	20.66
MANF	Mesencephalic Astrocyte Derived Neurotrophic Factor	Protein Coding	41	GC03P051385	20.53
SERP2	Stress Associated Endoplasmic Reticulum Protein Family Member 2	Protein Coding	31	GC13P044373	20.3
SEL1L	SEL1L Adaptor Subunit Of ERAD E3 Ubiquitin Ligase	Protein Coding	43	GC14M081471	19.9
APOB	Apolipoprotein B	Protein Coding	45	GC02M020956	19.83
ALB	Albumin	Protein Coding	51	GC04P073397	19.77
TRDN	Triadin	Protein Coding	41	GC06M123198	19.49
HSP90AA1	Heat Shock Protein 90 Alpha Family Class A Member 1	Protein Coding	50	GC14M102080	19.47
LMAN1	Lectin, Mannose Binding 1	Protein	45	GC18M059327	19.47

		Coding			
HSPA1A	Heat Shock Protein Family A (Hsp70) Member 1A	Protein Coding	45	GC06P033429	19.43
CDKN3	Cyclin Dependent Kinase Inhibitor 3	Protein Coding	41	GC14P054398	19.39
ERN2	Endoplasmic Reticulum To Nucleus Signaling 2	Protein Coding	39	GC16M023690	19.33
SOD2	Superoxide Dismutase 2	Protein Coding	52	GC06M159669	19.28
PDIA4	Protein Disulfide Isomerase Family A Member 4	Protein Coding	43	GC07M149003	19.12
POMC	Proopiomelanocortin	Protein Coding	50	GC02M025160	19.02
ALG2	ALG2 Alpha-1,3/1,6-Mannosyltransferase	Protein Coding	42	GC09M099216	18.82
SELENOS	Selenoprotein S	Protein Coding	32	GC15M102816	18.73
CREB3L1	CAMP Responsive Element Binding Protein 3 Like 1	Protein Coding	43	GC11P046299	18.62
APOE	Apolipoprotein E	Protein Coding	52	GC19P044906	18.57
HSPA8	Heat Shock Protein Family A (Hsp70) Member 8	Protein Coding	48	GC11M123057	18.48
HSF1	Heat Shock Transcription Factor 1	Protein Coding	46	GC08P144291	18.46
KCNH2	Potassium Voltage-Gated Channel Subfamily H Member 2	Protein	51	GC07M150944	18.4

		Coding			
HFE	Homeostatic Iron Regulator	Protein Coding	45	GC06P026087	18.38
EIF2AK4	Eukaryotic Translation Initiation Factor 2 Alpha Kinase 4	Protein Coding	46	GC15P039934	18.24
MAPK14	Mitogen-Activated Protein Kinase 14	Protein Coding	53	GC06P046047	18.23
COMT	Catechol-O-Methyltransferase	Protein Coding	52	GC22P019941	18.15
GFAP	Glial Fibrillary Acidic Protein	Protein Coding	49	GC17M044905	18.15
ELN	Elastin	Protein Coding	45	GC07P074027	17.97
CYP21A2	Cytochrome P450 Family 21 Subfamily A Member 2	Protein Coding	46	GC06P033435	17.89
KDELR2	KDEL Endoplasmic Reticulum Protein Retention Receptor 2	Protein Coding	40	GC07M006447	17.88
SEC23B	SEC23 Homolog B, COPII Coat Complex Component	Protein Coding	43	GC20P018507	17.83
BCAP31	B Cell Receptor Associated Protein 31	Protein Coding	42	GC0XM153701	17.8
CD4	CD4 Molecule	Protein Coding	51	GC12P006786	17.76
PSEN2	Presenilin 2	Protein Coding	51	GC01P226870	17.75
MAN1B1	Mannosidase Alpha Class 1B Member 1	Protein	47	GC09P137086	17.74

		Coding			
EDN1	Endothelin 1	Protein Coding	48	GC06P012290	17.72
DMD	Dystrophin	Protein Coding	48	GC0XM031047	17.7
DRD5	Dopamine Receptor D5	Protein Coding	47	GC04P009783	17.64
SEC13	SEC13 Homolog, Nuclear Pore And COPII Coat Complex Component	Protein Coding	43	GC03M010293	17.6
ERAP2	Endoplasmic Reticulum Aminopeptidase 2	Protein Coding	39	GC05P096875	17.55
ATF6B	Activating Transcription Factor 6 Beta	Protein Coding	39	GC06M032115	17.54
RPS27A	Ribosomal Protein S27a	Protein Coding	44	GC02P055231	17.49
BAG6	BAG Cochaperone 6	Protein Coding	37	GC06M031639	17.24
SPP1	Secreted Phosphoprotein 1	Protein Coding	48	GC04P087975	17.2
DNAJB9	DnaJ Heat Shock Protein Family (Hsp40) Member B9	Protein Coding	37	GC07P108569	17.09
COMP	Cartilage Oligomeric Matrix Protein	Protein Coding	48	GC19M018783	17.08
JUP	Junction Plakoglobin	Protein Coding	48	GC17M041754	17.03
ANK2	Ankyrin 2	Protein	43	GC04P112818	17.02

		Coding			
CREB3	CAMP Responsive Element Binding Protein 3	Protein Coding	39	GC09P035722	16.94
MOGS	Mannosyl-Oligosaccharide Glucosidase	Protein Coding	43	GC02M074461	16.9
SLC26A2	Solute Carrier Family 26 Member 2	Protein Coding	45	GC05P149944	16.83
SEC61A1	SEC61 Translocon Subunit Alpha 1	Protein Coding	43	GC03P128051	16.79
CR2	Complement C3d Receptor 2	Protein Coding	46	GC01P207454	16.66
CYCS	Cytochrome C, Somatic	Protein Coding	50	GC07M025118	16.57
SNAP25	Synaptosome Associated Protein 25	Protein Coding	51	GC20P010218	16.56
MAPK1	Mitogen-Activated Protein Kinase 1	Protein Coding	52	GC22M021754	16.49
SERPINH1	Serpin Family H Member 1	Protein Coding	47	GC11P075562	16.45
SCN5A	Sodium Voltage-Gated Channel Alpha Subunit 5	Protein Coding	51	GC03M038549	16.27
JUN	Jun Proto-Oncogene, AP-1 Transcription Factor Subunit	Protein Coding	51	GC01M058780	16.25
EDNRA	Endothelin Receptor Type A	Protein Coding	50	GC04P147480	16.16
PDIA2	Protein Disulfide Isomerase Family A Member 2	Protein	40	GC16P001224	16.16

		Coding			
SAR1A	Secretion Associated Ras Related GTPase 1A	Protein Coding	42	GC10M070147	16.12
DNAJB11	DnaJ Heat Shock Protein Family (Hsp40) Member B11	Protein Coding	41	GC03P186567	16.06
F9	Coagulation Factor IX	Protein Coding	47	GC0XP139530	15.87
DNAJB1	DnaJ Heat Shock Protein Family (Hsp40) Member B1	Protein Coding	45	GC19M014514	15.84
TXNDC12	Thioredoxin Domain Containing 12	Protein Coding	40	GC01M052020	15.84
SCN2A	Sodium Voltage-Gated Channel Alpha Subunit 2	Protein Coding	48	GC02P165130	15.83
NOS3	Nitric Oxide Synthase 3	Protein Coding	52	GC07P150990	15.74
CISD2	CDGSH Iron Sulfur Domain 2	Protein Coding	43	GC04P102868	15.69
EDEM1	ER Degradation Enhancing Alpha-Mannosidase Like Protein 1	Protein Coding	43	GC03P005187	15.67
GLA	Galactosidase Alpha	Protein Coding	50	GC0XM101397	15.65
SLC6A4	Solute Carrier Family 6 Member 4	Protein Coding	49	GC17M030194	15.65
SEC24C	SEC24 Homolog C, COPII Coat Complex Component	Protein Coding	45	GC10P073744	15.64
ERO1B	Endoplasmic Reticulum Oxidoreductase 1 Beta	Protein	33	GC01M236216	15.58

		Coding			
SELENOK	Selenoprotein K	Protein Coding	28	GC03M053884	15.49
PTPN1	Protein Tyrosine Phosphatase Non-Receptor Type 1	Protein Coding	52	GC20P050510	15.48
SEC24B	SEC24 Homolog B, COPII Coat Complex Component	Protein Coding	41	GC04P109433	15.46
PLN	Phospholamban	Protein Coding	45	GC06P118548	15.43
HTT	Huntingtin	Protein Coding	45	GC04P003041	15.42
SP1	Sp1 Transcription Factor	Protein Coding	45	GC12P053380	15.38
EIF2AK2	Eukaryotic Translation Initiation Factor 2 Alpha Kinase 2	Protein Coding	47	GC02M037099	15.37
GJB2	Gap Junction Protein Beta 2	Protein Coding	49	GC13M020187	15.37
MTTP	Microsomal Triglyceride Transfer Protein	Protein Coding	46	GC04P099563	15.32
UBC	Ubiquitin C	Protein Coding	44	GC12M124911	15.32
FOS	Fos Proto-Oncogene, AP-1 Transcription Factor Subunit	Protein Coding	52	GC14P075278	15.3
CASP8	Caspase 8	Protein Coding	54	GC02P201233	15.11
RTN4	Reticulon 4	Protein	46	GC02M054934	15.08

		Coding			
CREB3L2	CAMP Responsive Element Binding Protein 3 Like 2	Protein Coding	40	GC07M137874	15.07
SIRT1	Sirtuin 1	Protein Coding	51	GC10P067884	15.07
CRH	Corticotropin Releasing Hormone	Protein Coding	45	GC08M066176	15.02
KCNJ11	Potassium Inwardly Rectifying Channel Subfamily J Member 11	Protein Coding	49	GC11M017364	15.01
CASP9	Caspase 9	Protein Coding	49	GC01M015491	14.86
CREB3L3	CAMP Responsive Element Binding Protein 3 Like 3	Protein Coding	39	GC19P004153	14.85
TXNDC5	Thioredoxin Domain Containing 5	Protein Coding	40	GC06M007893	14.85
VAPB	VAMP Associated Protein B And C	Protein Coding	47	GC20P058389	14.82
PARK7	Parkinsonism Associated Deglycase	Protein Coding	47	GC01P007968	14.79
ERP27	Endoplasmic Reticulum Protein 27	Protein Coding	37	GC12M014914	14.73
TOR1A	Torsin Family 1 Member A	Protein Coding	45	GC09M129812	14.62
RTN3	Reticulon 3	Protein Coding	43	GC11P063700	14.59
SIGMAR1	Sigma Non-Opioid Intracellular Receptor 1	Protein	47	GC09M034634	14.49

		Coding			
PARP1	Poly(ADP-Ribose) Polymerase 1	Protein Coding	51	GC01M226360	14.47
IL6	Interleukin 6	Protein Coding	51	GC07P022765	14.43
UBQLN1	Ubiquilin 1	Protein Coding	44	GC09M083659	14.43
DSC2	Desmocollin 2	Protein Coding	47	GC18M031058	14.38
LMNA	Lamin A/C	Protein Coding	49	GC01P156082	14.38
TH	Tyrosine Hydroxylase	Protein Coding	53	GC11M002163	14.38
SERPINA3	Serpin Family A Member 3	Protein Coding	45	GC14P094612	14.36
RPN1	Ribophorin I	Protein Coding	43	GC03M128619	14.35
LRRK2	Leucine Rich Repeat Kinase 2	Protein Coding	51	GC12P040196	14.34
TF	Transferrin	Protein Coding	51	GC03P133666	14.32
INPP5K	Inositol Polyphosphate-5-Phosphatase K	Protein Coding	44	GC17M001494	14.31
APOA1	Apolipoprotein A1	Protein Coding	50	GC11M116835	14.29
RER1	Retention In Endoplasmic Reticulum Sorting Receptor 1	Protein	36	GC01P002391	14.26

		Coding			
UGGT1	UDP-Glucose Glycoprotein Glucosyltransferase 1	Protein Coding	39	GC02P128091	14.25
TXN	Thioredoxin	Protein Coding	47	GC09M110243	14.17
ORAI1	ORAI Calcium Release-Activated Calcium Modulator 1	Protein Coding	45	GC12P122635	14.09
CSTB	Cystatin B	Protein Coding	47	GC21M043772	14.06
CHERP	Calcium Homeostasis Endoplasmic Reticulum Protein	Protein Coding	35	GC19M016489	14.05
MAPK10	Mitogen-Activated Protein Kinase 10	Protein Coding	52	GC04M085990	14.04
BAK1	BCL2 Antagonist/Killer 1	Protein Coding	46	GC06M033572	14.01
GJA1	Gap Junction Protein Alpha 1	Protein Coding	52	GC06P121436	13.82
PPP1R15B	Protein Phosphatase 1 Regulatory Subunit 15B	Protein Coding	39	GC01M204400	13.8
AKT1	AKT Serine/Threonine Kinase 1	Protein Coding	56	GC14M104769	13.74
C1S	Complement C1s	Protein Coding	47	GC12P007884	13.73
SEC31A	SEC31 Homolog A, COPII Coat Complex Component	Protein Coding	43	GC04M082818	13.72
PTGS2	Prostaglandin-Endoperoxide Synthase 2	Protein	50	GC01M186640	13.69

		Coding			
PPIB	Peptidylprolyl Isomerase B	Protein Coding	51	GC15M064155	13.67
MTOR	Mechanistic Target Of Rapamycin Kinase	Protein Coding	54	GC01M011106	13.6
LGI4	Leucine Rich Repeat LGI Family Member 4	Protein Coding	40	GC19M035124	13.5
GSR	Glutathione-Disulfide Reductase	Protein Coding	50	GC08M030678	13.46
ALG1	ALG1 Chitobiosyldiphosphodolichol Beta-Mannosyltransferase	Protein Coding	44	GC16P005033	13.37
DPM1	Dolichyl-Phosphate Mannosyltransferase Subunit 1, Catalytic	Protein Coding	45	GC20M050934	13.35
KCNE1	Potassium Voltage-Gated Channel Subfamily E Regulatory Subunit 1	Protein Coding	45	GC21M034446	13.35
DNAH8	Dynein Axonemal Heavy Chain 8	Protein Coding	38	GC06P046078	13.32
TRAF2	TNF Receptor Associated Factor 2	Protein Coding	46	GC09P136881	13.32
CIB1	Calcium And Integrin Binding 1	Protein Coding	42	GC15M090229	13.3
AVP	Arginine Vasopressin	Protein Coding	47	GC20M003082	13.28
VEGFA	Vascular Endothelial Growth Factor A	Protein Coding	51	GC06P043770	13.28
DDOST	Dolichyl-Diphosphooligosaccharide--Protein Glycosyltransferase	Protein	45	GC01M020651	13.26

	Non-Catalytic Subunit	Coding			
SESN2	Sestrin 2	Protein Coding	39	GC01P028270	13.17
C1R	Complement C1r	Protein Coding	48	GC12M007110	13.15
IL7	Interleukin 7	Protein Coding	44	GC08M078689	13.15
LMAN2	Lectin, Mannose Binding 2	Protein Coding	39	GC05M177388	13.15
ATF3	Activating Transcription Factor 3	Protein Coding	45	GC01P212565	13.13
SSR4	Signal Sequence Receptor Subunit 4	Protein Coding	43	GC0XP153793	13.09
HMGCR	3-Hydroxy-3-Methylglutaryl-CoA Reductase	Protein Coding	47	GC05P075336	13.04
NOS2	Nitric Oxide Synthase 2	Protein Coding	51	GC17M027756	13.01
IGF2R	Insulin Like Growth Factor 2 Receptor	Protein Coding	47	GC06P159969	13
ESYT1	Extended Synaptotagmin 1	Protein Coding	39	GC12P056162	12.98
UBE2J1	Ubiquitin Conjugating Enzyme E2 J1	Protein Coding	41	GC06M089326	12.94
TFRC	Transferrin Receptor	Protein Coding	50	GC03M196027	12.93
LNPK	Lunapark, ER Junction Formation Factor	Protein	28	GC02M175924	12.86

		Coding			
HSPB1	Heat Shock Protein Family B (Small) Member 1	Protein Coding	52	GC07P076302	12.81
TRPV4	Transient Receptor Potential Cation Channel Subfamily V Member 4	Protein Coding	51	GC12M109783	12.8
COL2A1	Collagen Type II Alpha 1 Chain	Protein Coding	50	GC12M047972	12.79
VWF	Von Willebrand Factor	Protein Coding	50	GC12M005917	12.76
RNF185	Ring Finger Protein 185	Protein Coding	37	GC22P031160	12.6
HP	Haptoglobin	Protein Coding	45	GC16P072089	12.57
IAPP	Islet Amyloid Polypeptide	Protein Coding	41	GC12P021354	12.56
SERPINC1	Serpin Family C Member 1	Protein Coding	49	GC01M174131	12.53
BDNF	Brain Derived Neurotrophic Factor	Protein Coding	48	GC11M027654	12.52
NOX4	NADPH Oxidase 4	Protein Coding	44	GC11M089324	12.5
HSD11B1	Hydroxysteroid 11-Beta Dehydrogenase 1	Protein Coding	51	GC01P209686	12.43
ITPR3	Inositol 1,4,5-Trisphosphate Receptor Type 3	Protein Coding	47	GC06P033620	12.43
TMBIM6	Transmembrane BAX Inhibitor Motif Containing 6	Protein	38	GC12P049707	12.41

		Coding			
SEC62	SEC62 Homolog, Preprotein Translocation Factor	Protein Coding	37	GC03P169966	12.32
EBP	EBP Cholestenol Delta-Isomerase	Protein Coding	44	GC0XP048521	12.3
TAP1	Transporter 1, ATP Binding Cassette Subfamily B Member	Protein Coding	48	GC06M032866	12.3
GATA1	GATA Binding Protein 1	Protein Coding	47	GC0XP048786	12.28
XDH	Xanthine Dehydrogenase	Protein Coding	47	GC02M031294	12.28
CTNNB1	Catenin Beta 1	Protein Coding	54	GC03P041236	12.27
SEC61B	SEC61 Translocon Subunit Beta	Protein Coding	39	GC09P099222	12.27
SELENON	Selenoprotein N	Protein Coding	32	GC01P025800	12.27
ITPR2	Inositol 1,4,5-Trisphosphate Receptor Type 2	Protein Coding	47	GC12M026336	12.25
CASP12	Caspase 12 (Gene/Pseudogene)	Protein Coding	34	GC11M104885	12.24
TAPBP	TAP Binding Protein	Protein Coding	44	GC06M033299	12.24
CKAP4	Cytoskeleton Associated Protein 4	Protein Coding	39	GC12M106237	12.23
CLN3	CLN3 Lysosomal/Endosomal Transmembrane Protein, Battenin	Protein	44	GC16M028466	12.18

		Coding			
CNGA3	Cyclic Nucleotide Gated Channel Subunit Alpha 3	Protein Coding	44	GC02P098329	12.15
SLC8A1	Solute Carrier Family 8 Member A1	Protein Coding	45	GC02M040078	12.15
THBS1	Thrombospondin 1	Protein Coding	45	GC15P039581	12.15
BNIP1	BCL2 Interacting Protein 1	Protein Coding	37	GC05P173144	12.14
EGFR	Epidermal Growth Factor Receptor	Protein Coding	56	GC07P055019	12.13
SQSTM1	Sequestosome 1	Protein Coding	50	GC05P179806	12.12
LPL	Lipoprotein Lipase	Protein Coding	51	GC08P019901	12.04
STUB1	STIP1 Homology And U-Box Containing Protein 1	Protein Coding	47	GC16P001253	12.04
HSPA1B	Heat Shock Protein Family A (Hsp70) Member 1B	Protein Coding	41	GC06P033427	12.02
NR3C1	Nuclear Receptor Subfamily 3 Group C Member 1	Protein Coding	51	GC05M143277	12.01
RPN2	Ribophorin II	Protein Coding	43	GC20P037178	12
AKAP9	A-Kinase Anchoring Protein 9	Protein Coding	43	GC07P091940	11.96
CACNA1S	Calcium Voltage-Gated Channel Subunit Alpha1 S	Protein	48	GC01M201008	11.89

		Coding			
MYH7	Myosin Heavy Chain 7	Protein Coding	48	GC14M023412	11.86
TGFB1	Transforming Growth Factor Beta 1	Protein Coding	54	GC19M041301	11.86
NNT	Nicotinamide Nucleotide Transhydrogenase	Protein Coding	47	GC05P043638	11.83
ADIPOQ	Adiponectin, C1Q And Collagen Domain Containing	Protein Coding	47	GC03P186842	11.81
BCL2L1	BCL2 Like 1	Protein Coding	49	GC20M031664	11.8
ACP1	Acid Phosphatase 1	Protein Coding	45	GC02P000254	11.76
CST3	Cystatin C	Protein Coding	45	GC20M023608	11.75
SURF4	Surfeit 4	Protein Coding	39	GC09M133361	11.75
PON1	Paraoxonase 1	Protein Coding	47	GC07M095297	11.71
MBTPS1	Membrane Bound Transcription Factor Peptidase, Site 1	Protein Coding	44	GC16M084053	11.69
NFKB1	Nuclear Factor Kappa B Subunit 1	Protein Coding	54	GC04P102501	11.67
ABL1	ABL Proto-Oncogene 1, Non-Receptor Tyrosine Kinase	Protein Coding	54	GC09P130713	11.63
CCL2	C-C Motif Chemokine Ligand 2	Protein	50	GC17P034255	11.55

		Coding			
STING1	Stimulator Of Interferon Response CGAMP Interactor 1	Protein Coding	35	GC05M139476	11.53
BBC3	BCL2 Binding Component 3	Protein Coding	43	GC19M047220	11.5
CASQ2	Calsequestrin 2	Protein Coding	45	GC01M115700	11.47
CRYAB	Crystallin Alpha B	Protein Coding	47	GC11M111908	11.46
SEC63	SEC63 Homolog, Protein Translocation Regulator	Protein Coding	44	GC06M107867	11.43
ERLIN2	ER Lipid Raft Associated 2	Protein Coding	41	GC08P037736	11.42
ELANE	Elastase, Neutrophil Expressed	Protein Coding	48	GC19P000854	11.4
IGF2BP2	Insulin Like Growth Factor 2 MRNA Binding Protein 2	Protein Coding	44	GC03M185643	11.4
MAPK9	Mitogen-Activated Protein Kinase 9	Protein Coding	50	GC05M180234	11.4
VIM	Vimentin	Protein Coding	51	GC10P017227	11.4
TNFRSF10B	TNF Receptor Superfamily Member 10b	Protein Coding	52	GC08M023006	11.38
HLA-B	Major Histocompatibility Complex, Class I, B	Protein Coding	46	GC06M031289	11.36
INSIG1	Insulin Induced Gene 1	Protein	40	GC07P155297	11.31

		Coding			
BACE1	Beta-Secretase 1	Protein Coding	48	GC11M117285	11.29
CEBPB	CCAAT Enhancer Binding Protein Beta	Protein Coding	45	GC20P050190	11.26
PDCD6	Programmed Cell Death 6	Protein Coding	40	GC05P000272	11.26
RRBP1	Ribosome Binding Protein 1	Protein Coding	40	GC20M017613	11.24
CANT1	Calcium Activated Nucleotidase 1	Protein Coding	44	GC17M078992	11.23
MAP2	Microtubule Associated Protein 2	Protein Coding	44	GC02P209424	11.22
ERMP1	Endoplasmic Reticulum Metallopeptidase 1	Protein Coding	36	GC09M005756	11.14
UGGT2	UDP-Glucose Glycoprotein Glucosyltransferase 2	Protein Coding	37	GC13M095801	11.14
TRIM13	Tripartite Motif Containing 13	Protein Coding	37	GC13P049995	11.13
VCL	Vinculin	Protein Coding	49	GC10P073995	11.13
BRSK2	BR Serine/Threonine Kinase 2	Protein Coding	41	GC11P001389	11.12
CYBA	Cytochrome B-245 Alpha Chain	Protein Coding	47	GC16M088643	11.12
CRYAA	Crystallin Alpha A	Protein	45	GC21P043169	11.07

		Coding			
GBF1	Golgi Brefeldin A Resistant Guanine Nucleotide Exchange Factor 1	Protein Coding	44	GC10P102245	11.06
JPH2	Junctophilin 2	Protein Coding	40	GC20M044111	11.06
TRIP11	Thyroid Hormone Receptor Interactor 11	Protein Coding	43	GC14M091965	11.06
HSPA9	Heat Shock Protein Family A (Hsp70) Member 9	Protein Coding	48	GC05M138554	11.05
PDIA6	Protein Disulfide Isomerase Family A Member 6	Protein Coding	41	GC02M010774	11.01
ADAM10	ADAM Metallopeptidase Domain 10	Protein Coding	54	GC15M058588	10.97
DYSF	Dysferlin	Protein Coding	45	GC02P071453	10.97
SREBF1	Sterol Regulatory Element Binding Transcription Factor 1	Protein Coding	45	GC17M017810	10.92
TRPC1	Transient Receptor Potential Cation Channel Subfamily C Member 1	Protein Coding	43	GC03P142724	10.92
KEAP1	Kelch Like ECH Associated Protein 1	Protein Coding	48	GC19M010486	10.91
TLR4	Toll Like Receptor 4	Protein Coding	52	GC09P117704	10.91
CYP2E1	Cytochrome P450 Family 2 Subfamily E Member 1	Protein Coding	47	GC10P133520	10.89
SGK1	Serum/Glucocorticoid Regulated Kinase 1	Protein	50	GC06M134169	10.87

		Coding			
HSD11B2	Hydroxysteroid 11-Beta Dehydrogenase 2	Protein Coding	47	GC16P067433	10.86
PPARG	Peroxisome Proliferator Activated Receptor Gamma	Protein Coding	54	GC03P012287	10.86
TMEM33	Transmembrane Protein 33	Protein Coding	35	GC04P041937	10.86
UBB	Ubiquitin B	Protein Coding	44	GC17P016380	10.86
PMM2	Phosphomannomutase 2	Protein Coding	48	GC16P008788	10.83
RTN1	Reticulon 1	Protein Coding	39	GC14M059595	10.76
ESR1	Estrogen Receptor 1	Protein Coding	55	GC06P151656	10.75
FAF2	Fas Associated Factor Family Member 2	Protein Coding	39	GC05P176447	10.75
GET3	Guided Entry Of Tail-Anchored Proteins Factor 3, ATPase	Protein Coding	35	GC19P012737	10.75
HSP90AB1	Heat Shock Protein 90 Alpha Family Class B Member 1	Protein Coding	47	GC06P044246	10.75
UBE2G2	Ubiquitin Conjugating Enzyme E2 G2	Protein Coding	45	GC21M044768	10.74
G6PC	Glucose-6-Phosphatase Catalytic Subunit	Protein Coding	44	GC17P042900	10.73
PRKAA1	Protein Kinase AMP-Activated Catalytic Subunit Alpha 1	Protein	49	GC05M040759	10.69

		Coding			
EGF	Epidermal Growth Factor	Protein Coding	52	GC04P109912	10.68
CAV1	Caveolin 1	Protein Coding	50	GC07P116524	10.65
RAB1B	RAB1B, Member RAS Oncogene Family	Protein Coding	43	GC11P066270	10.64
BGLAP	Bone Gamma-Carboxyglutamate Protein	Protein Coding	41	GC01P156242	10.63
CALM1	Calmodulin 1	Protein Coding	45	GC14P090396	10.63
RAB1A	RAB1A, Member RAS Oncogene Family	Protein Coding	42	GC02M065048	10.61
ATP2C1	ATPase Secretory Pathway Ca2+ Transporting 1	Protein Coding	46	GC03P130850	10.6
EDEM3	ER Degradation Enhancing Alpha-Mannosidase Like Protein 3	Protein Coding	36	GC01M184690	10.6
MAPK3	Mitogen-Activated Protein Kinase 3	Protein Coding	51	GC16M030117	10.57
CRP	C-Reactive Protein	Protein Coding	48	GC01M159715	10.56
ADAMTSL1	ADAMTS Like 1	Protein Coding	42	GC09P017906	10.55
PLOD2	Procollagen-Lysine,2-Oxoglutarate 5-Dioxygenase 2	Protein Coding	46	GC03M146069	10.55
CD40	CD40 Molecule	Protein	50	GC20P046118	10.54

		Coding			
ENO2	Enolase 2	Protein Coding	49	GC12P006913	10.54
PIK3R1	Phosphoinositide-3-Kinase Regulatory Subunit 1	Protein Coding	52	GC05P068215	10.5
SOAT1	Sterol O-Acyltransferase 1	Protein Coding	45	GC01P179262	10.5
SREBF2	Sterol Regulatory Element Binding Transcription Factor 2	Protein Coding	44	GC22P041833	10.49
GSK3B	Glycogen Synthase Kinase 3 Beta	Protein Coding	51	GC03M119821	10.47
HSPD1	Heat Shock Protein Family D (Hsp60) Member 1	Protein Coding	49	GC02M197486	10.43
MAP1LC3A	Microtubule Associated Protein 1 Light Chain 3 Alpha	Protein Coding	45	GC20P034546	10.42
DDRGK1	DDRGK Domain Containing 1	Protein Coding	38	GC20M003191	10.39
VAPA	VAMP Associated Protein A	Protein Coding	44	GC18P009904	10.39
IFNG	Interferon Gamma	Protein Coding	50	GC12M068064	10.37
COL1A1	Collagen Type I Alpha 1 Chain	Protein Coding	52	GC17M050183	10.33
SLN	Sarcolipin	Protein Coding	33	GC11M107709	10.33
ALG13	ALG13 UDP-N-Acetylglucosaminyltransferase Subunit	Protein	38	GC0XP111665	10.31

		Coding			
CXCR4	C-X-C Motif Chemokine Receptor 4	Protein Coding	54	GC02M136114	10.31
EDEM2	ER Degradation Enhancing Alpha-Mannosidase Like Protein 2	Protein Coding	40	GC20M035115	10.3
CASP7	Caspase 7	Protein Coding	51	GC10P113679	10.24
TUSC3	Tumor Suppressor Candidate 3	Protein Coding	43	GC08P015417	10.24
PNKD	PNKD Metallo-Beta-Lactamase Domain Containing	Protein Coding	43	GC02P218270	10.21
IER3IP1	Immediate Early Response 3 Interacting Protein 1	Protein Coding	38	GC18M047152	10.2
KCNQ1	Potassium Voltage-Gated Channel Subfamily Q Member 1	Protein Coding	51	GC11P002444	10.18
LDLR	Low Density Lipoprotein Receptor	Protein Coding	50	GC19P011061	10.18
FKBP14	FKBP Prolyl Isomerase 14	Protein Coding	41	GC07M030010	10.17
AIFM1	Apoptosis Inducing Factor Mitochondria Associated 1	Protein Coding	51	GC0XM130129	10.16
IL10	Interleukin 10	Protein Coding	49	GC01M206767	10.14
HIF1A	Hypoxia Inducible Factor 1 Subunit Alpha	Protein Coding	49	GC14P061695	10.12
PRDX4	Peroxiredoxin 4	Protein	44	GC0XP023665	10.12

		Coding			
ALG3	ALG3 Alpha-1,3- Mannosyltransferase	Protein Coding	43	GC03M184244	10.11
BMP2	Bone Morphogenetic Protein 2	Protein Coding	48	GC20P006696	10.1
GAPDH	Glyceraldehyde-3-Phosphate Dehydrogenase	Protein Coding	50	GC12P006630	10.09
ALG11	ALG11 Alpha-1,2-Mannosyltransferase	Protein Coding	41	GC13P052012	10.08
FGFR3	Fibroblast Growth Factor Receptor 3	Protein Coding	56	GC04P001795	10.08
NCK1	NCK Adaptor Protein 1	Protein Coding	47	GC03P136862	10.08
BCL2L11	BCL2 Like 11	Protein Coding	47	GC02P111119	10.05
CDKN1A	Cyclin Dependent Kinase Inhibitor 1A	Protein Coding	51	GC06P046057	10.04
CYBB	Cytochrome B-245 Beta Chain	Protein Coding	48	GC0XP037780	10.04
ERGIC3	ERGIC And Golgi 3	Protein Coding	37	GC20P035542	10.03
HLA-A	Major Histocompatibility Complex, Class I, A	Protein Coding	48	GC06P033211	10.01
FICD	FIC Domain Containing	Protein Coding	34	GC12P108515	10
POR	Cytochrome P450 Oxidoreductase	Protein	50	GC07P075899	10

		Coding			
ATM	ATM Serine/Threonine Kinase	Protein Coding	55	GC11P108222	9.98
APEX1	Apurinic/Apyrimidinic Endodeoxyribonuclease 1	Protein Coding	47	GC14P020455	9.96
VCAM1	Vascular Cell Adhesion Molecule 1	Protein Coding	47	GC01P100719	9.96
NQO1	NAD(P)H Quinone Dehydrogenase 1	Protein Coding	51	GC16M069706	9.94
PTPN11	Protein Tyrosine Phosphatase Non-Receptor Type 11	Protein Coding	55	GC12P112418	9.94
PCSK9	Proprotein Convertase Subtilisin/Kexin Type 9	Protein Coding	52	GC01P055039	9.93
SLC37A4	Solute Carrier Family 37 Member 4	Protein Coding	44	GC11M119024	9.93
TRAPPC2	Trafficking Protein Particle Complex 2	Protein Coding	39	GC0XM013712	9.93
MAP2K7	Mitogen-Activated Protein Kinase Kinase 7	Protein Coding	47	GC19P007903	9.91
CPT2	Carnitine Palmitoyltransferase 2	Protein Coding	50	GC01P053196	9.9
BOK	BCL2 Family Apoptosis Regulator BOK	Protein Coding	40	GC02P241558	9.89
COL1A2	Collagen Type I Alpha 2 Chain	Protein Coding	48	GC07P094394	9.89
DNAJB12	DnaJ Heat Shock Protein Family (Hsp40) Member B12	Protein	37	GC10M072332	9.87

		Coding			
BECN1	Beclin 1	Protein Coding	48	GC17M042810	9.86
RECK	Reversion Inducing Cysteine Rich Protein With Kazal Motifs	Protein Coding	42	GC09P036036	9.86
TOMM40	Translocase Of Outer Mitochondrial Membrane 40	Protein Coding	41	GC19P044890	9.86
SRC	SRC Proto-Oncogene, Non-Receptor Tyrosine Kinase	Protein Coding	53	GC20P037344	9.84
GPR37	G Protein-Coupled Receptor 37	Protein Coding	45	GC07M124745	9.83
SERPINI1	Serpin Family I Member 1	Protein Coding	47	GC03P167735	9.83
G6PD	Glucose-6-Phosphate Dehydrogenase	Protein Coding	52	GC0XM154531	9.8
COPB1	COPI Coat Complex Subunit Beta 1	Protein Coding	39	GC11M014436	9.79
PKP2	Plakophilin 2	Protein Coding	46	GC12M032790	9.78
PRL	Prolactin	Protein Coding	44	GC06M022230	9.78
SEC61G	SEC61 Translocon Subunit Gamma	Protein Coding	37	GC07M054752	9.78
UBE2D2	Ubiquitin Conjugating Enzyme E2 D2	Protein Coding	45	GC05P139526	9.78
ABCC8	ATP Binding Cassette Subfamily C Member 8	Protein	47	GC11M017392	9.72

		Coding			
ERLIN1	ER Lipid Raft Associated 1	Protein Coding	43	GC10M100150	9.72
TEX264	Testis Expressed 264, ER-Phagy Receptor	Protein Coding	36	GC03P051663	9.72
STT3A	STT3 Oligosaccharyltransferase Complex Catalytic Subunit A	Protein Coding	45	GC11P125592	9.7
DNM1L	Dynamin 1 Like	Protein Coding	48	GC12P032679	9.68
NLRP3	NLR Family Pyrin Domain Containing 3	Protein Coding	48	GC01P247415	9.67
UBE2D3	Ubiquitin Conjugating Enzyme E2 D3	Protein Coding	45	GC04M102794	9.67
TMEM43	Transmembrane Protein 43	Protein Coding	41	GC03P014124	9.65
RUNX2	RUNX Family Transcription Factor 2	Protein Coding	48	GC06P045327	9.62
RHOA	Ras Homolog Family Member A	Protein Coding	48	GC03M049359	9.59
UBE2J2	Ubiquitin Conjugating Enzyme E2 J2	Protein Coding	42	GC01M001254	9.59
ARL6IP1	ADP Ribosylation Factor Like GTPase 6 Interacting Protein 1	Protein Coding	41	GC16M018792	9.58
NUPR1	Nuclear Protein 1, Transcriptional Regulator	Protein Coding	37	GC16M028548	9.57
TXNIP	Thioredoxin Interacting Protein	Protein	39	GC01M145992	9.57

		Coding			
TNFSF11	TNF Superfamily Member 11	Protein Coding	49	GC13P042562	9.55
PPARA	Peroxisome Proliferator Activated Receptor Alpha	Protein Coding	47	GC22P046150	9.52
GABARAPL1	GABA Type A Receptor Associated Protein Like 1	Protein Coding	44	GC12P010212	9.51
TRPM4	Transient Receptor Potential Cation Channel Subfamily M Member 4	Protein Coding	47	GC19P049157	9.51
TRAM1	Translocation Associated Membrane Protein 1	Protein Coding	40	GC08M070573	9.49
SRPRA	SRP Receptor Subunit Alpha	Protein Coding	31	GC11M126263	9.48
LIPC	Lipase C, Hepatic Type	Protein Coding	46	GC15P058410	9.46
STAU1	Staufen Double-Stranded RNA Binding Protein 1	Protein Coding	39	GC20M049113	9.44
FKRP	Fukutin Related Protein	Protein Coding	41	GC19P046746	9.43
TMEM208	Transmembrane Protein 208	Protein Coding	32	GC16P067230	9.43
UFL1	UFM1 Specific Ligase 1	Protein Coding	35	GC06P096521	9.43
STX17	Syntaxin 17	Protein Coding	36	GC09P099906	9.42
TYR	Tyrosinase	Protein	48	GC11P089177	9.41

		Coding			
MAP1LC3B	Microtubule Associated Protein 1 Light Chain 3 Beta	Protein Coding	44	GC16P087384	9.39
CLCN1	Chloride Voltage-Gated Channel 1	Protein Coding	45	GC07P143316	9.38
CRHR1	Corticotropin Releasing Hormone Receptor 1	Protein Coding	46	GC17P045784	9.37
RNF139	Ring Finger Protein 139	Protein Coding	40	GC08P124474	9.37
TCF7L2	Transcription Factor 7 Like 2	Protein Coding	47	GC10P112950	9.37
VKORC1	Vitamin K Epoxide Reductase Complex Subunit 1	Protein Coding	48	GC16M031105	9.36
HSPA6	Heat Shock Protein Family A (Hsp70) Member 6	Protein Coding	45	GC01P161524	9.34
CAPN2	Calpain 2	Protein Coding	48	GC01P223701	9.33
RB1	RB Transcriptional Corepressor 1	Protein Coding	51	GC13P048303	9.33
GANAB	Glucosidase II Alpha Subunit	Protein Coding	46	GC11M063073	9.32
NPPB	Natriuretic Peptide B	Protein Coding	45	GC01M011858	9.28
DCN	Decorin	Protein Coding	48	GC12M091140	9.26
NR3C2	Nuclear Receptor Subfamily 3 Group C Member 2	Protein	49	GC04M148078	9.26

		Coding			
OSBP	Oxysterol Binding Protein	Protein Coding	41	GC11M059818	9.25
UFD1	Ubiquitin Recognition Factor In ER Associated Degradation 1	Protein Coding	36	GC22M019450	9.25
NOTCH1	Notch Receptor 1	Protein Coding	52	GC09M136582	9.24
TMED10	Transmembrane P24 Trafficking Protein 10	Protein Coding	45	GC14M075132	9.23
HLA-DRB3	Major Histocompatibility Complex, Class II, DR Beta 3	Protein Coding	28	GC06Mn03715	9.22
IGF1	Insulin Like Growth Factor 1	Protein Coding	50	GC12M102395	9.22
TAP2	Transporter 2, ATP Binding Cassette Subfamily B Member	Protein Coding	45	GC06M032821	9.22
TMED9	Transmembrane P24 Trafficking Protein 9	Protein Coding	36	GC05P177594	9.2
FAS	Fas Cell Surface Death Receptor	Protein Coding	52	GC10P088969	9.19
MDM2	MDM2 Proto-Oncogene	Protein Coding	54	GC12P068808	9.19
BSG	Basigin (Ok Blood Group)	Protein Coding	45	GC19P000571	9.18
TMED4	Transmembrane P24 Trafficking Protein 4	Protein Coding	35	GC07M044577	9.17
RNF5	Ring Finger Protein 5	Protein	41	GC06P033439	9.16

		Coding			
DHCR24	24-Dehydrocholesterol Reductase	Protein Coding	45	GC01M054849	9.14
CCR6	C-C Motif Chemokine Receptor 6	Protein Coding	46	GC06P167111	9.13
FOXO1	Forkhead Box O1	Protein Coding	50	GC13M040555	9.13
USO1	USO1 Vesicle Transport Factor	Protein Coding	40	GC04P075724	9.12
AGR2	Anterior Gradient 2, Protein Disulphide Isomerase Family Member	Protein Coding	42	GC07M016818	9.1
ITIH4	Inter-Alpha-Trypsin Inhibitor Heavy Chain 4	Protein Coding	44	GC03M052812	9.1
TRPC4	Transient Receptor Potential Cation Channel Subfamily C Member 4	Protein Coding	46	GC13M037636	9.1
CD8A	CD8a Molecule	Protein Coding	48	GC02M086784	9.09
LEP	Leptin	Protein Coding	49	GC07P128241	9.08
TIA1	TIA1 Cytotoxic Granule Associated RNA Binding Protein	Protein Coding	44	GC02M070209	9.08
SCAP	SREBF Chaperone	Protein Coding	41	GC03M047413	9.07
ZFAND2B	Zinc Finger AN1-Type Containing 2B	Protein Coding	36	GC02P219195	9.07
OSBPL8	Oxysterol Binding Protein Like 8	Protein	39	GC12M076354	9.06

		Coding			
TMED2	Transmembrane P24 Trafficking Protein 2	Protein Coding	37	GC12P123584	9.04
MAN2A1	Mannosidase Alpha Class 2A Member 1	Protein Coding	43	GC05P109689	9.03
UBQLN2	Ubiquilin 2	Protein Coding	44	GC0XP056563	9.01
NPLOC4	NPL4 Homolog, Ubiquitin Recognition Factor	Protein Coding	40	GC17M081556	9
RNF186	Ring Finger Protein 186	Protein Coding	33	GC01M019814	9
KTN1	Kinectin 1	Protein Coding	40	GC14P055559	8.99
PRKAA2	Protein Kinase AMP-Activated Catalytic Subunit Alpha 2	Protein Coding	51	GC01P056645	8.95
FABP1	Fatty Acid Binding Protein 1	Protein Coding	45	GC02M088122	8.94
C9orf72	C9orf72-SMCR8 Complex Subunit	Protein Coding	42	GC09M027539	8.93
CP	Ceruloplasmin	Protein Coding	49	GC03M149162	8.93
TMX1	Thioredoxin Related Transmembrane Protein 1	Protein Coding	39	GC14P051240	8.92
ARSA	Arylsulfatase A	Protein Coding	48	GC22M050622	8.9
CALHM1	Calcium Homeostasis Modulator 1	Protein	35	GC10M103454	8.9

		Coding			
CUL1	Cullin 1	Protein Coding	47	GC07P148697	8.9
G3BP1	G3BP Stress Granule Assembly Factor 1	Protein Coding	41	GC05P151771	8.9
GCG	Glucagon	Protein Coding	42	GC02M162142	8.9
MATN3	Matrilin 3	Protein Coding	44	GC02M019992	8.9
TMEM214	Transmembrane Protein 214	Protein Coding	35	GC02P026997	8.9
RAB6A	RAB6A, Member RAS Oncogene Family	Protein Coding	42	GC11M073676	8.87
RAC1	Rac Family Small GTPase 1	Protein Coding	51	GC07P006380	8.87
CCDC47	Coiled-Coil Domain Containing 47	Protein Coding	35	GC17M063745	8.86
TMTC3	Transmembrane O-Mannosyltransferase Targeting Cadherins 3	Protein Coding	37	GC12P088142	8.86
ACTC1	Actin Alpha Cardiac Muscle 1	Protein Coding	43	GC15M034788	8.85
CASP2	Caspase 2	Protein Coding	50	GC07P144746	8.84
ORMDL3	ORMDL Sphingolipid Biosynthesis Regulator 3	Protein Coding	40	GC17M039921	8.84
RNFT1	Ring Finger Protein, Transmembrane 1	Protein	36	GC17M059952	8.84

		Coding			
GPT	Glutamic--Pyruvic Transaminase	Protein Coding	42	GC08P144502	8.83
PON2	Paraoxonase 2	Protein Coding	44	GC07M095404	8.83
HMGB1	High Mobility Group Box 1	Protein Coding	45	GC13M030456	8.82
MGAT5	Alpha-1,6-Mannosylglycoprotein 6-Beta-N-Acetylglucosaminyltransferase	Protein Coding	40	GC02P134119	8.82
SSR2	Signal Sequence Receptor Subunit 2	Protein Coding	41	GC01M156009	8.82
JPH4	Junctophilin 4	Protein Coding	35	GC14M023568	8.81
UFM1	Ubiquitin Fold Modifier 1	Protein Coding	41	GC13P038349	8.81
PTGS1	Prostaglandin-Endoperoxide Synthase 1	Protein Coding	48	GC09P122370	8.8
TRIB3	Tribbles Pseudokinase 3	Protein Coding	45	GC20P000361	8.8
ASPH	Aspartate Beta-Hydroxylase	Protein Coding	44	GC08M061500	8.78
MYOC	Myocilin	Protein Coding	43	GC01M171604	8.78
CREB1	CAMP Responsive Element Binding Protein 1	Protein Coding	50	GC02P207529	8.76
PIK3CG	Phosphatidylinositol-4,5-Bisphosphate 3-Kinase Catalytic Subunit	Protein	49	GC07P106865	8.76

	Gamma	Coding			
HTRA2	HtrA Serine Peptidase 2	Protein Coding	48	GC02P074529	8.75
TFG	Trafficking From ER To Golgi Regulator	Protein Coding	46	GC03P100709	8.75
ANXA5	Annexin A5	Protein Coding	48	GC04M121667	8.73
GABARAP	GABA Type A Receptor-Associated Protein	Protein Coding	45	GC17M007240	8.71
ARSG	Arylsulfatase G	Protein Coding	44	GC17P068326	8.7
ITGB1	Integrin Subunit Beta 1	Protein Coding	51	GC10M032900	8.69
MAN1A1	Mannosidase Alpha Class 1A Member 1	Protein Coding	40	GC06M119269	8.69
PRKCD	Protein Kinase C Delta	Protein Coding	55	GC03P053156	8.69
AGER	Advanced Glycosylation End-Product Specific Receptor	Protein Coding	45	GC06M032180	8.68
CYP1A1	Cytochrome P450 Family 1 Subfamily A Member 1	Protein Coding	48	GC15M074719	8.68
INSR	Insulin Receptor	Protein Coding	55	GC19M007112	8.67
PPARGC1A	PPARG Coactivator 1 Alpha	Protein Coding	48	GC04M023755	8.65
SCAMP5	Secretory Carrier Membrane Protein 5	Protein	37	GC15P074957	8.65

		Coding			
STAT3	Signal Transducer And Activator Of Transcription 3	Protein Coding	54	GC17M042313	8.64
TTN	Titin	Protein Coding	48	GC02M178525	8.64
NPY	Neuropeptide Y	Protein Coding	47	GC07P024290	8.63
TG	Thyroglobulin	Protein Coding	43	GC08P132866	8.61
TMCO1	Transmembrane And Coiled-Coil Domains 1	Protein Coding	41	GC01M165724	8.61
FOXO3	Forkhead Box O3	Protein Coding	45	GC06P108559	8.59
DNAJA1	DnaJ Heat Shock Protein Family (Hsp40) Member A1	Protein Coding	43	GC09P033025	8.58
PIEZO1	Piezo Type Mechanosensitive Ion Channel Component 1	Protein Coding	39	GC16M088715	8.57
BAG1	BAG Cochaperone 1	Protein Coding	44	GC09M033245	8.56
RPSA	Ribosomal Protein SA	Protein Coding	46	GC03P039406	8.56
KNG1	Kininogen 1	Protein Coding	45	GC03P186717	8.54
RYR3	Ryanodine Receptor 3	Protein Coding	43	GC15P033310	8.54
TOR1B	Torsin Family 1 Member B	Protein	39	GC09P129803	8.54

		Coding			
SSR1	Signal Sequence Receptor Subunit 1	Protein Coding	41	GC06M007268	8.53
ADRB2	Adrenoceptor Beta 2	Protein Coding	50	GC05P148825	8.52
NOS1	Nitric Oxide Synthase 1	Protein Coding	50	GC12M117208	8.51
IREB2	Iron Responsive Element Binding Protein 2	Protein Coding	44	GC15P078437	8.5
P4HTM	Prolyl 4-Hydroxylase, Transmembrane	Protein Coding	37	GC03P049150	8.5
AGR3	Anterior Gradient 3, Protein Disulphide Isomerase Family Member	Protein Coding	39	GC07M016854	8.49
CDIPT	CDP-Diacylglycerol--Inositol 3-Phosphatidyltransferase	Protein Coding	42	GC16M029870	8.49
SRP68	Signal Recognition Particle 68	Protein Coding	36	GC17M076038	8.49
CCDC88B	Coiled-Coil Domain Containing 88B	Protein Coding	33	GC11P064340	8.48
CERT1	Ceramide Transporter 1	Protein Coding	35	GC05M075356	8.43
LRP5	LDL Receptor Related Protein 5	Protein Coding	50	GC11P068298	8.43
NGF	Nerve Growth Factor	Protein Coding	52	GC01M115285	8.42
RAB10	RAB10, Member RAS Oncogene Family	Protein	44	GC02P026033	8.4

		Coding			
TLR9	Toll Like Receptor 9	Protein Coding	46	GC03M052222	8.4
SHH	Sonic Hedgehog Signaling Molecule	Protein Coding	50	GC07M155799	8.39
MMP9	Matrix Metallopeptidase 9	Protein Coding	54	GC20P046008	8.36
UGT1A1	UDP Glucuronosyltransferase Family 1 Member A1	Protein Coding	50	GC02P233760	8.36
PINK1	PTEN Induced Kinase 1	Protein Coding	48	GC01P020634	8.34
ERGIC2	ERGIC And Golgi 2	Protein Coding	37	GC12M029337	8.33
SERPINA7	Serpin Family A Member 7	Protein Coding	40	GC0XM106032	8.33
YKT6	YKT6 V-SNARE Homolog	Protein Coding	41	GC07P044200	8.33
SEC22B	SEC22 Homolog B, Vesicle Trafficking Protein (Gene/Pseudogene)	Protein Coding	36	GC01M120150	8.32
SGPP1	Sphingosine-1-Phosphate Phosphatase 1	Protein Coding	40	GC14M063684	8.31
MGST1	Microsomal Glutathione S-Transferase 1	Protein Coding	44	GC12P016347	8.3
RBX1	Ring-Box 1	Protein Coding	44	GC22P040951	8.3
STARD3	StAR Related Lipid Transfer Domain Containing 3	Protein	40	GC17P039637	8.29

		Coding			
CD36	CD36 Molecule	Protein Coding	50	GC07P080369	8.28
ESYT2	Extended Synaptotagmin 2	Protein Coding	37	GC07M158730	8.27
GBA	Glucosylceramidase Beta	Protein Coding	49	GC01M155234	8.27
SCG5	Secretogranin V	Protein Coding	39	GC15P032641	8.26
KDR	Kinase Insert Domain Receptor	Protein Coding	55	GC04M055078	8.24
SCD	Stearoyl-CoA Desaturase	Protein Coding	49	GC10P100347	8.24
TERT	Telomerase Reverse Transcriptase	Protein Coding	53	GC05M001253	8.24
PKD2	Polycystin 2, Transient Receptor Potential Cation Channel	Protein Coding	47	GC04P088007	8.23
CREBRF	CREB3 Regulatory Factor	Protein Coding	32	GC05P173056	8.22
GSTM1	Glutathione S-Transferase Mu 1	Protein Coding	43	GC01P109687	8.22
GPX1	Glutathione Peroxidase 1	Protein Coding	49	GC03M049370	8.21
RHBDD1	Rhomboid Domain Containing 1	Protein Coding	36	GC02P226835	8.21
TGM2	Transglutaminase 2	Protein	50	GC20M038127	8.21

		Coding			
UBE4B	Ubiquitination Factor E4B	Protein Coding	41	GC01P010032	8.21
DSG2	Desmoglein 2	Protein Coding	47	GC18P031498	8.2
STX18	Syntaxin 18	Protein Coding	38	GC04M004417	8.19
COL9A1	Collagen Type IX Alpha 1 Chain	Protein Coding	43	GC06M070215	8.18
MARCHF6	Membrane Associated Ring-CH-Type Finger 6	Protein Coding	29	GC05P010356	8.18
FKBP10	FKBP Prolyl Isomerase 10	Protein Coding	41	GC17P041812	8.17
UBA52	Ubiquitin A-52 Residue Ribosomal Protein Fusion Product 1	Protein Coding	43	GC19P018563	8.14
UBE2D1	Ubiquitin Conjugating Enzyme E2 D1	Protein Coding	47	GC10P058334	8.14
WNT1	Wnt Family Member 1	Protein Coding	48	GC12P049041	8.14
CDK1	Cyclin Dependent Kinase 1	Protein Coding	47	GC10P060772	8.13
DES	Desmin	Protein Coding	49	GC02P219418	8.13
HSPA1L	Heat Shock Protein Family A (Hsp70) Member 1 Like	Protein Coding	44	GC06M031809	8.13
ACAN	Aggrecan	Protein	47	GC15P088814	8.1

		Coding			
ATXN3	Ataxin 3	Protein Coding	45	GC14M093534	8.09
ACE	Angiotensin I Converting Enzyme	Protein Coding	51	GC17P063477	8.08
JPH3	Junctophilin 3	Protein Coding	42	GC16P087601	8.08
UBQLN4	Ubiquilin 4	Protein Coding	41	GC01M156033	8.08
ARSH	Arylsulfatase Family Member H	Protein Coding	35	GC0XP003006	8.07
FBXO6	F-Box Protein 6	Protein Coding	36	GC01P011664	8.06
FUS	FUS RNA Binding Protein	Protein Coding	45	GC16P031418	8.06
GUSB	Glucuronidase Beta	Protein Coding	48	GC07M065960	8.06
PML	Promyelocytic Leukemia	Protein Coding	47	GC15P073994	8.06
AGTR1	Angiotensin II Receptor Type 1	Protein Coding	51	GC03P148697	8.04
SEC23IP	SEC23 Interacting Protein	Protein Coding	39	GC10P119892	8.02
TTR	Transthyretin	Protein Coding	51	GC18P031557	8.02
AUP1	AUP1 Lipid Droplet Regulating VLDL Assembly Factor	Protein	38	GC02M074526	8.01

		Coding			
NGLY1	N-Glycanase 1	Protein Coding	44	GC03M025718	8
PRKCSH	Protein Kinase C Substrate 80K-H	Protein Coding	45	GC19P011435	8
IVL	Involucrin	Protein Coding	39	GC01P152881	7.99
UBXN4	UBX Domain Protein 4	Protein Coding	36	GC02P135741	7.99
JKAMP	JNK1/MAPK8 Associated Membrane Protein	Protein Coding	35	GC14P059484	7.98
HSPA2	Heat Shock Protein Family A (Hsp70) Member 2	Protein Coding	45	GC14P064535	7.96
TNFSF10	TNF Superfamily Member 10	Protein Coding	48	GC03M172505	7.96
GET4	Guided Entry Of Tail-Anchored Proteins Factor 4	Protein Coding	35	GC07P000876	7.94
HSD17B10	Hydroxysteroid 17-Beta Dehydrogenase 10	Protein Coding	47	GC0XM053431	7.94
DAB2IP	DAB2 Interacting Protein	Protein Coding	40	GC09P121566	7.93
UBL4A	Ubiquitin Like 4A	Protein Coding	36	GC0XM154483	7.93
BSCL2	BSCL2 Lipid Droplet Biogenesis Associated, Seipin	Protein Coding	43	GC11M063080	7.92
GSTP1	Glutathione S-Transferase Pi 1	Protein	51	GC11P067583	7.92

		Coding			
MFN2	Mitofusin 2	Protein Coding	50	GC01P011980	7.92
UBE2K	Ubiquitin Conjugating Enzyme E2 K	Protein Coding	43	GC04P039700	7.91
CAMK2G	Calcium/Calmodulin Dependent Protein Kinase II Gamma	Protein Coding	48	GC10M073812	7.9
TMEM117	Transmembrane Protein 117	Protein Coding	33	GC12P043795	7.87
GOSR1	Golgi SNAP Receptor Complex Member 1	Protein Coding	40	GC17P030477	7.86
OPA3	Outer Mitochondrial Membrane Lipid Metabolism Regulator OPA3	Protein Coding	40	GC19M045527	7.86
SCN4A	Sodium Voltage-Gated Channel Alpha Subunit 4	Protein Coding	45	GC17M063938	7.86
GOLGA2	Golgin A2	Protein Coding	42	GC09M128255	7.85
MYC	MYC Proto-Oncogene, BHLH Transcription Factor	Protein Coding	53	GC08P127735	7.84
CYB5R3	Cytochrome B5 Reductase 3	Protein Coding	45	GC22M042617	7.83
SVIP	Small VCP Interacting Protein	Protein Coding	34	GC11M022799	7.82
NLRP1	NLR Family Pyrin Domain Containing 1	Protein Coding	45	GC17M005499	7.8
ZDHHC6	Zinc Finger DHHC-Type Palmitoyltransferase 6	Protein	36	GC10M112430	7.8

		Coding			
EEF2	Eukaryotic Translation Elongation Factor 2	Protein Coding	50	GC19M003976	7.79
TMEM259	Transmembrane Protein 259	Protein Coding	32	GC19M001010	7.79
TNFRSF1A	TNF Receptor Superfamily Member 1A	Protein Coding	51	GC12M006328	7.79
CDK5	Cyclin Dependent Kinase 5	Protein Coding	54	GC07M151053	7.78
DSPP	Dentin Sialophosphoprotein	Protein Coding	37	GC04P087608	7.78
EEF1A1	Eukaryotic Translation Elongation Factor 1 Alpha 1	Protein Coding	44	GC06M073515	7.77
NSFL1C	NSFL1 Cofactor	Protein Coding	40	GC20M001442	7.77
F8	Coagulation Factor VIII	Protein Coding	47	GC0XM154835	7.76
TMX3	Thioredoxin Related Transmembrane Protein 3	Protein Coding	39	GC18M068673	7.76
OXT	Oxytocin/Neurophysin I Prepropeptide	Protein Coding	41	GC20P003068	7.75
SLC2A1	Solute Carrier Family 2 Member 1	Protein Coding	54	GC01M042925	7.75
EIF2AK1	Eukaryotic Translation Initiation Factor 2 Alpha Kinase 1	Protein Coding	43	GC07M006022	7.74
ICAM1	Intercellular Adhesion Molecule 1	Protein	52	GC19P010270	7.73

		Coding			
CACNB4	Calcium Voltage-Gated Channel Auxiliary Subunit Beta 4	Protein Coding	47	GC02M151832	7.72
CDKAL1	CDK5 Regulatory Subunit Associated Protein 1 Like 1	Protein Coding	39	GC06P020534	7.72
FMR1	FMRP Translational Regulator 1	Protein Coding	46	GC0XP147912	7.72
FURIN	Furin, Paired Basic Amino Acid Cleaving Enzyme	Protein Coding	48	GC15P090868	7.72
PLA2G6	Phospholipase A2 Group VI	Protein Coding	50	GC22M045345	7.7
COPA	COPI Coat Complex Subunit Alpha	Protein Coding	42	GC01M160288	7.69
PTGIS	Prostaglandin I2 Synthase	Protein Coding	48	GC20M049503	7.68
MAPKAPK2	MAPK Activated Protein Kinase 2	Protein Coding	50	GC01P206684	7.67
SSR3	Signal Sequence Receptor Subunit 3	Protein Coding	38	GC03M156540	7.67
TFEB	Transcription Factor EB	Protein Coding	43	GC06M041835	7.66
CYP1A2	Cytochrome P450 Family 1 Subfamily A Member 2	Protein Coding	47	GC15P074748	7.65
DUSP19	Dual Specificity Phosphatase 19	Protein Coding	40	GC02P183078	7.64
DNAJB2	DnaJ Heat Shock Protein Family (Hsp40) Member B2	Protein	44	GC02P219279	7.62

		Coding			
HLA-DRA	Major Histocompatibility Complex, Class II, DR Alpha	Protein Coding	48	GC06P032439	7.62
HSPH1	Heat Shock Protein Family H (Hsp110) Member 1	Protein Coding	44	GC13M031134	7.62
TMCC1	Transmembrane And Coiled-Coil Domain Family 1	Protein Coding	35	GC03M129647	7.62
PSMD2	Proteasome 26S Subunit, Non-ATPase 2	Protein Coding	44	GC03P184298	7.61
DHCR7	7-Dehydrocholesterol Reductase	Protein Coding	47	GC11M071428	7.59
DPM2	Dolichyl-Phosphate Mannosyltransferase Subunit 2, Regulatory	Protein Coding	37	GC09M127935	7.59
TGFB3	Transforming Growth Factor Beta 3	Protein Coding	48	GC14M075958	7.59
APAF1	Apoptotic Peptidase Activating Factor 1	Protein Coding	48	GC12P098645	7.57
CALR3	Calreticulin 3	Protein Coding	39	GC19M016450	7.57
COL9A2	Collagen Type IX Alpha 2 Chain	Protein Coding	43	GC01M040300	7.57
MYH6	Myosin Heavy Chain 6	Protein Coding	47	GC14M023380	7.57
CAPN1	Calpain 1	Protein Coding	51	GC11P065198	7.56
PRDX1	Peroxiredoxin 1	Protein	51	GC01M045511	7.56

		Coding			
GORASP2	Golgi Reassembly Stacking Protein 2	Protein Coding	41	GC02P170928	7.55
NOTCH3	Notch Receptor 3	Protein Coding	50	GC19M015159	7.55
SEC31B	SEC31 Homolog B, COPII Coat Complex Component	Protein Coding	38	GC10M100486	7.54
AQP11	Aquaporin 11	Protein Coding	36	GC11P077589	7.53
ICMT	Isoprenylcysteine Carboxyl Methyltransferase	Protein Coding	43	GC01M006222	7.53
MCL1	MCL1 Apoptosis Regulator, BCL2 Family Member	Protein Coding	49	GC01M150673	7.53
SKP1	S-Phase Kinase Associated Protein 1	Protein Coding	44	GC05M134148	7.53
SOX9	SRY-Box Transcription Factor 9	Protein Coding	48	GC17P072121	7.53
FLNB	Filamin B	Protein Coding	46	GC03P058008	7.52
JPH1	Junctophilin 1	Protein Coding	40	GC08M074234	7.52
EGR1	Early Growth Response 1	Protein Coding	45	GC05P138465	7.51
SDF2L1	Stromal Cell Derived Factor 2 Like 1	Protein Coding	37	GC22P023730	7.51
ECPAS	Ecm29 Proteasome Adaptor And Scaffold	Protein	28	GC09M111361	7.5

		Coding			
CCND1	Cyclin D1	Protein Coding	53	GC11P069641	7.49
CDH2	Cadherin 2	Protein Coding	51	GC18M027950	7.49
FASLG	Fas Ligand	Protein Coding	49	GC01P172628	7.49
YOD1	YOD1 Deubiquitinase	Protein Coding	38	GC01M207044	7.49
MAP2K1	Mitogen-Activated Protein Kinase Kinase 1	Protein Coding	55	GC15P066386	7.48
PRKCA	Protein Kinase C Alpha	Protein Coding	52	GC17P066302	7.47
ALOX15	Arachidonate 15-Lipoxygenase	Protein Coding	45	GC17M004630	7.46
EPO	Erythropoietin	Protein Coding	43	GC07P100720	7.46
SGTA	Small Glutamine Rich Tetratricopeptide Repeat Containing Alpha	Protein Coding	39	GC19M002754	7.44
TLR2	Toll Like Receptor 2	Protein Coding	52	GC04P153684	7.44
GOLPH3	Golgi Phosphoprotein 3	Protein Coding	41	GC05M032124	7.43
RSAD2	Radical S-Adenosyl Methionine Domain Containing 2	Protein Coding	40	GC02P006865	7.43
GP6	Glycoprotein VI Platelet	Protein	45	GC19M055013	7.42

		Coding			
TTPA	Alpha Tocopherol Transfer Protein	Protein Coding	42	GC08M063048	7.42
CPQ	Carboxypeptidase Q	Protein Coding	35	GC08P096645	7.41
KPNB1	Karyopherin Subunit Beta 1	Protein Coding	44	GC17P047649	7.41
EIF4E	Eukaryotic Translation Initiation Factor 4E	Protein Coding	51	GC04M098871	7.4
PPP1CA	Protein Phosphatase 1 Catalytic Subunit Alpha	Protein Coding	50	GC11M067415	7.4
FOXRED2	FAD Dependent Oxidoreductase Domain Containing 2	Protein Coding	34	GC22M036487	7.39
KRT14	Keratin 14	Protein Coding	49	GC17M041582	7.39
UBXN8	UBX Domain Protein 8	Protein Coding	32	GC08P030723	7.39
CTSD	Cathepsin D	Protein Coding	54	GC11M001752	7.38
HM13	Histocompatibility Minor 13	Protein Coding	39	GC20P031514	7.38
PKD1	Polycystin 1, Transient Receptor Potential Channel Interacting	Protein Coding	46	GC16M002170	7.38
DSG3	Desmoglein 3	Protein Coding	40	GC18P031447	7.36
COL9A3	Collagen Type IX Alpha 3 Chain	Protein	43	GC20P062816	7.35

		Coding			
PRDM10	PR/SET Domain 10	Protein Coding	35	GC11M129899	7.35
APOA4	Apolipoprotein A4	Protein Coding	43	GC11M116820	7.33
FKBP5	FKBP Prolyl Isomerase 5	Protein Coding	47	GC06M041289	7.33
MATN1	Matrilin 1	Protein Coding	39	GC01M030711	7.32
PTH	Parathyroid Hormone	Protein Coding	48	GC11M013492	7.32
ELAVL1	ELAV Like RNA Binding Protein 1	Protein Coding	43	GC19M007958	7.31
M6PR	Mannose-6-Phosphate Receptor, Cation Dependent	Protein Coding	44	GC12M008951	7.31
SERPINA2	Serpin Family A Member 2 (Gene/Pseudogene)	Protein Coding	27	GC14M094363	7.31
AIF1	Allograft Inflammatory Factor 1	Protein Coding	41	GC06P033396	7.3
MT-TK	Mitochondrially Encoded TRNA-Lys (AAA/G)	RNA Gene	15	GCMTP008297	7.29
SFTPC	Surfactant Protein C	Protein Coding	44	GC08P022156	7.27
TPP1	Tripeptidyl Peptidase 1	Protein Coding	44	GC11M006614	7.27
USP19	Ubiquitin Specific Peptidase 19	Protein Coding	40	GC03M049205	7.25

ATXN2	Ataxin 2	Protein Coding	47	GC12M111443	7.24
CDC42	Cell Division Cycle 42	Protein Coding	53	GC01P022043	7.24
GPER1	G Protein-Coupled Estrogen Receptor 1	Protein Coding	39	GC07P001143	7.24
PRDX5	Peroxiredoxin 5	Protein Coding	48	GC11P064317	7.22
U2AF1	U2 Small Nuclear RNA Auxiliary Factor 1	Protein Coding	42	GC21M043092	7.22
PXN	Paxillin	Protein Coding	47	GC12M120210	7.21
SHISA5	Shisa Family Member 5	Protein Coding	36	GC03M048468	7.21
BRCA1	BRCA1 DNA Repair Associated	Protein Coding	52	GC17M043044	7.2
SHC1	SHC Adaptor Protein 1	Protein Coding	47	GC01M154962	7.2
HEXA	Hexosaminidase Subunit Alpha	Protein Coding	47	GC15M072340	7.19
OSBPL3	Oxysterol Binding Protein Like 3	Protein Coding	39	GC07M024836	7.19
COL10A1	Collagen Type X Alpha 1 Chain	Protein Coding	44	GC06M116118	7.18
QDPR	Quinoid Dihydropteridine Reductase	Protein Coding	50	GC04M017460	7.18

SET	SET Nuclear Proto-Oncogene	Protein Coding	49	GC09P128684	7.18
BET1	Bet1 Golgi Vesicular Membrane Trafficking Protein	Protein Coding	38	GC07M093962	7.17
CES1	Carboxylesterase 1	Protein Coding	48	GC16M055836	7.17
LPIN1	Lipin 1	Protein Coding	48	GC02P011649	7.17
RAB2A	RAB2A, Member RAS Oncogene Family	Protein Coding	43	GC08P060516	7.17
SCFD1	Sec1 Family Domain Containing 1	Protein Coding	38	GC14P030622	7.17
EMD	Emerin	Protein Coding	47	GC0XP154379	7.16
NPC1	NPC Intracellular Cholesterol Transporter 1	Protein Coding	48	GC18M023506	7.15
STBD1	Starch Binding Domain 1	Protein Coding	37	GC04P076306	7.15
UBE2G1	Ubiquitin Conjugating Enzyme E2 G1	Protein Coding	44	GC17M004270	7.15
CYB5A	Cytochrome B5 Type A	Protein Coding	45	GC18M074250	7.14
MAPK8IP1	Mitogen-Activated Protein Kinase 8 Interacting Protein 1	Protein Coding	45	GC11P045961	7.14
SELENOF	Selenoprotein F	Protein Coding	27	GC01M086863	7.14

CYB5R4	Cytochrome B5 Reductase 4	Protein Coding	40	GC06P083859	7.13
DMPK	DM1 Protein Kinase	Protein Coding	49	GC19M045769	7.12
SPAST	Spastin	Protein Coding	41	GC02P032063	7.12
CTSK	Cathepsin K	Protein Coding	50	GC01M150796	7.11
G6PC3	Glucose-6-Phosphatase Catalytic Subunit 3	Protein Coding	42	GC17P044070	7.1
VDAC1	Voltage Dependent Anion Channel 1	Protein Coding	47	GC05M133975	7.1
FPR2	Formyl Peptide Receptor 2	Protein Coding	47	GC19P051752	7.09
SERPINE1	Serpin Family E Member 1	Protein Coding	52	GC07P101127	7.09
COG7	Component Of Oligomeric Golgi Complex 7	Protein Coding	40	GC16M023307	7.08
SGPP2	Sphingosine-1-Phosphate Phosphatase 2	Protein Coding	37	GC02P222424	7.08
CAV3	Caveolin 3	Protein Coding	44	GC03P008733	7.07
DPAGT1	Dolichyl-Phosphate N-Acetylglucosaminephosphotransferase 1	Protein Coding	46	GC11M119096	7.06
RCN1	Reticulocalbin 1	Protein Coding	40	GC11P032090	7.06

CTSB	Cathepsin B	Protein Coding	52	GC08M011842	7.05
NCK2	NCK Adaptor Protein 2	Protein Coding	43	GC02P105744	7.05
PTK2	Protein Tyrosine Kinase 2	Protein Coding	49	GC08M140657	7.05
SEC11A	SEC11 Homolog A, Signal Peptidase Complex Subunit	Protein Coding	38	GC15M084669	7.05
SRPRB	SRP Receptor Subunit Beta	Protein Coding	38	GC03P133784	7.05
PSMA5	Proteasome 20S Subunit Alpha 5	Protein Coding	44	GC01M109399	7.04
STIP1	Stress Induced Phosphoprotein 1	Protein Coding	44	GC11P064203	7.04
CLGN	Calmegin	Protein Coding	39	GC04M140388	7.03
PTEN	Phosphatase And Tensin Homolog	Protein Coding	54	GC10P087863	7.03
ZC3H12A	Zinc Finger CCCH-Type Containing 12A	Protein Coding	37	GC01P037474	7.03
HSD17B12	Hydroxysteroid 17-Beta Dehydrogenase 12	Protein Coding	41	GC11P043702	7.02
RCN2	Reticulocalbin 2	Protein Coding	40	GC15P076931	7.02
AFP	Alpha Fetoprotein	Protein Coding	47	GC04P073431	7.01

UBAC2	UBA Domain Containing 2	Protein Coding	38	GC13P099200	7.01
FN1	Fibronectin 1	Protein Coding	51	GC02M215360	7
RELA	RELA Proto-Oncogene, NF-KB Subunit	Protein Coding	51	GC11M065653	7
MMP2	Matrix Metallopeptidase 2	Protein Coding	55	GC16P055390	7
PIGN	Phosphatidylinositol Glycan Anchor Biosynthesis Class N	Protein Coding	41	GC18M061905	7
TMED7	Transmembrane P24 Trafficking Protein 7	Protein Coding	33	GC05M115613	7
