# Supplementary material for: Loss of BAP31 Is Detrimentally Aging Photoreceptors Through ER Stress-Mediated Retinal Degeneration
Source: Cells. 2025 Nov 17;14(22):1802. doi: 10.3390/cells14221802 (PMC12650883; doi:10.3390/cells14221802)
Supplement: Supplementary file 1 [file cells-14-01802-s001.zip › Supplement-figure.pdf]

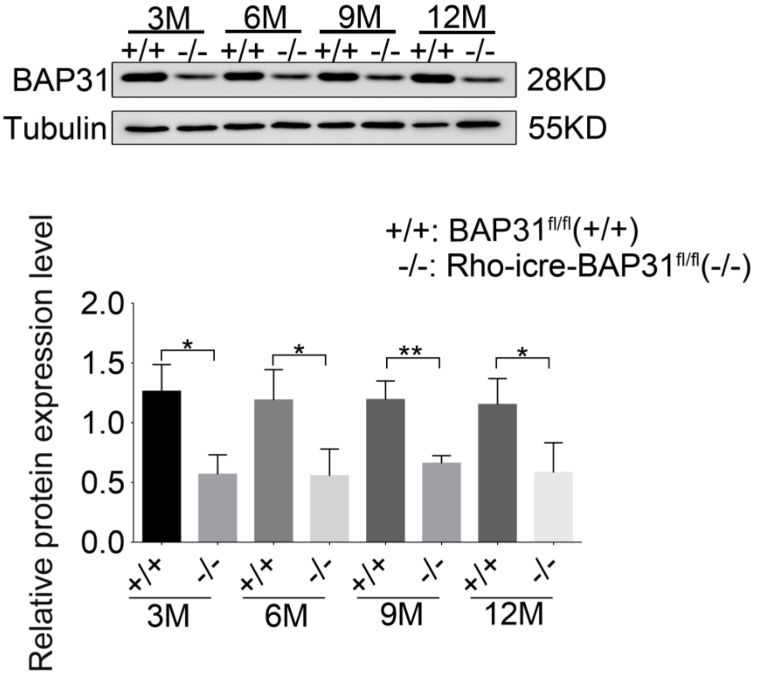

Figure S1 Deletion of BAP31 in Mouse Rod Photoreceptors via Rho-iCre. Western blot analysis was performed to assess the expression level of BAP31 in the retinas of $BAP31^{fl/fl}$(+/+) mice and Rho-iCre-$BAP31^{fl/fl}$(-/-) mice at the age of 3, 6, 9, 12 months. (n=3). *P < 0.05, **P < 0.01.

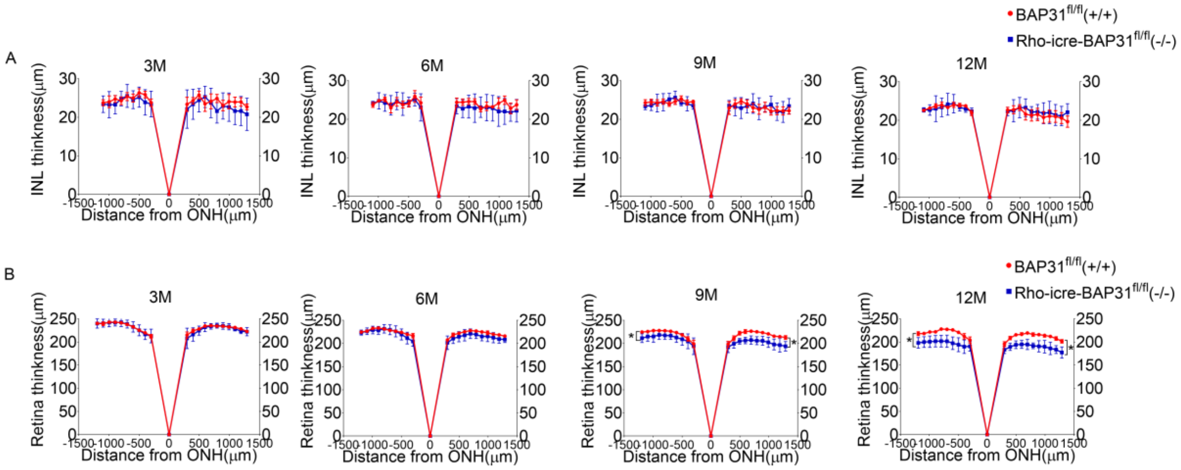

Figure S2 BAP31 specific knockdown results in Age-Related Degeneration. (A) INL thickness was quantified in both BAP31$^{fl/fl}$(+/+) mice and Rho-iCre-BAP31$^{fl/fl}$(-/-) mice at the age of 3, 6, 9, 12 months. n=8 for BAP31$^{fl/fl}$(+/+) mice and Rho-iCre-BAP31$^{fl/fl}$(-/-) at each time group. (B) Retina thickness was quantified in both BAP31$^{fl/fl}$(+/+) mice and Rho-iCre-BAP31$^{fl/fl}$(-/-) mice at the age of 3, 6, 9, 12 months. (n=8). *P < 0.05, **P < 0.01.

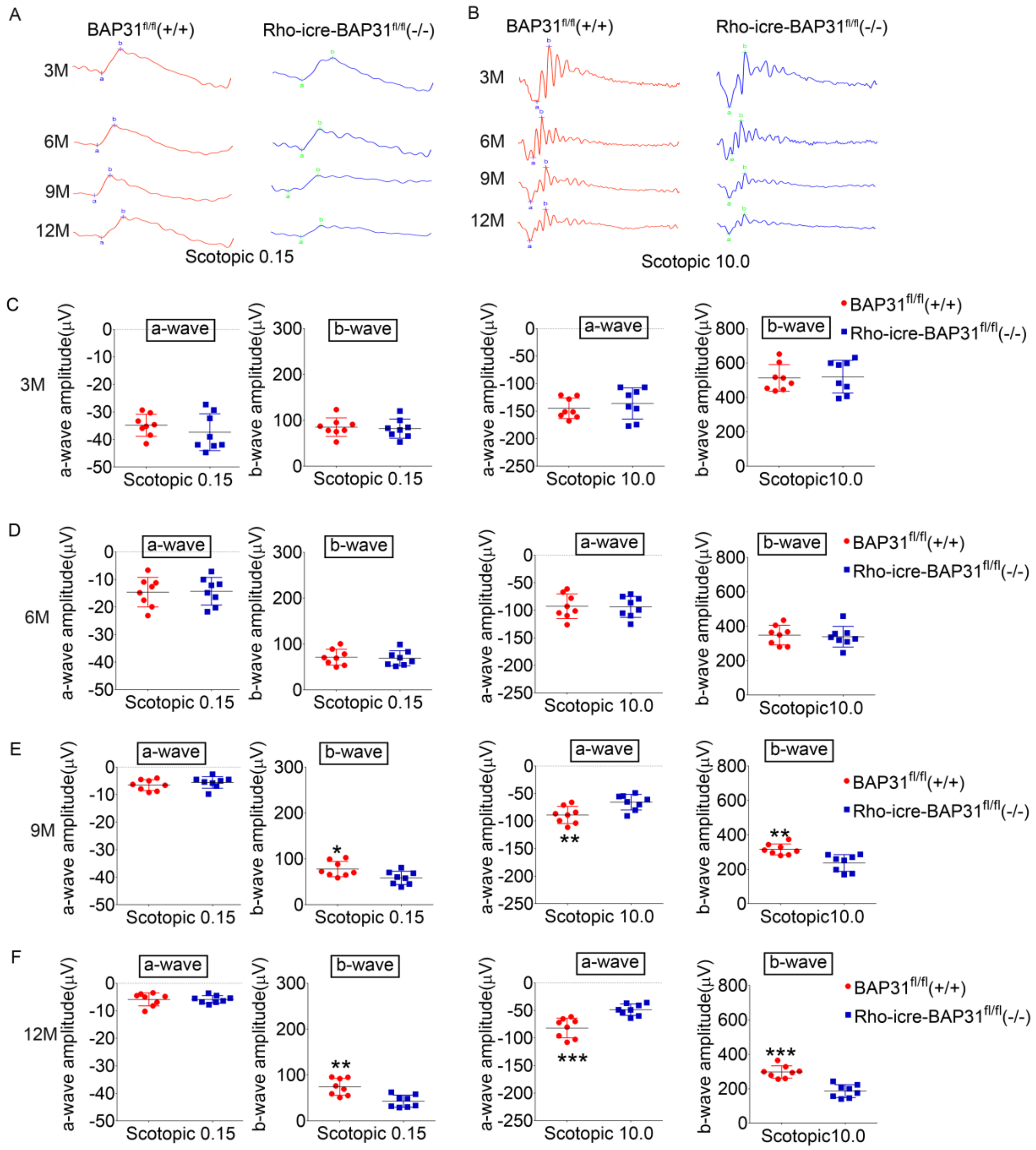

Figure S3 Electroretinography (ERG) recording analysis under 0.15 cd • s/m^2 and 10 cd • s/m^2 . The ERG waveforms were recorded of retinas of BAP31$^{fl/fl}$(+/+) mice and Rho-iCre-BAP31$^{fl/fl}$(-/-) mice at 3, 6, 9, and 12 months of age under 0.15 flash strength(cd·s/m^2) (A) and 10.0 flash strength(cd·s/m^2) (B) (n = 8). (C-F) Quantitative analysis of a-wave and b-wave amplitudes was conducted at flash strengths of 0.15 and 10.0 cd·s/m^2. Statistical comparisons were performed to assess potential differences between the genotypes. *P < 0.05, **P < 0.01, *** P < 0.001.

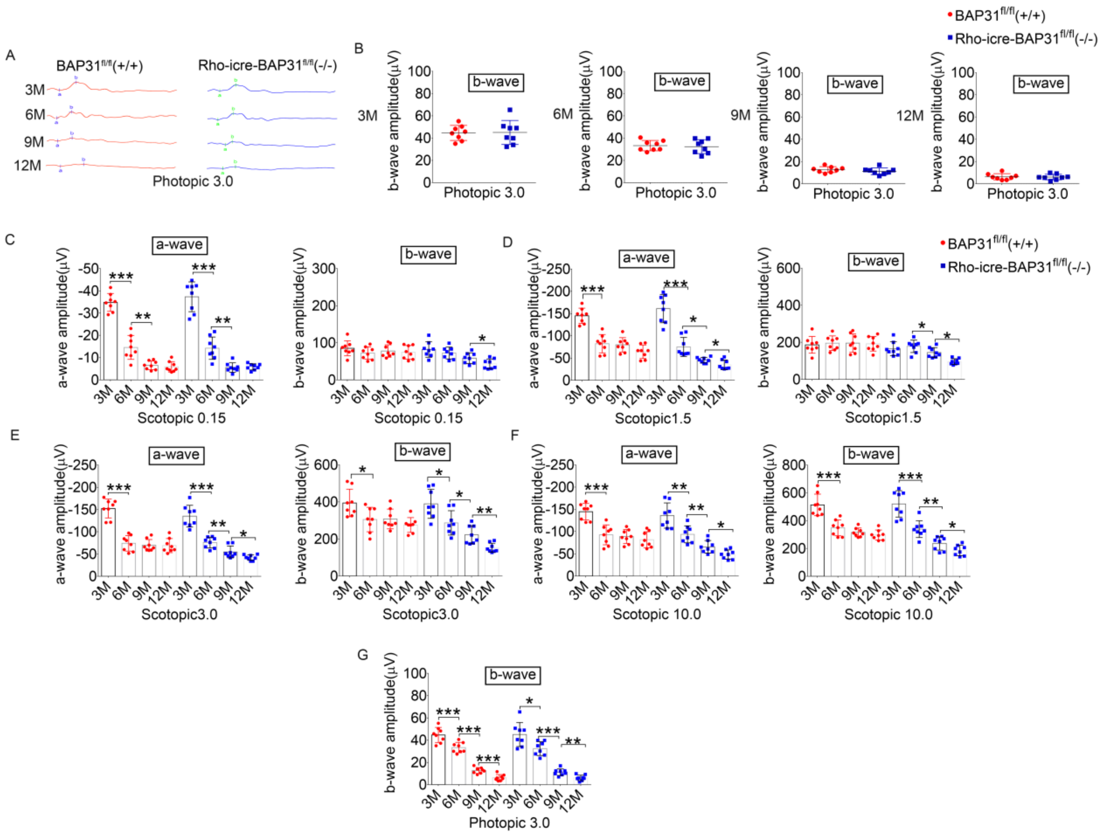

FigureS4 Electroretinography (ERG) recording analysis. (A) The ERG waveforms were recorded of retinas of BAP31$^{fl/fl}$(+/+) mice and Rho-iCre-BAP31$^{fl/fl}$(-/-) mice at 3, 6, 9 and 12 months of age under 3.0 flash strength(cd·s/m^2) of photopic ERG (n=8). (B) Quantitative analysis of b-wave amplitudes(n=8). (C) Statistical comparison of ERG a-wave and b-wave at each time point under scotopic 0.15 cd·s/m² of BAP31$^{fl/fl}$(+/+) mice and Rho-iCre-BAP31$^{fl/fl}$(-/-) mice at 3, 6, 9 and 12 months of age(n=8). (D) Statistical comparison of ERG a-wave and b-wave at each time point under scotopic 1.5 cd·s/m² of BAP31$^{fl/fl}$(+/+) mice and Rho-iCre-BAP31$^{fl/fl}$(-/-) mice at 3, 6, 9 and 12 months of age(n=8). (E) Statistical comparison of ERG a-wave and b-wave at each time point under scotopic 3.0 cd·s/m² of BAP31$^{fl/fl}$(+/+) mice and Rho-iCre-BAP31$^{fl/fl}$(-/-) mice at 3, 6, 9 and 12 months of age(n=8). (F) Statistical comparison of ERG a-wave and b-wave at each time point under scotopic 10.0 cd·s/m² of BAP31$^{fl/fl}$(+/+) mice and Rho-iCre-BAP31$^{fl/fl}$(-/-) mice at 3, 6, 9 and 12 months of age (n=8) (n=8). (G) Statistical comparison of ERG b-wave at each time point under photopic 3.0 cd·s/m² of BAP31$^{fl/fl}$(+/+) mice and Rho-iCre-BAP31$^{fl/fl}$(-/-) mice at 3, 6, 9 and 12 months of age (n=8). *P < 0.05, **P < 0.01, *** P < 0.001.

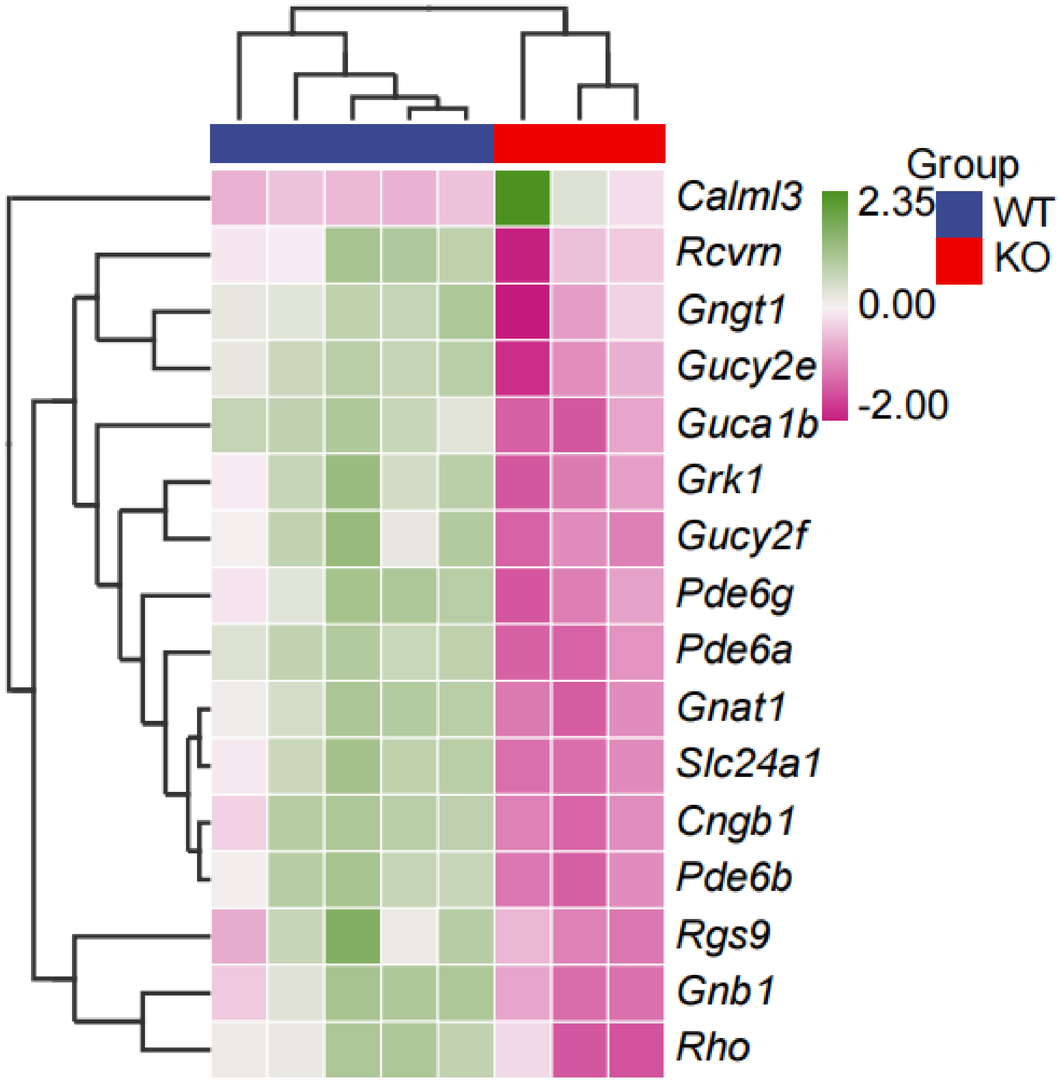

FigureS5 Heatmap of gene expression in the phototransduction pathway (BAP31$^{fl/fl}$(+/+) =5, Rho-iCre-BAP31$^{fl/fl}$(-/-) =3).

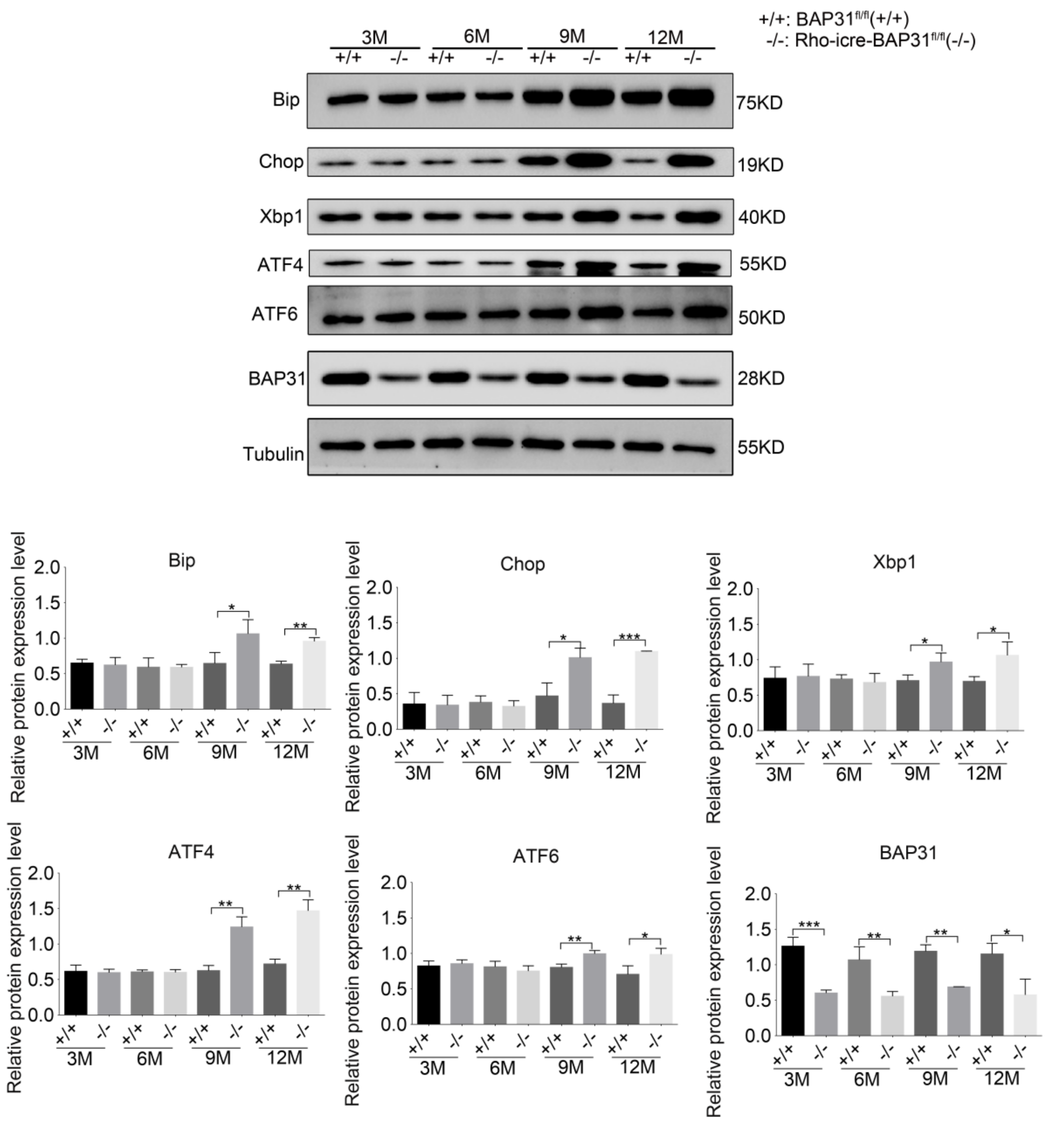

Figure S6 BAP31 regulated UPR to affect the expression of phototransduction genes. Western blot analysis was performed to assess the expression levels of the UPR genes of BAP31$^{fl/fl}$(+/+) mice and Rho-iCre-BAP31$^{fl/fl}$(-/-) mice at 3, 6, 9 and 12 months of age(n=3). *P < 0.05, **P < 0.01, *** P < 0.001.
