# Supplementary figures and images for: Loss of BAP31 Is Detrimentally Aging Photoreceptors Through ER Stress-Mediated Retinal Degeneration
Source: Cells. 2025 Nov 17;14(22):1802. doi: 10.3390/cells14221802 (PMC12650883; doi:10.3390/cells14221802)

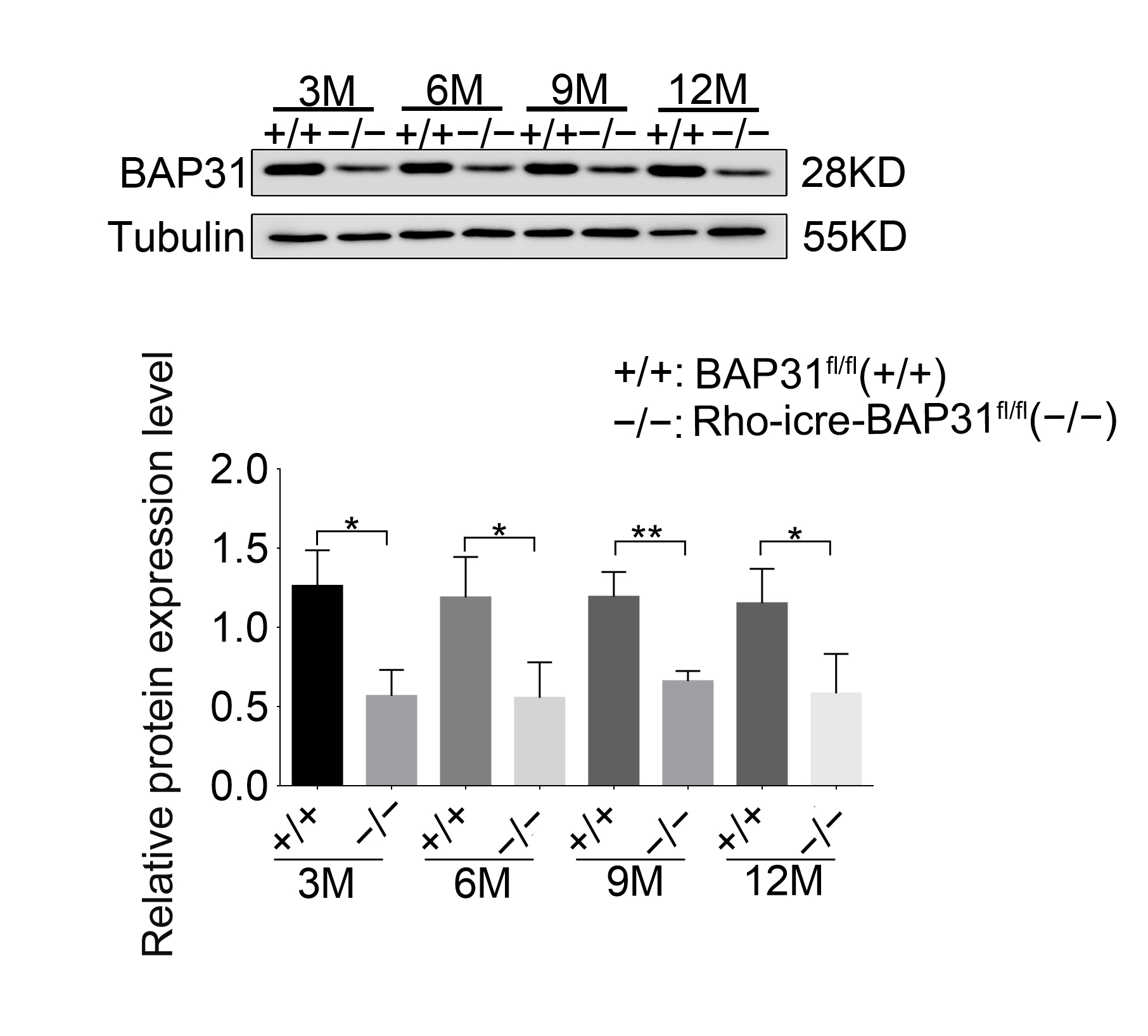

Supplement: Supplementary file 1 [file cells-14-01802-s001.zip › FigureS1.JPG]

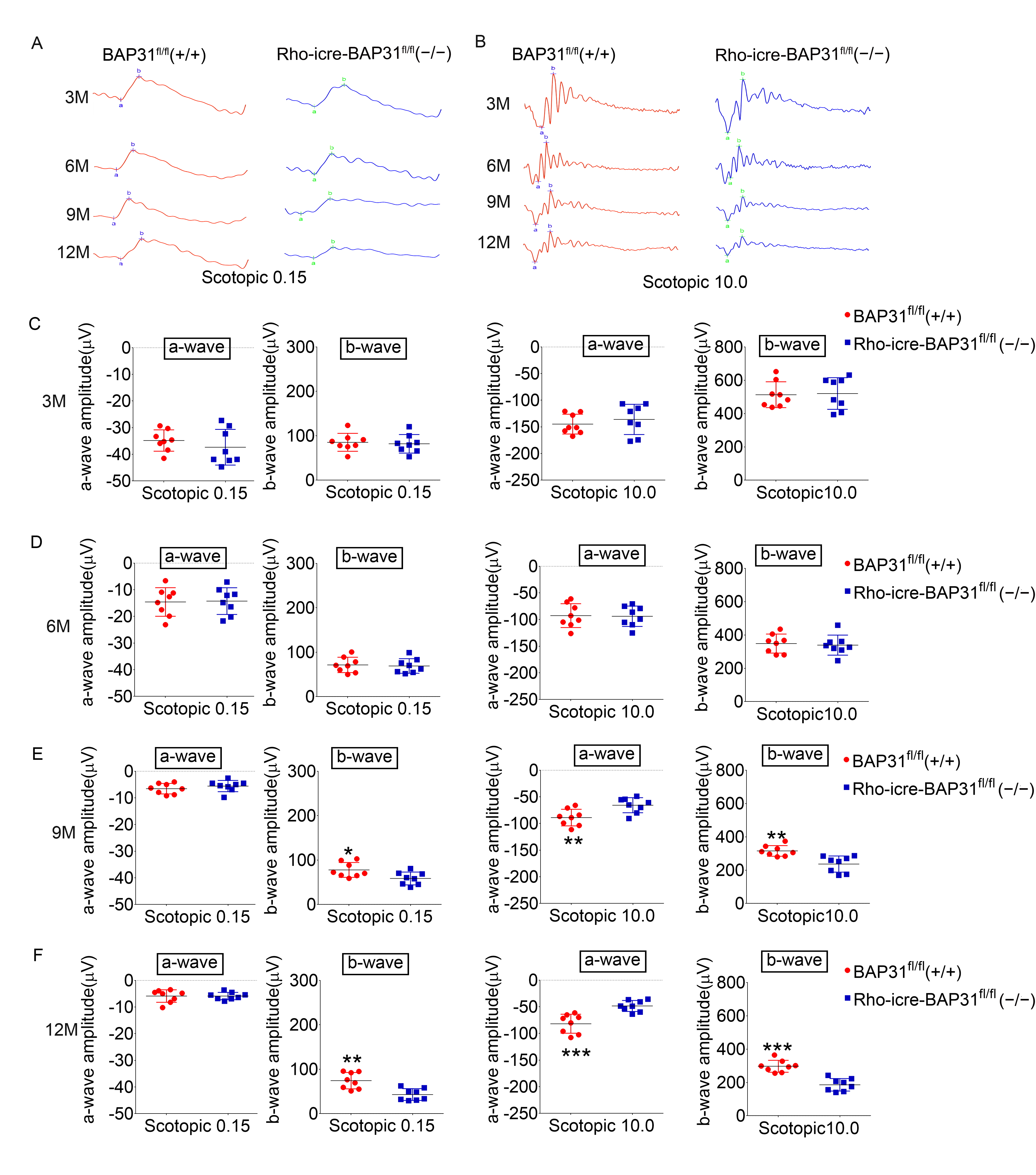

Supplement: Supplementary file 1 [file cells-14-01802-s001.zip › FigureS3.JPG]

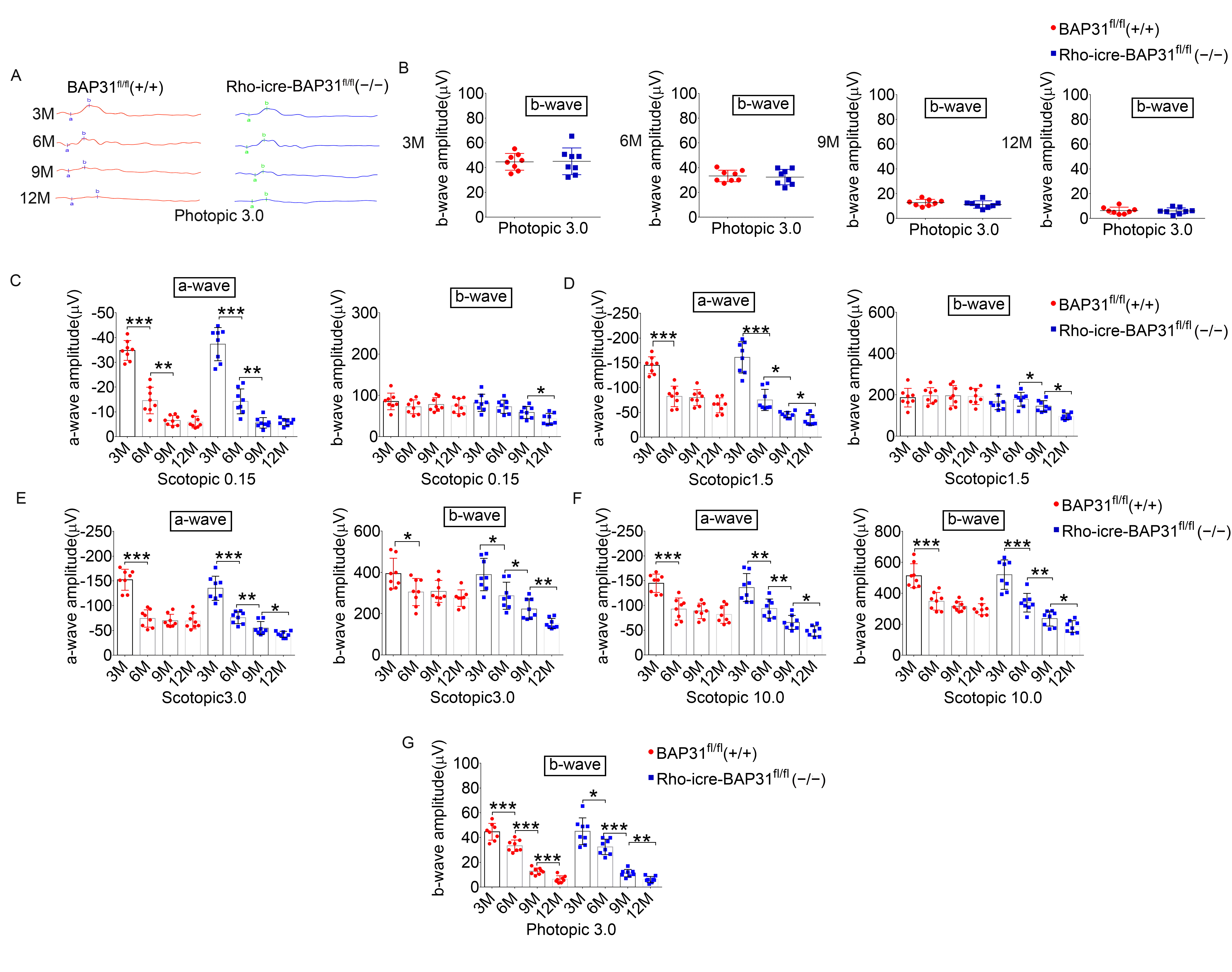

Supplement: Supplementary file 1 [file cells-14-01802-s001.zip › FigureS4.JPG]

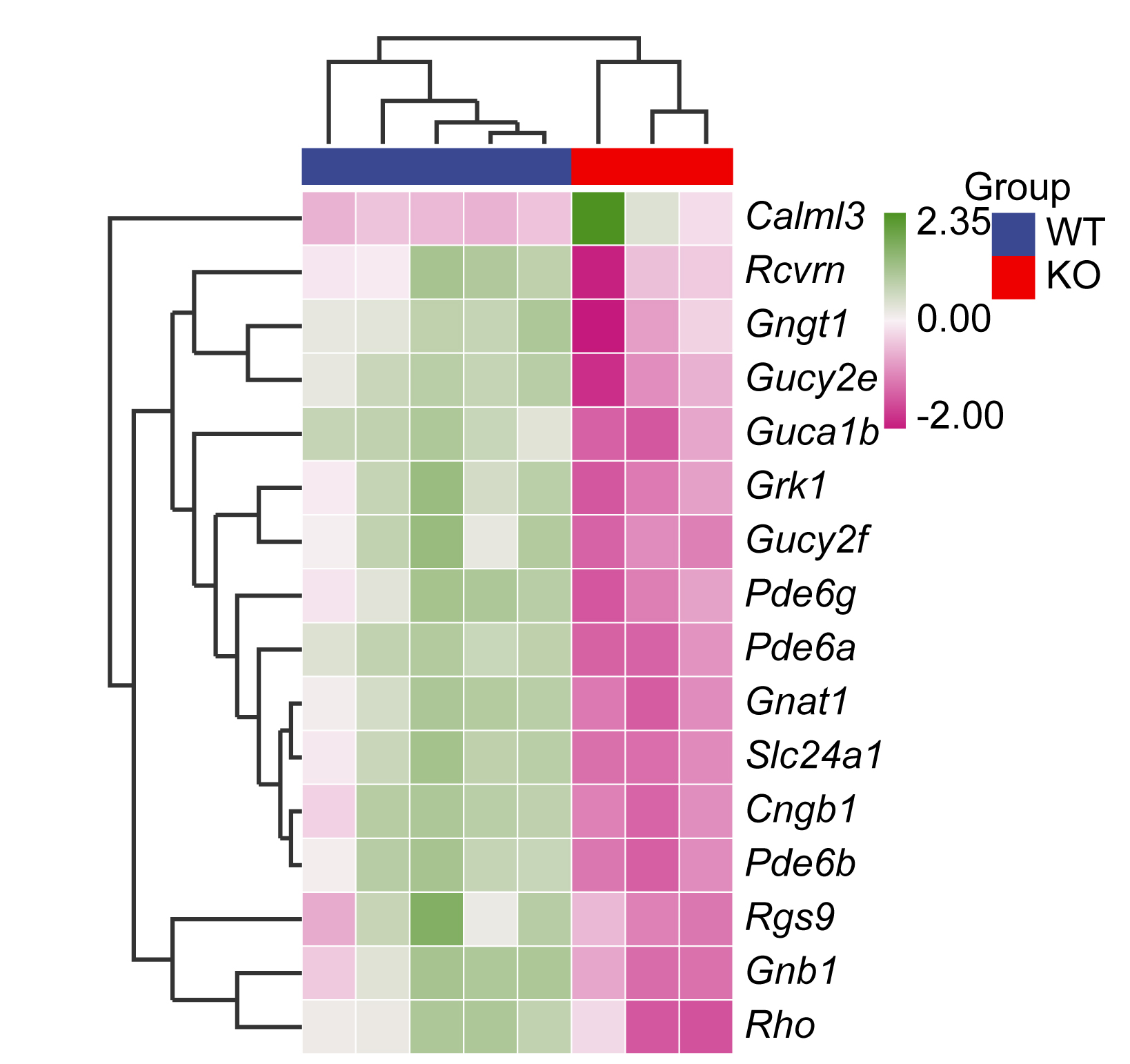

Supplement: Supplementary file 1 [file cells-14-01802-s001.zip › FigureS5.jpg]

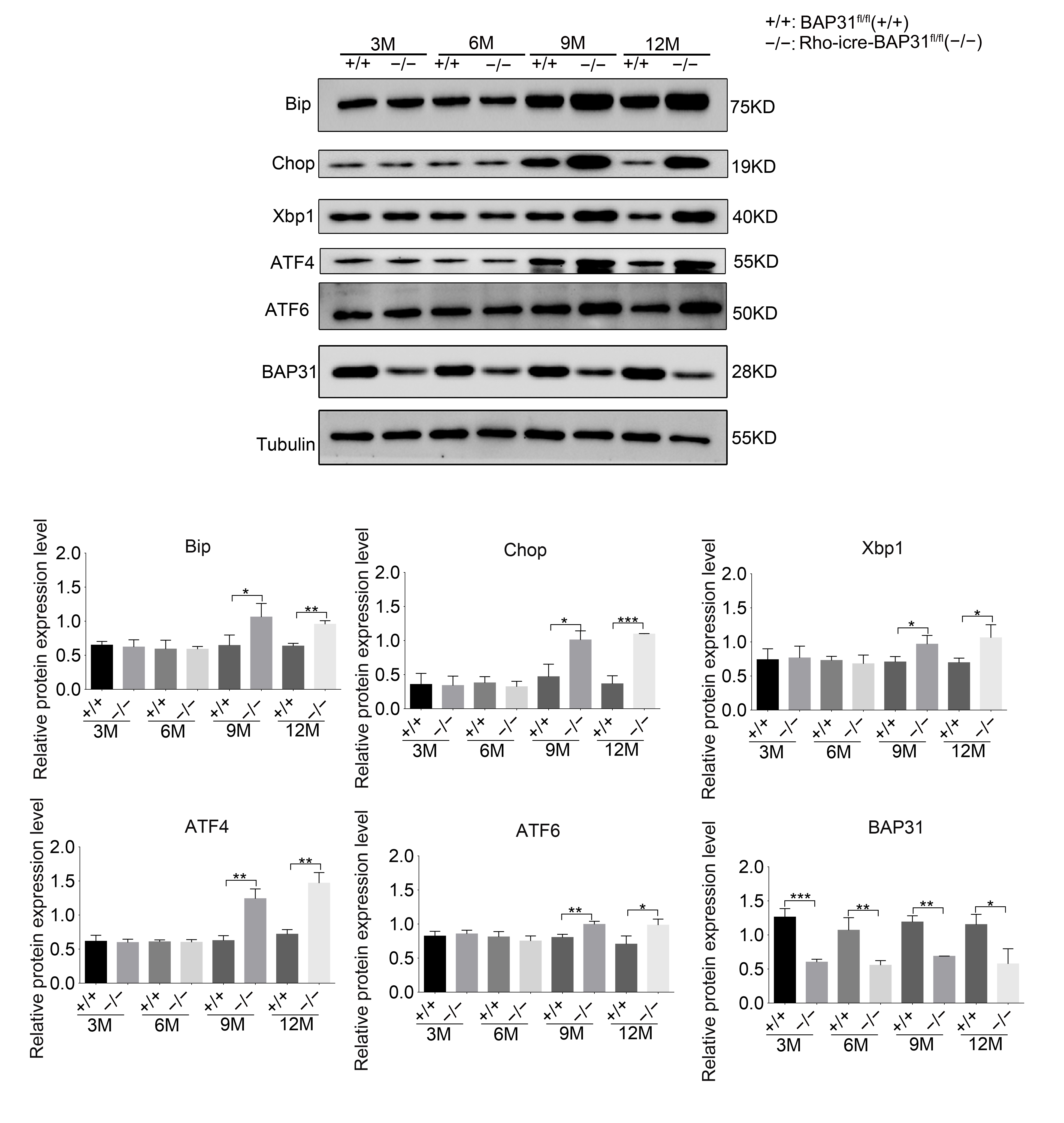

Supplement: Supplementary file 1 [file cells-14-01802-s001.zip › FigureS6.JPG]

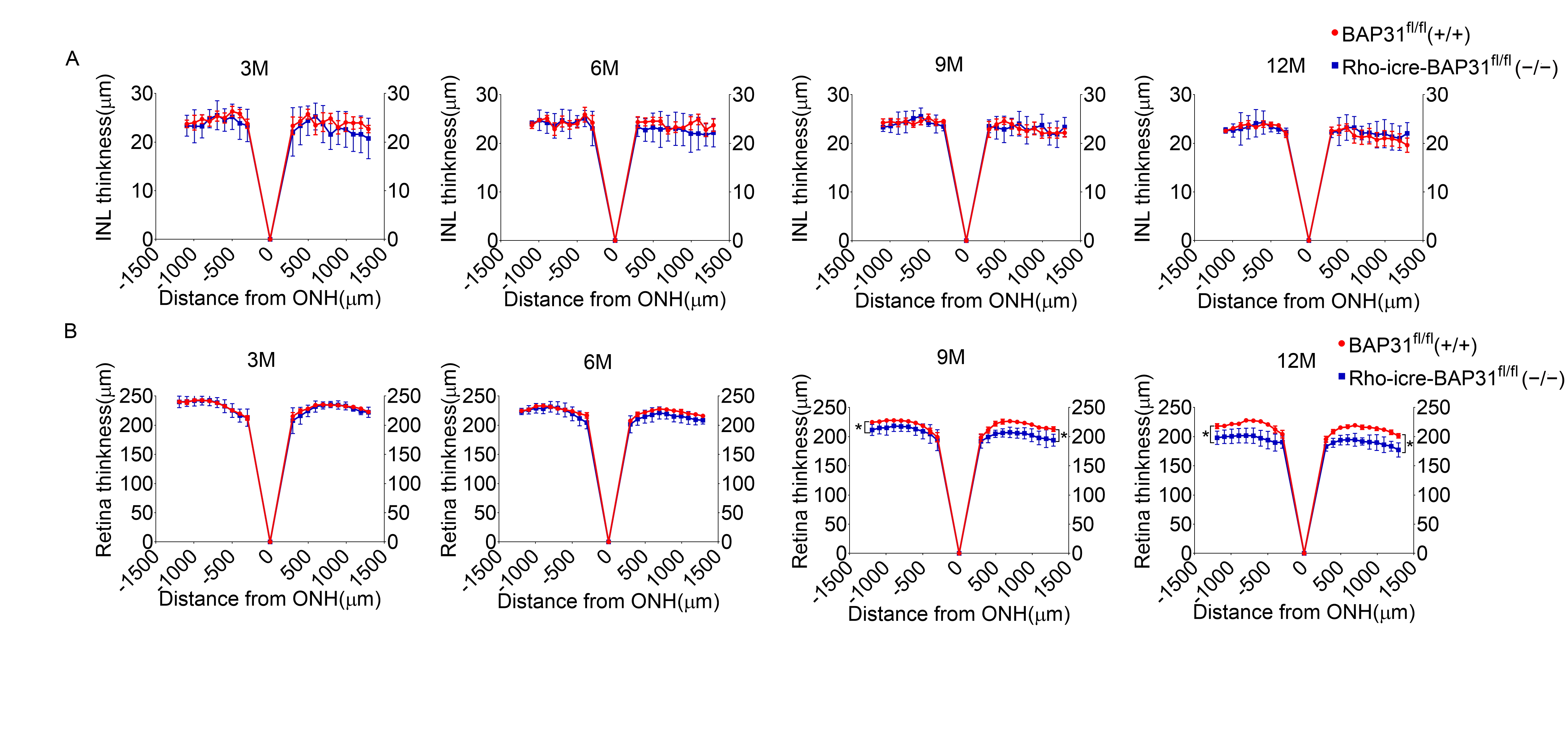

Supplement: Supplementary file 1 [file cells-14-01802-s001.zip › FigureS2.JPG]
